# Supplementary material for: Intensification of Double Kinetic Resolution of Chiral Amines and Alcohols via Chemoselective Formation of a Carbonate–Enzyme Intermediate
Source: Molecules. 2022 Jul 6;27(14):4346. doi: 10.3390/molecules27144346 (PMC9319036; doi:10.3390/molecules27144346)
Supplement: Supplementary file 1 [file molecules-27-04346-s001.zip › molecules-1785567-supplementary.pdf]

Intensification of double kinetic resolution of chiral amines and alcohols via chemoselective formation of a carbonate–enzyme intermediate

Supplementary Materials

Table of Contents:

General procedure for the synthesis of alcohols 1e,f,i,j 3

General procedure for the synthesis of carbamates 3

Enzymatic kinetic resolution of alcohols 4

Double enzymatic kinetic resolution by alcoholysis of carbonate: 9

Double enzymatic kinetic resolution by aminolysis of carbonate: 13

Upscale studies 22

Spectral and analytical data 24

NMR spectra, HPLC and GC data 32

General procedure for the synthesis of alcohols 1e, f, I ,j

General procedure for the reduction of ketones to alcohols (1e, 1f, 1i):

Ketone (10 mmol) was dissolved in 10 mL of methanol in a round bottom flask, and the resulting solution was stirred in an ice bath for 10 minutes. Subsequently, sodium borohydride (13 mmol) was added slowly, and the reaction mixture was stirred at room temperature for 4 h. Then, the reaction mixture was poured into 50 mL of ice-cooled saturated aqueous solution of NH_4Cl and extracted with DCM (3x20mL). The combined organic phases were dried over anhydrous $MgSO_4$, and the solvent was removed on a rotary evaporator. The crude product was purified using column chromatography (hexane/ethyl acetate) [39].

Compound **1j** was obtained via a two-step reduction from 4-phenylbut-3-en-2-one: 4-phenylbut-3-en-2-one (10 mmol) was put in a round bottom flask and dissolved in 10 mL of anhydrous ethanol and 3 mL of glacial acetic acid. Then, 10 mg of Pd/C catalyst was added and the mixture was stirred under an atmosphere of hydrogen overnight, at room temperature. The reaction mixture was then filtrated through celite, to remove the palladium catalyst. Solvents were evaporated on a rotary evaporator. Subsequently, the obtained liquid was dissolved in 10 mL of methanol for further reduction with 13 mmol of $NaBH_4$, according to the general method.

Additionally, enantiomerically-enriched alcohols (See sections Double enzymatic kinetic resolution by alcoholysis of carbonate and Double enzymatic kinetic resolution by aminolysis of carbonate) isolated from enzyme-catalyzed reactions were characterized by ^{1}H NMR and ^{13}C NMR. The obtained spectral data were compared to the literature. Retention times were measured according to the methods mentioned in the Materials and methods and Enantioselectivity determination sections.

General procedure for the synthesis of carbamates

Amine (5 mmol) was dissolved in 6 mL of dry THF, and 7.5 mmol of TEA (triethylamine) was added. The mixture was stirred in an ice bath for ten minutes. Then chloroformate (5.5 mmol) was added at 0 °C. The reaction was stirred at room temperature for two hours then poured on 20 mL of saturated NH_4Cl solution and extracted with DCM (3 × 20mL). The combined organic phases were dried over anhydrous $MgSO_4$, and the solvent was evaporated to dryness. The residue was purified using column chromatography (hexane/ethyl acetate), to give pure carbamate [40].

Enzymatic kinetic resolution of alcohols:

Enzyme screening:

Enzymes: Novozym 435 CALB (*Candida antarctica* lipase B, activity: 10000 PLU/g), PPL (crude lipase type II), wheat germ lipase, Amano PS, Amano AK, Acylase from *Aspergillus meleus*, Lipozyme, crude Lipase from *Candida antarctica*, lipase from *Rhizopus arrizus*, and lipase from *Candida cylindracea*; Immobilized enzymes: Tl imino (*Thermomyces lanuginosa* lipase), PS imino (*Pseudomonas cepacia* lipase), CalA imino (*Candida antarctica* lipase A), CR imino (*Candida rugosa* lipase), and RM imino (*Rhizomucor miehei* lipase) were used in screening for the activity in the reaction of mixed carbonate synthesis. To a 3-mL glass vial containing 1-phenyethanol (0.26 mmol) were put dimethyl carbonate (1.25 mmol), 1 mL of toluene, and enzyme (50 mg). The reaction vial was placed on a shaker (200 rpm, r.t.) for 16 h. Conversions were calculated using GC measurements (sample preparation: 50 µL of reaction mixture dissolved in 1 mL of ethyl acetate). The obtained results showed that of all tested enzymes only Novozym 435 (CALB) catalyzed the studied transformation.

Carbonate to alcohol ratio optimization

The studies on various ratios between substrates (performed on model substrates) showed that 5 equivalents of carbonate were optimal (when higher excesses were used, the conversion increased slightly (1% between 5 eq and 10 eq) (Table S1). To ensure that the mixed carbonate was synthesized exclusively due to the chemoselectivity of the enzyme, and not the chemical equilibrium, studies with an excess of alcohol were performed of the model reaction. 1-phenyl ethanol was added at 2, 5, and 10 molar equivalents to carbonate. In every reaction, asymmetric carbonate was synthesized exclusively; however, the yields were lower than in the situation of carbonate excess. However, only (*R*)-carbonate was synthesized with >99 % *ee*.

Table S1. Optimal carbonate to alcohol ratio for EKR of alcohols performed on model substrates in MTBE.

Used carbonate	Carbonate to alcohol ratio	Reaction time (h)	conversion (%)[a]	*ee*S[b] (%)	*ee*P[c] (%)
DMC	1:1	16	18	22	>99
DMC	2:1	16	22	27	>99
DMC	5:1	16	27	32	>99
DMC	10:1	16	28	35	>99
DMC	1:2	16	22	26	>99
DMC	1:5	16	22	27	>99
DMC	1:10	16	22	26	>99

[a] Obtained from GC. [b] Enantiomeric excess of 1-phenylethanol. [c] Enantiomeric excess of methyl 1-phenylethyl carbonate.

Optimization of reaction conditions:

Under the initial conditions (room temperature, shaker 200 rpm), different solvents were examined. In a 3-mL vial, 1-phenyethanol (0.26 mmol) and dimethyl carbonate (1.25 mmol) were dissolved in 1 mL of solvent, enzyme was added (50 mg), and the reaction vial was placed on a shaker (200 rpm, r.t.) for 16 h. Conversions were calculated using GC measurements (sample preparation: 50 µL of reaction mixture dissolved in 1 mL of ethyl acetate). Reaction without solvent was performed on 1 mmol of 1-phenylethanol and 5 mmol of dimethyl

carbonate, according to the abovementioned procedure. The results of solvent screening are presented in Table S2.

Table S2. Solvent screening for EKR of alcohols for model substrates.

Solvent	Reaction time (h)	conversion (%)[a]	*ee*S[b] (%)	*ee*P[c] (%)
neat	16	20	22	>99
toluene	16	21	24	>99
dry toluene	16	21	24	>99
MTBE	16	27	32	>99
DCM	16	0	-	-
cyclohexane	16	0	-	-
hexane	16	19	20	>99
heptane	16	0	-	-
diethyl ether	16	8	9	>99
isooctane	16	0	-	-
THF	16	0	-	-
dioxane	16	0	-	-
DMSO	16	0	-	-

[a] Obtained from GC. [b] Enantiomeric excess of 1-phenylethanol. [c] Enantiomeric excess of methyl 1-phenylethyl carbonate.

Afterwards, temperature screening was performed for the model reaction. In a 3-mL vial, 1-phenyethanol (0.26 mmol) and dimethyl carbonate (1.25 mmol) were dissolved in 1 mL of MTBE. Then, enzyme was added (50 mg) and the reaction was placed on a shaker (200 rpm). Conversions were calculated using GC measurements (sample preparation: 50 µL of reaction mixture dissolved in 1 mL of ethyl acetate) after 20 h (Table S3).

Table S3. Enantioselectivity studies of EKR of alcohols using carbonates.

Temperature (°C)	Reaction time (h)	Conversion (%) [a]	*ee*S_1[b] (%)	*ee*P_1[c] (%)
20	20	24	28	>99
25	20	27	32	>99
30	20	21	26	>99
40	20	24	29	>99

[a] Obtained from GC. [b] Enantiomeric excess of 1-phenylethanol. [c] Enantiomeric excess of methyl 1-phenylethyl carbonate.

Under the optimized conditions, scope and limitation studies were performed using dimethyl and diethyl carbonates as acyl donors, to determine the impact of the acyl donors on the conversion of alcohol to carbonate (Table S4). For a model reaction, the time course (Figure S1) was recorded. To a 3-mL glass vial containing alcohol (**1a–n**) (0.26 mmol), DMC or DEC (1.25 mmol), 1 mL of MTBE, and enzyme were added (50 mg). Conversions were calculated using GC measurements (sample preparation: 50 µL of reaction mixture was dissolved in 1 mL of ethyl acetate). The velocity of reaction was defined as the molar amount of carbonate product after a particular reaction time.

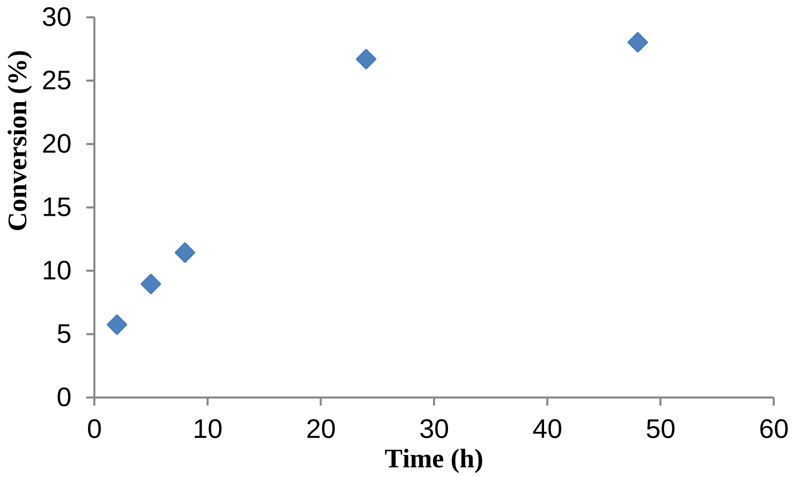

Figure S1. Time course for model reaction of carbonate synthesis.

Table S4. E-values for scope limitation reactions.

Carbonate (substrate)[a]	Acyl donor	Reaction time (h)	C (%)[b]	v (mmol/h)	*ee*S1[c] (%)	*ee*P1[d] (%)	E[e]
**1a**	DMC	24	31	0.0034	40%	>99%	>200
**1a**	DEC	24	15	0.0016	17	>99%	>200
**1b**	DMC	120	3	0.0001	2	>99%	>200
**1b**	DEC	120	0	0.0000	-	-	-
**1c**	DMC	24	13	0.0014	14	>99	>200
**1d**	DMC	24	60	0.0065	6	4	1
**1d**	DEC	24	59	0.0064	0	0	-
**1e**	DMC	24	14	0.0015	16 %	>99%	-
**1f**	DMC	24	37	0.0040	52 %	>99%	>200
**1g**	DMC	24	44	0.0048	45 %	55%	5
**1h**	DMC	24	48	0.0052	84 %	>99%	>200
**1h**	DEC	24	47	0.0051	78 %	>99%	>200
**1i**	DMC	48	35	0.0019	67 %	>99%	>200
**1j**	DMC	48	48	0.0026	81 %	>99%	>200
**1k**	DMC	24	23	0.0025	31 %	>99%	>200
**1l**	DMC	24	55	0.0060	8	7	1
**1m**	DMC	24	87	0.0094	0	0	-
**1n**	DMC	24	93	0.0101	-	-	-

[a] According to description in Figure 1. [b] GC conversion (C) and reaction velocity (v) calculated using carbonate and alcohol peaks,$v_1=C_1 \times n_{0S1} \times t^{-1}$ while t—reaction time, n_{0S1}—amount of alcohol added to the reaction vial. [c] Enantiomeric excess of alcohol. [d] Enantiomeric excess of the obtained carbonate. [e] E value calculated for reaction of carbonate synthesis, according to the general equation.

Enantiospecificity studies

To determine the enantiospecificity of the established method, studies on the racemization of the product and reaction with different enantiomers were performed. Under optimized conditions, in-3 mL vial, alcohol (1—racemic, 2—*R*-enantiomer, 3—*S*-enantiomer) (0.26 mmol) and dimethyl carbonate (1.25 mmol) were dissolved in 1 mL of MTBE, enzyme was added (50 mg), and the reaction was placed on a shaker (200 rpm, r.t.). Conversions were calculated using GC measurements (sample preparation: 50 µL of reaction mixture was dissolved in 1 mL of ethyl acetate) after 1, 2, 10, 20 days. When (*S*)-1-phenylethanol was used as substrate, conversion was not observed. For (*R*)-1-phenylethanol, conversion to product was observed and the reaction reached a final conversion of 73%, a racemate reaction was observed as mentioned in the scope and limitation studies, and the final conversion was 39% (after 20 days); however, product was (*R*)-carbontae >99 % *ee* and racemization was not observed.

Double enzymatic kinetic resolution by alcoholysis of carbonate:

Enzyme screening

In a 3-mL vial, model substrates, 2-hexanol (0.26 mmol) and methyl 1-phenylethyl carbonate (0.26 mmol), were dissolved in 1 mL of toluene, and enzymes: (Novozym 435 CALB (*Candida antarctica* lipase B, activity: 10000 PLU/g), PPL (crude lipase type II), wheat germ lipase, Amano PS, Amano AK, Acylase from *Aspergillus meleus*, Lipozyme, crude Lipase from *Candida antarctica*, lipase from *Rhizopus arrizus*, lipase from *Candida cylindracea*; and immobilized enzymes: Tl imino (*Thermomyces lanuginosa* lipase), PS imino (*Pseudomonas cepacia* lipase), CalA imino (*Candida antarctica* lipase A), CR imino (*Candida rugosa* lipase), RM imino (*Rhizomucor miehei* lipase),) were added (50 mg), and the reaction was placed on a shaker (200 rpm, r.t.). Reaction conversions were calculated using GC measurements (sample preparation was as mentioned in the section "Enzymatic kinetic resolution").

Solvent optimization:

In a 3-mL vial, 2-hexanol (0.26 mmol) and methyl 1-phenylethyl carbonate (0.26 mmol) were dissolved in 1 mL of solvent, and Novozym 435 CALB was added (50 mg), and then the reaction was placed on a shaker (200 rpm, r.t.). Conversions were calculated using GC measurements (sample preparation was as mentioned in the section "Enzymatic kinetic resolution") after 96 h. The obtained results are gathered in Table S5.

Table S5. Solvent screening for DEKR by carbonate alcoholysis.

Solvent	Reaction time (h)	conversion (%)	$ee\mathbf{S_1}$[a]	$ee\mathbf{P_1}$[b]
toluene	96	46	73	92
dry toluene	96	43	74	92
MTBE	96	47	67	77
- (neat)	96	0.0	0	0
DCM	96	0.0	0	0
cyclohexane	96	11	11	89
hexane	96	28	38	92
heptane	96	21	24	90
diethyl ether	96	34	44	84
isooctane	96	11	12	88
THF	96	0.0	0	0
dioxane	96	0.0	0	0
DMSO	96	0.0	0	0

[a] Enantiomeric excess of methyl 1-phenylethyl carbonate, [b] enantiomeric excess of 1-phenylethanol,

General procedure for temperature screening: In a 3-mL vial, 2-hexanol (0.26 mmol) and methyl 1-phenylethyl carbonate (0.26 mmol) were dissolved in 1 mL of toluene, Novozym 435 CALB was added (50 mg), and the reaction was placed on a shaker (200 rpm) and conversions were calculated using GC measurements (sample preparation was as mentioned in the section "Enzymatic kinetic resolution") after 96 h (Table S6). The highest conversion was obtained for the temperature of 60 °C; however, the enantioselectivity of the reaction was poor. The reaction velocities of the double enzymatic kinetic resolution were defined as the amount of product

formed (alcohol or carbamate) in the reaction mixture. Due to the convergence of DEKR step conversions, the reactions velocities were similar.

Table S6. Temperature optimization for DEKR by carbonate alcoholysis.

Temperature (°C)	Reaction time (h)	conversion (%)	***ee*S$_1$**[a] (%)	***ee*P$_1$**[b] (%)	***ee*P$_2$**[c] (%)
20	96	12	16	97	>99
25	96	13	18	96	>99
30	96	17	19	96	>99
40	96	41	66	94	>99
50	96	45	74	93	>99
60	96	50	87	88	97

[a] Enantiomeric excess of methyl (*S*)-1-phenylethyl carbonate. [b] Enantiomeric excess of (*R*)-1-phenylethanol. [c] Enantiomeric excess of (*R*)-methyl 2-hexyl carbonate.

A conversion vs. enzyme amount plot was obtained at the optimal temperature of 50 °C, according to the general procedure, including the amounts of substrates and solvents. Reactions were analyzed by GC after 96 h. The results are gathered in Figure S2.

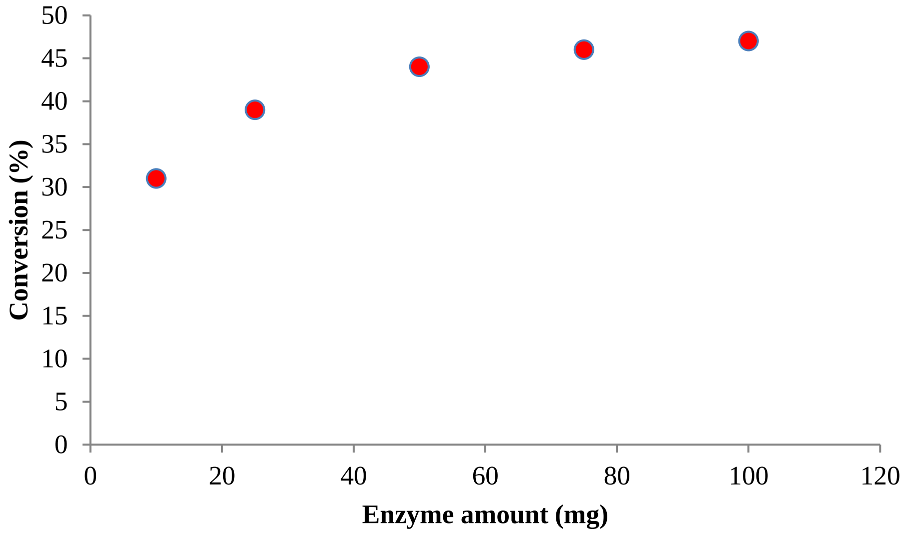

Figure S2. Enzyme amount optimization for DEKR by carbonate alcoholysis.

The time course of model reaction (Figure S3) was recorded for the model reaction.

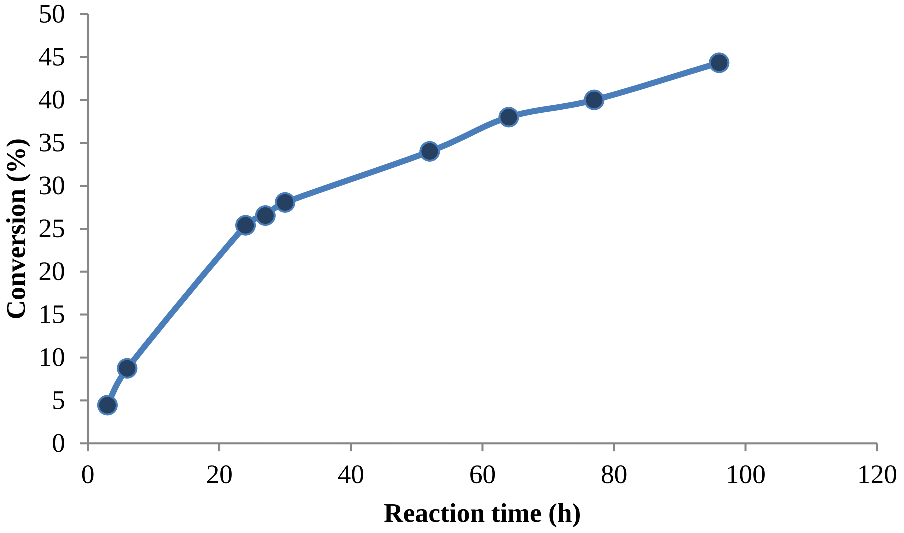

Figure S3. Time course of model DEKR by carbonate alcoholysis reaction under optimized conditions.

Alcoholysis procedure (scope and limitation and alcoholysis with methanol and ethanol):

In a 3-mL glass vial, carbonate (rac **2/3a**) (0.26 mmol), alcohol (rac **1b-n**), MeOH, or EtOH (0.26 mmol) were dissolved, and 1 mL of toluene and Novozym 435® CALB (50 mg) were added. The vial was closed and placed on a shaker (200 rpm, 50 °C) for 96 h. Then 50 µL of reaction mixture was dissolved in 1 mL of ethyl acetate, for GC studies. The rest of the mixture was purified by column chromatography (hexane:ethyl acetate), and the obtained conversions and enantioselectivities are presented in Table 2.

Double enzymatic kinetic resolution by aminolysis of carbonate:

Enzyme screening

Based on the previous literature on enzyme-catalyzed kinetic resolutions, [41] we used 1-phenylethylamine and methyl 1-phenylethyl carbonate as model substrates. In a 3-mL vial, 1-phenylethylamine (0.26 mmol) and methyl 1-phenylethyl carbonate (0.26 mmol) were dissolved in 1 mL of toluene, and the enzymes (Novozym 435 CALB (*Candida antarctica* lipase B, activity: 10000 PLU/g), PPL (crude lipase type II), wheat germ lipase, Amano PS, Amano AK, Acylase from *Aspergillus meleus*, Lipozyme, crude Lipase from *Candida antarctica*, lipase from *Rhizopus arrizus*, lipase from *Candida cylindracea*, Immobilized enzymes: Tl imino (*Thermomyces lanuginosa* lipase), PS imino (*Pseudomonas cepacia* lipase), CalA imino (*Candida antarctica* lipase A), CR imino (*Candida rugosa* lipase), RM imino (*Rhizomucor miehei* lipase) were added (50 mg), and then the reaction was placed on a shaker (200 rpm, r.t.). Reaction conversions were calculated using GC measurements (sample preparation was as mentioned in the section Enzymatic kinetic resolution).

Solvent optimization:

In a 3-mL vial, 1-phenylethylamine (0.26 mmol) and methyl 1-phenylethyl carbonate (0.26 mmol) were dissolved in 1 mL of solvent, Novozym 435 CALB was added (50 mg), and the reaction was placed on a shaker (200 rpm, r.t.). Conversions were calculated using GC measurements (sample preparation was as mentioned in the section Enzymatic kinetic resolution) after 16 hours. The obtained results are gathered in Table S7.

Table S7. Solvent screening for DEKR of carbonates and 1-phenylethyamine.

Solvent	Reaction time (h)	conversion (%)	*ee*S1[a]	*ee*P1[b]
toluene	16	11.9	12	97
dry toluene	16	9.6	9	77
MTBE	16	15.6	11	63
- (neat)	16	0.0	0	0
DCM	16	0.0	0	0
cyclohexane	16	9.5	10	77
hexane	16	12.3	12	83
heptane	16	10.0	12	83
diethyl ether	16	9.5	7	69
isooctane	16	0.0	0	0
THF	16	0.0	0	0
dioxane	16	0.0	0	0
DMSO	16	0.0	0	0

[a] Enantiomeric excess of methyl 1-phenylethyl carbonate, [b] enantiomeric excess of 1-phenylethanol,

Temperature and enzyme amount optimization, reaction time course, and enzyme recyclability:

General procedure for temperature screening: In a 3-mL vial, 1-phenyetylamine (0.26 mmol) and methyl 1-phenylethyl carbonate (0.26 mmol) were dissolved in 1 mL of toluene, Novozym 435 CALB was added (50 mg), and the reaction was placed on a shaker (200 rpm), and conversions were calculated using GC measurements (sample preparation was as mentioned in the section "Enzymatic kinetic resolution") after 36 h (Table S8 and Figure S4). The reaction velocities of the double enzymatic kinetic resolution were defined as the amount of product formed (alcohol or carbamate) in the reaction mixture.

Table S8. Temperature optimization.

Temperature (°C)	Reaction time (h)	conversion (%)	*ee*S1[a] (%)	*ee*P1[b] (%)	*ee*P2[c] (%)
20	36	17	98	18	>99
25	36	17	96	18	>99
30	36	17	96	19	>99
40	36	41	82	61	>99
50	36	48	87	85	>99
60	36	50	76	77	98

[a] Enantiomeric excess of (*S*)-methyl 1-phenylethyl carbonate. [b] Enantiomeric excess of (*R*)-1-phenylethanol. [c] Enantiomeric excess of (*R*)-methyl (1-phenylethyl) carbamate.

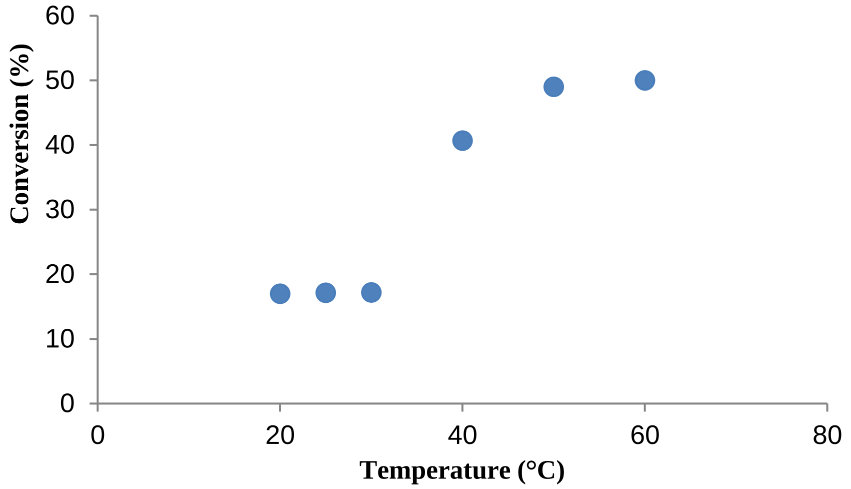

Figure S4. Temperature optimization for model reaction.

A conversion vs. enzyme amount plot was obtained at the optimal temperature of 50 °C, according to the general procedure, including the amounts of substrates and solvents. Reactions were analyzed by GC after 30 h. The results are gathered in Figure S5.

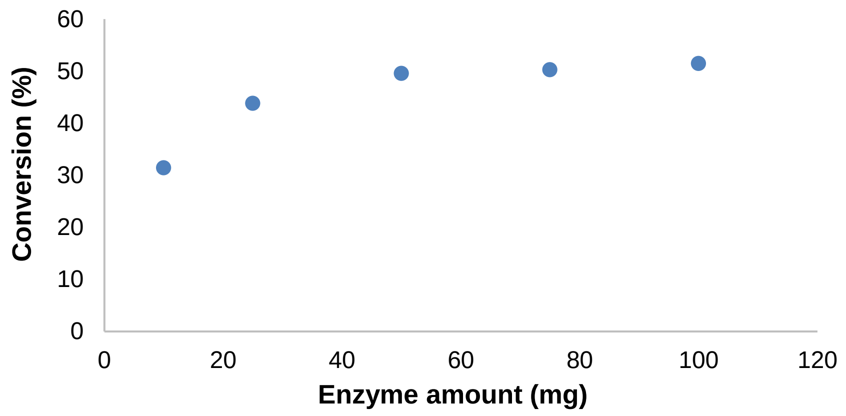

Figure S5. Enzyme amount optimization.

General procedure for enzyme recyclability studies: In a 3-mL vial, 1-phenylethylamine (0.26 mmol) and methyl 1-phenylethyl carbonate (0.26 mmol) were dissolved in 1 mL of toluene, Novozym 435 CALB was added (50 mg), and the reaction was placed on a shaker (200 rpm, 50 °C, 64 h) and conversions were calculated using GC measurements (Table S9 and Figure 2 in main text). Between every run, the enzyme was separated from the reaction mixture on a funnel and washed with toluene and MTBE before the next run.

Table S9. Enzyme recyclability studies.

Run number	Reaction time (h)	conversion (%)	*ee*S_1[a] (%)	*ee*P_1[b] (%)	*ee*P_2[c] (%)
1	30	44	76	93	>99
2	30	41	75	94	>99
3	30	40	66	91	>99
4	30	37	55	86	>99
5	30	25	50	84	>99

[a] Enantiomeric excess of (*S*)-methyl 1-phenylethyl carbonate. [b] Enantiomeric excess of (*R*)-1-phenylethanol. [c] Enantiomeric excess of (*R*)-methyl (1-phenylethyl) carbamate.

The time course of model reaction (Figure S6) was recorded under optimized conditions.

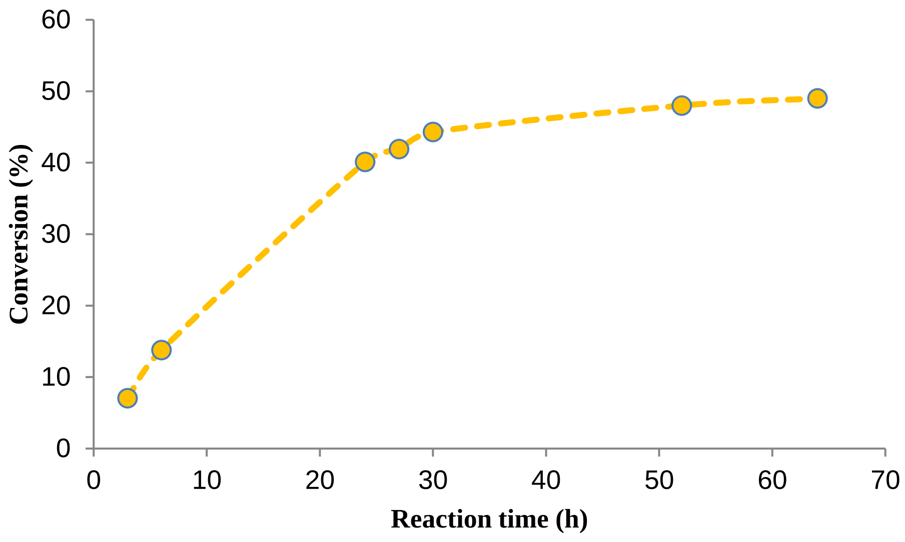

Figure S6. Time course of model reaction in optimized conditions.

Scope and limitation:

Under optimal conditions, scope limitation studies were performed. In a 3-mL glass vial, carbonate (0.26 mmol) and amine **1o–r** (0.26 mmol) were dissolved in 1 mL of toluene, and Novozyme 435® CALB (50 mg) was added. The vial was closed and placed on a shaker (200 rpm, 50 °C) for three days. Then, 50 µL of the reaction mixture was dissolved in 1 mL of ethyl acetate for GC studies. The rest of the mixture was purified by column chromatography (hexane:ethyl acetate) (Table S7). For all reactions, two E-values for the two steps of kinetic resolution were calculated using the general calculation method [41]. According to the limitations of the method accuracy, E-values above 200 were stated as “>200”. The optical purity of amines was calculated with optical rotation or upon derivation with acetyl group, according to the method of amine derivation and based on a literature procedure [42]. For reactions with benzylamine as acyl acceptor, conversion of the acylation of amine was determined using GC method 3. Method: column VF1701ms (Agilent Technologies) 30 m length, 0.25 mm cross-section, FID as detector with nitrogen as carrier gas, flow 1 mL/min, split 401, with a

temperature gradient 70–200 °C (rate 10 °C/min) and with a hold of 140 °C for 3 minutes and 200 °C for 2 minutes at the end of the run.

Table S10. E-values for scope limitation reactions with 1-phenylethylamine **1p**.

Carbonate (substrate)[a]	Reaction time (h)	C_1 (%)[b]	v_1 (mmol/h)[b]	C_2 (%)[c]	v_2 (mmol/h)[c]	*ee*S$_1$[d] (%)	*ee*S$_2$[e] (%)	*ee*P$_1$[f] (%)	*ee*P$_2$[g] (%)	Ec[h]	Ea[i]
**2a**	64	49	0.0020	47	0.0019	87	89	90	>99	54	>200
**3a**	64	27	0.0011	28	0.0011	34	36	82	85	14	18
**2b**	64	14	0.0006	13	0.0005	15	15	76	>99	8	>200
**3b**	88	24	0.0007	23	0.0007	20	10	62	33	5	2
**2c**	64	28	0.0011	29	0.0012	33	40	84	>99	16	>200
**2d**	64	49	0.0020	48	0.0020	24	91	25	>99	2	>200
**2e**	64	36	0.0019	36	0.0015	35	56	67	>99	7	>200
**2f**	64	49	0.0019	47	0.0019	89	89	89	>99	51	>200
**2g**	64	46	0.0017	44	0.0018	56	85	68	>99	8	>200
**2h**	64	47	0.0019	48	0.0020	90	92	93	>99	85	>200
**2i**	64	42	0.0017	43	0.0017	85	76	98	>99	>200	>200
**2j**	64	46	0.0019	45	0.0018	94	86	98	>99	>200	>200
**2k**	64	21	0.0009	21	0.0009	22	43	83	>99	13	>200
**2l**	64	44	0.0018	45	0.0018	29	81	44	>99	3	>200
**2m**	64	50	0.0020	50	0.0020	*nd*	95	*nd*	>99	*nd*	>200
**2n**	64	48	0.0020	49	0.0020	-	94	-	>99	-	>200

[a] According to the description in Figure 1. [b] GC conversion (C_1) and reaction velocity (v_1), calculated using carbonate and alcohol peaks,$v_1=C_1 \times n_{0S1} \times t^{-1}$ while t—reaction time, n_{0S1}—amount of carbonate added to the reaction vial. [c] GC conversion (C_2) and reaction velocity (v_2) calculated using amine and carbamate peaks, $v_2=C_2 \times n_{0S2} \times t^{-1}$ while t—reaction time, n_{0S2}—amount of carbonate added to the reaction vial. [d] Enantiomeric excess of (*S*)-carbonate. [e] Enantiomeric excess of (*S*)-amine (calculated after derivation mentioned in "Amine derivation" section). [f] Enantiomeric excess of (*R*)-alcohol. [g] Enantiomeric excess of (*R*)-methyl (1-phenylethyl) carbamate. [h] E value calculated for reaction of R_3OOCO- group removal from alcohol. [i] E value calculated for amine acylation.

Recovery yields for the obtained products

Recovery yield was examined for the model DEKR reaction (by aminolysis). In a glass vial were placed Novozyme 435® CALB (100 mg), carbonate (2 mmol), and 1-phenylethylamine (2 mmol), and 3 mL of toluene was added. The vial was closed and placed on a shaker (200 rpm, 50 °C) for three days. Then, 50 µL of reaction mixture was dissolved in 1 mL of ethyl acetate, for GC studies. Then, the reaction mixture was purified by column chromatography (hexane:ethyl acetate). The amount of obtained product was compared with that obtained according to the GC measurements. The results are presented in Table S11.

Table S11. Recovery yield studies for model DEKR reaction.

Compound[a]	GC yield (%)	Mass of product In mixture (mg)[b]	Yield of isolated product (%)	Mass of isolated product (mg)	Recovery (%)[c]
**(*S*)-2a**	55	213	53.9	209	98
**(*R*)-1a**	45	110	43.7	106.5	97

**(*S*)-1o**	56	157	63.7	154.2	98
**(*R*)-2o**	44	158	40.5	145	92

[a] According to description in Table 3, [b] Calculated using GC yield and amount of substrate used [c] Calculated with the equation: Recovery=$y_{isolated}/y_{GC}$ × 100%

Amine derivation:

To determine the optical purity of the amine obtained in DEKR, 1-phenylethylamine was acylated using acetic anhydride, according to the general method [42]. 1-phenylethyamine (0.1 mmol), DMAP (5 mg), and acetic anhydride (0.2 mmol) were dissolved in 500 µL of ethyl acetate (in Eppendorf®) and incubated on a shaker (200 rpm, r.t.) for four hours. Then, 500 µL of water was added to the reaction mixture and shook vigorously, to remove unreacted anhydride. Organic phase was dried with anhydrous $MgSO_4$ and diluted with 1 mL of ethyl acetate, before GC analysis (method 1).

Carbamate deprotection by hydrolysis

According to a procedure found in the literature [43], a carbamate group removal procedure was applied to obtain pure amine **1r,** to determine the enantioselectivity when separation of enantiomers by chiral HPLC was unsuccessful. Carbamate **3r** (0.1 mmol) was dissolved in 1 mL of a 1:1 mixture of dioxane and 1 M HCl and the reaction mixture was stirred in an ice bath. Then, the mixture was stirred at room temperature for 30 minutes. When the TLC suggested that the reaction was completed, dioxane was evaporated on a rotary evaporator and 1 mL of 2M NaOH aqueous solution was added. The resulting mixture was extracted with ethyl acetate (3 x 1 mL) and organic phases were combined, dried over anhydrous $MgSO_4$, and evaporated, to give pure amine.

Studies of DEKR with 4-phenylbutan-2-amine:

The DEKR studies performed with 4-phenylbutan-2-amine (instead of 1-phenylethylamine) gave the results presented in Table S12. The results indicated that a high enantioselectivity of the DEKR method is not only observed when 1-phenyletyl amine is used for the aminolysis. Upscale studies for the reaction between **2a** and 4-phenylbutan-2-amine (according to the method described in the **Upscale studies** section) resulted in a 42% yield of alcohol after isolation and 40% yield of carbamate after isolation (conversion 43% calculated from GC, based on alcohol and carbonate)

Table S12. *E*-values for scope limitation reactions of DEKR, with **1p** serving as an acyl acceptor.

Carbonate (substrate)[a]	Reaction time (h)	C_1 (%)[b]	v_1 (mmol/h)	C_2 (%)[c]	v_2 (mmol/h)	*ee*S_1[d] (%)	*ee*S_2[e] (%)	*ee*P_1[f] (%)	*ee*P_2[g] (%)	Ec[h]	Ea[i]
**2a**	64	51	0.0021	51	0.0021	>99	95	71	92	30	93
**3a**	64	20	0.0008	21	0.0009	36	21	85	52	15	4
**2b**	64	29	0.0012	30	0.0012	28	42	65	98	6	150
**3b**	88	<1	0.0000	<1	0.0000	*nd*	*nd*	*nd*	*nd*	*nd*	*nd*
**2c**	88	27	0.0008	26	0.0008	43	30	69	99	7	>200
**2d**	64	41	0.0017	39	0.0016	15	65	22	98	2	195
**2e**	64	42	0.0017	40	0.0016	49	62	57	93	6	52

**2f**	64	47	0.0019	47	0.0019	87	84	88	97	44	183
**2g**	64	42	0.0017	41	0.0017	37	62	54	89	5	32
**2h**	64	46	0.0019	47	0.0019	92	85	>99	97	>200	183
**2i**	64	49	0.0020	49	0.0020	>99	92	>99	97	>200	>200
**2j**	64	36	0.0015	35	0.0014	55	51	>99	99	>200	112
**2k**	64	31	0.0013	32	0.0013	40	44	87	99	21	>200
**2l**	64	32	0.0013	30	0.0012	22	45	45	99	3	>200
**2m**	64	45	0.0018	46	0.0019	*nd*	83	*nd*	98	*nd*	>200
**2n**	64	45	0.0018	44	0.0018	-	78	-	99	-	>200

[a] According to the description in Figure 1. [b] GC conversion (C_1) and reaction velocity (v_1) calculated using carbonate and alcohol peaks,$v_1=C_1 \times n_{0S1} \times t^{-1}$ while t—reaction time, n_{0S1}—amount of carbonate added to the reaction vial. [c] GC conversion (C_2) and reaction velocity (v_2) calculated using amine and carbamate peaks, $v_2=C_2 \times n_{0S2} \times t^{-1}$ while t—reaction time, n_{0S2}—amount of carbonate added to the reaction vial. [d] Enantiomeric excess of (*S*)-carbonate. [e] Enantiomeric excess of (*S*)-amine (estimated using optical rotation measurements). [f] Enantiomeric excess of (*R*)-alcohol. [g] Enantiomeric excess of (*R*)-methyl (1-phenylethyl) carbamate. [h] E value calculated for reaction of R_3OOCO- group removal from alcohol. [i] E value calculated for amine acylation.

Enantiospecificity studies

To determine the enantiospecificity of the established method, studies on the racemization of the product and the reaction with different enantiomers of the substrate were performed. Under optimized conditions in a 3-mL vial, 1-phenylethylamine (1—racemic, 2—*R*-enantiomer, 3—*S*-enantiomer) (0.26 mmol) and 2a (0.26 mmol) were dissolved in 1 mL of toluene, enzyme was added (50 mg), and the reaction was placed on shaker (200 rpm, 50 °C). Conversions were measured by GC (sample preparation: 50 μL of reaction mixture dissolved in 1 mL of ethyl acetate) after 1, 2, 5, 10, and 20 days. When (*S*)-1-phenylethylamine was used as substrate, no conversion was observed. With (*R*)-1-phenylethylamine, conversion to product was observed and the reaction reached a final conversion of >99% (after 5 days); a racemate reaction was observed, as mentioned in the scope and limitation studies, and the final conversion was 50% (after 5 days). On the obtained product ((*R*)-carbamate >99 % *ee*), racemization was not observed. After 10 and 20 days of reaction, the alcohol and carbonate *ee* values were lower than obtained after 5 days.

Upscale studies:

Model reactions of 1a resulting in synthesis of 2a and 3a were performed using 11 mmol of racemic alcohol and 55 mmol of carbonate, 150 mg of enzyme, and 5 mL of MTBE. The proper amount of MTBE was important, for its presence decreased the conversion. At upscale, enantioselectivity was preserved and carbonates were obtained enantiospecifically (Table S13).

Table S13. Upscale studies for selected reactions.

Alcohol[a]	Acyl donor	conversion (%)	yield of alcohol (%)	yield of carbonate (%)	*ee*S1[b] (%)	*ee*P1[c] (%)	E
**1a**	DMC	29	67	26	42	>99	>200
**1a**	DEC	18	80	17	22	>99	>200
**1f**	DMC	38	58	35	53	>99	>200
**1h**	DMC	46	51	43	83	>99	>200
**1h**	DEC	44	52	43	79	>99	>200
**1i**	DMC	37	60	32	59	>99	>200
**1j**	DMC	44	53	40	72	>99	>200

[a] According to description in Figure.1. [b] Enantiomeric excess of alcohol. [c] Enantiomeric excess of carbonate.

Procedure for the upscaling of DEKR by aminolysis is provided in the main article in Section 3.5. Comprehensive results of the reaction optimization are gathered in Table S14

Table S14. Upscale studies for selected DEKR reactions engaging amine as acyl acceptor.

Carbonate[(a)]	Amine[(a)]	conversion (%)	yield of carbamate	yield of amine	yield of alcohol	yield of carbonate	*ee*S_1[b] (%)	*ee*S_2[c] (%)	*ee*P_1[d] (%)	*ee*P_2[e] (%)	E_1[f]	E_2[g]
**2a**	**1p**	45	44 %	48%	41%	55%	86	83	91	>99	59	>200
**2a**	**1r**	43	40%	51%	42%	54%	66	72	88	96	31	107
**2h**	**1p**	46	43%	41%	46%	50%	87	84	>99	>99	>200	>200
**2i**	**1p**	47	39%	40%	42%	53%	86	82	98	>99	>200	>200
**2j**	**1p**	44	36%	45%	38%	54%	83	77	99	>99	>200	>200

[a] According to description in Figure.1. [b] Enantiomeric excess of (*S*)-carbonate. [c] Enantiomeric excess of substrate (*S*)-amine (calculated after derivation mentioned in "Amine derivation" section). [d] Enantiomeric excess of (*R*)-alcohol. [e] Enantiomeric excess of (*R*)-methyl (1-phenylethyl) carbamate. [f] Enantioselectivity calculated using ee values of alcohol and carbonate. [g] Enantioselectivity calculated using ee values of amine and carbamate.

Spectral and analytical data

Alcohols:

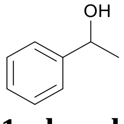

1-phenylethanol (1a): ^{1}H NMR (400 MHz, $CDCl_3$) δ 7.41–7.23 (m, 5H), 4.86 (q, J = 6.5 Hz, 1H), 2.34 (br s, 1H), 1.48 (d, J = 6.5 Hz, 3H); ^{13}C NMR (100 MHz, $CDCl_3$) δ 145.8, 128.4, 127.4, 125.4, 125.4, 70.3, 25.1. [44] Gas chromatography: t_r(*R*)= 19.33 min; t_r(*S*)= 19.61 min (according to general method 1).

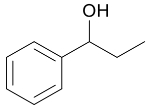

1-phenylpropanol (1b): ^{1}H NMR (400 MHz, $CDCl_3$) δ 7.44–7.23 (m, 5H), 4.54 (t, J = 6.6 Hz, 1H), 2.52 (br s, 1H), 1.96–1.63 (m, 2H), 0.91 (t, J = 7.5 Hz, 3H); ^{13}C NMR (100 MHz, $CDCl_3$) δ 144.6, 128.3, 127.4, 126.0, 75.9, 31.8, 10.1.[45] Gas chromatography: t_r(*R*)= 21.17 min ; t_r(*S*)= 21.31 min (according to general method 1). chiral HPLC: t_r(*R*)= 13.81 min ; t_r(*S*)= 15.56 min (hexane : isopropanol 97:3; OM T=15 °C). [46]

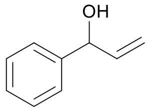

1-phenylprop-2-en-1-ol (1c): ^{1}H NMR (400 MHz, $CDCl_3$) δ 7.45–7.26 (m, 5H), 6.06 (ddd, J = 17.1, 10.5, 5.9 Hz, 1H), 5.35 (d, J = 17.1 Hz, 1H), 5.28–5.15 (m, 2H), 1.97 (br s, 1H); ^{13}C NMR (100 MHz, $CDCl_3$) δ 142.6, 140.2, 128.5, 127.7, 126.3, 115.1, 75.3. Spectroscopic data remained in agreement with literature.[47] Gas chromatography: t_r(*R*)= 21.36 min ; t_r(*S*)= 21.56 min; (according to general method 2). chiral HPLC: t_r(*R*)= 10.52 min ; t_r(*S*)= 16.61 min (hexane : isopropanol = 95:5; OM 15 °C).[48]

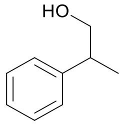

2-phenyl-1-propanol (1d): ^{1}H NMR (400 MHz, $CDCl_3$) δ 7.33 (dd, J = 8.0, 6.7 Hz, 2H), 7.30–7.15 (m, 3H), 3.70 (dd, J = 6.7, 1.2 Hz, 2H), 2.94 (p, J = 6.9 Hz, 1H), 1.57 (br s, 1H), 1.28 (d, J = 7.0 Hz, 3H); ^{13}C NMR (100 MHz, $CDCl_3$) δ 143.7, 128.6, 127.4, 126.6, 68.6, 42.4, 17.5.[49] Gas chromatography: t_r(*R*)= 31.00 min ; t_r(*S*)= 31.15 min (according to general method modified: temperature rate 3 °C/min (method 2)).

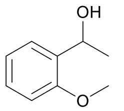

1-(2-methoxyphenyl)ethan-1-ol (1e): obtained according to general method for the ketone reduction from 2-methoxyacetophenone with yield: 97% (1.48 g; 9.7 mmol). Gas chromatography: t_r(*R*)= 24.29 min ; t_r(*S*)= 24.47 min (according to general method 1).[46] ^{1}H NMR (400 MHz, $CDCl_3$) δ 7.26 (t, J = 8.1 Hz, 1H), 6.99–6.90 (m, 2H), 6.81 (ddd, J = 8.2, 2.6, 1.1 Hz, 1H), 4.86 (q, J = 6.5 Hz, 1H), 3.81 (s, 3H), 1.92 (br s, 1H), 1.48 (d, J = 6.5 Hz, 3H); ^{13}C NMR (100 MHz, $CDCl_3$) δ 159.8, 147.6, 129.5, 117.6, 112.8, 110.9, 70.3, 55.2, 25.1. Spectroscopic data remained in agreement with literature.[50]

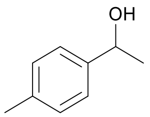

1-(4-methylphenyl)ethan-1-ol (1f): obtained according to general method for the ketone reduction from *p*-methylacetophenone with yield: 88% (1.3 g; 8.8 mmol). Gas chromatography: t_r(*R*)= 20.85 min ; t_r(*S*)= 21.20 min (according to general method 1).[46] ^{1}H NMR (400 MHz, $CDCl_3$) δ 7.31 (d, *J* = 8.2 Hz, 2H), 7.22 (d, *J* = 8.1 Hz, 2H), 4.84 (q, *J* = 6.5 Hz, 1H), 3.42 (br s, 1H), 2.44 (s, 3H), 1.52 (d, *J* = 6.6 Hz, 3H); ^{13}C NMR (100 MHz, $CDCl_3$) δ 143.2, 136.8, 129.1, 125.5, 69.9, 25.2, 21.1. Spectroscopic data remained in agreement with literature.[50]

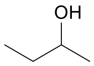

sec-butanol (1g):^{1}H NMR (400 MHz, $CDCl_3$) δ 3.65 (h, *J* = 6.2 Hz, 1H), 2.17 (br s, 1H), 1.41 (td, *J* = 14.3, 7.6 Hz, 2H), 1.11 (d, *J* = 6.2 Hz, 3H), 0.86 (t, *J* = 7.5 Hz, 3H).; ^{13}C NMR (100 MHz, $CDCl_3$) δ 69.2, 31.9, 22.7, 9.8. Gas chromatography: t_r(*S*)= 6.26 min ; t_r(*R*)= 6.43 min (according to general method 1). [51]

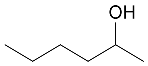

hexan-2-ol (1h): Gas chromatography: t_r(*R*)= 10.79 min ; t_r(*S*)= 10.89 min (according to general method 1). Optical rotation: $[\alpha]_D^{25}$ -5.5 (c 0.23, $CHCl_3$) 98 % *ee* (lit. $[\alpha]_D^{25}$ -5.4 (c 0.23, $CHCl_3$) 97.7 % *ee*). [52] ^{1}H NMR (400 MHz, $CDCl_3$) δ 3.81–3.66 (m, 1H), 2.03 (br s, 1H), 1.52–1.20 (m, 6H), 1.13 (d, *J* = 6.3 Hz, 3H), 0.86 (t, *J* = 7.1 Hz, 3H); ^{13}C NMR (100 MHz, $CDCl_3$) δ 68.00, 38.98, 27.90, 23.32, 22.65, 13.96. Spectroscopic data remained in agreement with literature. [53]

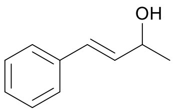

4-phenylbut-3-en-2-ol (1i): obtained according to general method for the ketone reduction from 4-phenylbut-3-en-2-one with yield: 98% (1.45 g; 9.8 mmol). Gas chromatography: t_r(*R*)= 24.38 min ; t_r(*S*)= 24.44 min (according to general method 1). Optical rotation: $[\alpha]_D^{25}$ +27.8 (1.57, $CHCl_3$) 98 % *ee* (lit. $[\alpha]_D^{25}$ +27.5 (1.59, $CHCl_3$) 95.3 % *ee*) [44] ^{1}H NMR (400 MHz, $CDCl_3$) δ 7.43–7.35 (m, 2H), 7.35–7.27 (m, 2H), 7.27–7.20 (m, 1H), 6.56 (dd, *J* = 15.9, 1.3 Hz, 1H), 6.26 (dd, *J* = 15.9, 6.3 Hz, 1H), 4.48 (pd, *J* = 6.4, 1.3 Hz, 1H), 2.19 (br s, 1H), 1.38 (d, *J* = 6.3 Hz, 3H); ^{13}C NMR (100 MHz, $CDCl_3$) δ 136.7, 133.7, 129.3, 128.5, 127.6, 126.4, 68.8, 23.4. Spectroscopic data remained in agreement with literature. [54]

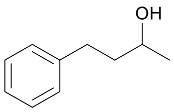

4-phenylbutan-2-ol (1j): obtained in two-step reduction with yield: 72% (1.08 g; 7.2 mmol). Gas chromatography: t_r(*S*)= 22.64 min ; t_r(*R*)= 22.74 min (according to general method 1). [45] ^{1}H NMR (400 MHz, $CDCl_3$) δ 7.35–7.26 (m, 2H), 7.26–7.17 (m, 3H), 3.84 (dqd, *J* = 7.5, 6.2, 5.1 Hz, 1H), 2.85–2.63 (m, 2H), 2.31 (br s, 1H), 1.89–1.71 (m, 2H), 1.25 (d, *J* = 6.2 Hz, 3H); ^{13}C NMR (100 MHz, $CDCl_3$) δ 142.2, 128.4, 128.4, 125.8, 67.3, 40.8, 32.1, 23.5. Spectroscopic data remained in agreement with literature. [55]

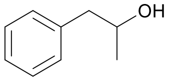

1-phenylprop-2-en-1-ol (1k): Gas chromatography: t_r(*S*)= 19.72 min ; t_r(*R*)= 19.82 min (according to general method 1). [56] chiral HPLC: t_r(*R*)= 7.27 min ; t_r(*S*)= 7.91 min (hexane : isopropanol = 94:6; OM 15 °C) ^{1}H NMR (400 MHz, $CDCl_3$) δ 7.37–7.27 (m, 2H), 7.27–7.18 (m, 3H), 4.09–3.95 (m, 1H), 2.84–2.62 (m, 2H), 1.24 (d, *J* = 6.2 Hz, 3H); ^{13}C NMR (101 MHz, $CDCl_3$) δ 138.57, 129.41, 126.47, 126.47, 68.86, 45.81, 22.79. Spectroscopic data remained in agreement with literature. [57]

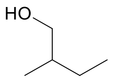

2-methyl-butan-1-ol (1l): Gas chromatography: t_r= 13.2 min (according to general method 2). Optical rotation: (*S*) enantiomer $[\alpha]_D^{25}$ −31.2 (c 0.85, $CHCl_3$) >99 % *ee* was obtained for commercial optically pure alcohol. ^{1}H NMR (400 MHz, $CDCl_3$) δ 3.48–3.22 (m, 2H), 2.88 (br s, 1H), 1.41 (dddd, *J* = 27.5, 13.0, 7.2, 5.6 Hz, 2H), 1.14–0.98 (m, 1H), 0.83 (dt, *J* = 7.3, 4.0 Hz, 6H); ^{13}C NMR (100 MHz, $CDCl_3$) δ 67.6, 37.2, 25.7, 16.0, 11.1. Spectroscopic data remained in agreement with literature. [58]

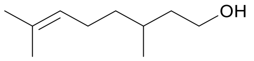

β-citronellol (1m): Gas chromatography: t_r= 20.87 min (according to general method 1). Optical rotation measurements suggested that citronellol obtained in DEKR was racemic. ^{1}H NMR (400 MHz, $CDCl_3$) δ 5.17–5.01 (m, 1H), 3.74–3.55 (m, 2H), 2.10–1.88 (m, 2H), 1.74–1.46 (m, 10H), 1.42–1.24 (m, 2H), 1.16 (dddd, *J* = 13.4, 9.4, 7.6, 6.1 Hz, 1H), 0.89 (d, *J* = 6.3 Hz, 3H); ^{13}C NMR (100 MHz, $CDCl_3$) δ 131.1, 124.7, 61.0, 39.8, 37.2, 29.1, 25.6, 25.4, 19.4, 17.5. [59]

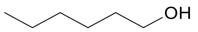

***n*-hexanol (1n)**: Gas chromatography: t_r= 12.84 min (according to general method 1). ^{1}H NMR (400 MHz, $CDCl_3$) δ 3.57 (t, *J* = 6.7 Hz, 2H), 2.34 (dd, *J* = 3.9, 1.9 Hz, 1H), 1.57–1.44 (m, 2H), 1.37–1.19 (m, 6H), 0.91–0.79 (m, 3H).; ^{13}C NMR (100 MHz, $CDCl_3$) δ 62.7, 32.6, 31.6, 25.3, 22.5, 13.9. [60]

Carbonates:

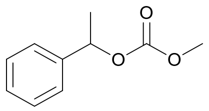

methyl 1-phenylethyl carbonate (2a): obtained according to general method with yield: 95% (1.71 g; 9.5 mmol). Gas chromatography: t_r(*S*)= 20.01 min ; t_r(*R*)= 20.1 min (according to general method 1). 1H NMR (400 MHz, CDCl3) δ 7.41–7.28 (m, 5H), 5.74 (q, J = 6.6 Hz, 1H), 3.75 (s, 3H), 1.60 (d, J = 6.6 Hz, 3H); 13C NMR (100 MHz, CDCl3) δ 155.1, 141.0, 128.5, 128.1, 126.0, 76.4, 54.5, 22.2. Spectroscopic data remained in agreement with literature. [61]

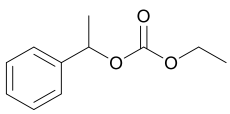

ethyl 1-phenylethyl carbonate (3a): obtained according to general method with yield: 96% (1.86 g; 9.6 mmol). Gas chromatography: t_r= 21.58 min ; (according to general method 1). chiral HPLC: t_r(*S*)= 6.00 min ; t_r(*R*)= 6.44 min (hexane:isopropanol : 98:2; column: OM; T=15 °C) ^{1}H NMR (400 MHz, $CDCl_3$) δ 7.41–7.23 (m, 5H), 5.73 (q, *J* = 6.6 Hz, 1H), 4.15 (q, *J* = 7.1 Hz, 2H), 1.57 (d, *J* = 6.7 Hz, 3H), 1.25 (t, *J* = 7.1 Hz, 3H); ^{13}C NMR (100 MHz, $CDCl_3$) δ 154.5, 141.2,

128.4, 127.9, 125.9, 76.0, 63.6, 26.8, 22.2. Spectroscopic data remained in agreement with literature. [62]

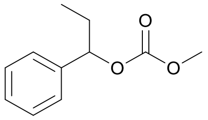

methyl 1-phenylpropyl carbonate (2b): obtained according to general method with yield: 94% (1.82 g; 9.4 mmol). Gas chromatography: t_r(*S*)= 21.33 min ; t_r(*R*)= 21.41 min (according to general method 1). chiral HPLC: t_r(*S*)= 5.88 min ; t_r(*R*)= 6.48 min (hexane : isopropanol = 98:2, OM column, 15 °C) ^{1}H NMR (400 MHz, $CDCl_3$) δ 7.42–7.28 (m, 5H), 5.57 (q, *J* = 7.2, 6.3 Hz, 1H), 3.74 (s, 3H), 1.97–1.74 (m, 2H), 0.93 (t, *J* = 7.2 Hz, 3H); ^{13}C NMR (100 MHz, $CDCl_3$) δ 155.3, 140.0, 128.4, 128.4, 128.0, 126.4, 81.5, 54.4, 29.3, 9.7. HRMS (EI) calcd. for $C_{11}H_{14}O_3$ $[M+H]^+$ 194.0943 , found 194.0944.

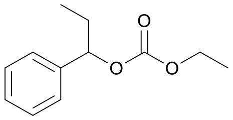

ethyl 1-phenylpropyl carbonate (3b): obtained according to general method with yield: 96% (1.99 g; 9.6 mmol). Gas chromatography: t_r= 24.57 min ; (according to general method 1). chiral HPLC: t_r(*S*)= 6.11 min ; t_r(*R*)= 6.60 min (hexane : isopropanol : 98:2; column: OM; T=15 °C) ^{1}H NMR (400 MHz, $CDCl_3$) δ 7.54–7.12 (m, 5H), 5.61–5.37 (m, 1H), 4.17 (q, *J* = 7.3, 2H), 2.10–1.76 (m, 2H), 1.28 (t, *J* = 7.3 Hz, 3H), 0.92 (t, *J* = 7.3, 3H); ^{13}C NMR (100 MHz, $CDCl_3$) δ 154.7, 140.0, 128.4, 128.4, 128.4, 128.0, 128.0, 126.5, 126.5, 81.3, 63.8, 29.3, 14.2, 9.8. (EI) calcd. for $C_{12}H_{16}O_3$ $[M+H]^+$ 208.1099 , found 208.1100.

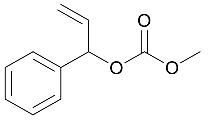

methyl 1-phenylprop-2-en-1-yl carbonate (2c): obtained according to general method with yield: 95% (1.83 g; 9.5 mmol). Gas chromatography: tr= 21.5 min ; (according to general method 1). 1H NMR (400 MHz, CDCl3) δ 7.38–7.29 (m, 5H), 6.13–5.97 (m, 2H), 5.40–5.25 (m, 2H), 3.78 (s, 3H); 13C NMR (100 MHz, CDCl3) δ 155.0, 138.2, 135.7, 128.6, 128.5, 128.4, 127.0, 126.3, 117.4, 80.1, 54.7. chiral HPLC: t_r(*R*)= 6.43 min ; t_r(*S*)= 7.55 min (hexane : isopropanol = 98:2; 15 °C) Obtained data remained in agreement with those presented in literature. [63]

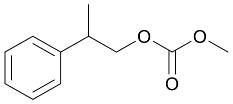

methyl 2-phenyl-1-propyl carbonate (2d): obtained according to general method with yield: 98% (1.90 g; 9.8 mmol). Gas chromatography: t_r= 33.76 min ; (according to general method 2). Optical rotation: $[\alpha]_D^{25}$=-0.67 (c 0.69, $CHCl_3$) 23 % optical purity (*R*)- methyl 2-phenyl-1-ethyl carbonate $[\alpha]_D^{25}$=-2.87 (c 0.68, $CHCl_3$) >99 % *ee*–obtained for standard) ^{1}H NMR (400 MHz, $CDCl_3$) δ 7.38–7.30 (m, 2H), 7.30–7.21 (m, 3H), 4.35–4.14 (m, 2H), 3.75 (s, 3H), 3.16 (q, *J* = 7.0 Hz, 1H), 1.35 (d, *J* = 7.1 Hz, 3H); ^{13}C NMR (100 MHz, $CDCl_3$) δ 155.7, 142.7, 128.5, 127.3, 126.8, 72.8, 54.6, 39.0, 17.8. HRMS (ESI) calcd. for $C_{11}H_{14}O_3$ $[M+H]^+$ 217.0841 , found 217.0844.

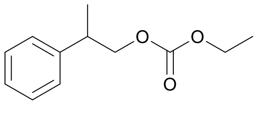

ethyl 2-phenyl-1-propyl carbonate (3d): obtained according to general method with yield: 96% (1.99 g; 9.6 mmol). Gas chromatography: t_r= 36.20 min ; (according to general method 2). ^{1}H NMR (400 MHz, $CDCl_3$) δ 7.37–7.29 (m, 2H), 7.29–7.21 (m, 3H), 4.29 (dd, *J* = 10.5, 6.6 Hz, 1H), 4.22–4.13 (m, 3H), 3.15 (p, *J* = 7.0 Hz, 1H), 1.35 (d, *J* = 7.0 Hz, 3H), 1.28 (t, *J* = 7.1 Hz, 3H); ^{13}C NMR (100 MHz, $CDCl_3$) δ 155.1, 142.7, 128.5, 127.2, 126.7, 72.5, 63.8, 39.0, 17.8, 14.2. HRMS (ESI) calcd. for $C_{12}H_{16}O_3$ $[M+H]^+$ 231.0997 , found 231.1003.

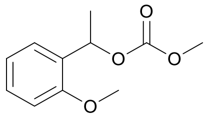

methyl 1-(2-methoxyphenyl)ethan-1-yl carbonate (2e): obtained according to general method with yield: 92% (1.93 g; 9.2 mmol). Gas chromatography: t_r(*S*)= 25.33 min ; t_r(*R*)= 25.38 min (according to general method 1). ^{1}H NMR (400 MHz, $CDCl_3$) δ 7.26 (t, *J* = 7.9 Hz, 1H), 6.98–6.89 (m, 2H), 6.83 (ddd, *J* = 8.2, 2.6, 1.0 Hz, 1H), 5.70 (q, *J* = 6.6 Hz, 1H), 3.79 (s, 3H), 3.75 (s, 3H), 1.58 (d, *J* = 6.6 Hz, 3H); ^{13}C NMR (100 MHz, $CDCl_3$) δ 159.7, 155.1, 142.7, 129.6, 118.2, 113.5, 111.5, 76.2, 55.1, 54.5, 22.3. HRMS (EI) calcd. for $C_{11}H_{14}O_3$ $[M+H]^+$ 210.0892 , found 210.0896.

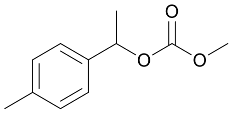

methyl 1-(4-methylphenyl)ethan-1-yl carbonate (2f): obtained according to general method with yield: 98% (1.90 g; 9.8 mmol). Gas chromatography: t_r(*S*)= 22.48 min ; t_r(*R*)= 22.56 min (according to general method 1). ^{1}H NMR (400 MHz, $CDCl_3$) δ 7.29 (d, *J* = 8.2 Hz, 2H), 7.18 (d, *J* = 7.9 Hz, 2H), 5.72 (q, *J* = 6.6 Hz, 1H), 3.75 (s, 3H), 2.36 (s, 3H), 1.60 (d, *J* = 6.6 Hz, 3H); ^{13}C NMR (100 MHz, $CDCl_3$) δ 155.1, 138.1, 137.8, 129.2, 129.1, 126.0, 76.3, 54.4, 22.2, 21.0. HRMS (EI) calcd. for $C_{11}H_{14}O_3$ $[M+H]^+$ 194.0943 , found 194.0941.

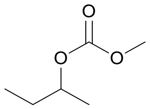

butan-2-yl methyl carbonate (2g): obtained according to general method with yield: 94% (1.24 g; 9.4 mmol). Gas chromatography: t_r(*S*)= 8.59 min ; t_r(*R*)= 8.75 min (according to general method 1). ^{1}H NMR (400 MHz, $CDCl_3$) δ 4.70–4.60 (m, 1H), 3.69 (s, 3H), 1.64–1.52 (m, 2H), 1.20 (d, J = 6.3 Hz, 3H), 0.87 (t, *J* = 7.5 Hz, 3H); ^{13}C NMR (100 MHz, $CDCl_3$) δ 155.4, 76.3, 54.0, 31.5, 19.1, 9.3. Spectroscopic data remained in agreement with literature. [64]

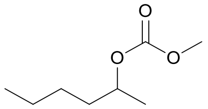

hexan-2-yl methyl carbonate (2h): obtained according to general method with yield: 97% (1.55 g; 9.7 mmol). Gas chromatography: t_r(*S*)= 13.00 min ; t_r(*R*)= 13.23 min (according to general method 1). Optical rotation: $[\alpha]_D^{25}$ +14.72 (c 0.66 , $CHCl_3$) 87 % *ee*. ^{1}H NMR (400 MHz, $CDCl_3$) δ 4.69 (dt, *J* = 6.6, 0.9 Hz, 1H), 3.69 (s, 3H), 1.58 (ddd, *J* = 12.3, 7.6, 4.6 Hz, 1H), 1.49–1.42 (m, 1H), 1.23 (dd, *J* = 19.2, 5.1 Hz, 7H), 0.83 (t, *J* = 7.2 Hz, 3H); ^{13}C NMR (100 MHz, $CDCl_3$) δ 155.4, 75.2, 54.1, 34.5, 27.3, 22.5, 19.7, 13.7. Spectroscopic data remained in agreement with literature. [65]

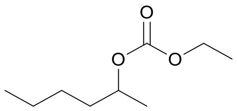

hexan-2-yl ethyl carbonate (3h): obtained according to general method with yield: 98% (1.71 g; 9.8 mmol). Gas chromatography: t_r(*S*)= 14.49 min ; t_r(*R*)= 14.59 min (according to general method 1). ^{1}H NMR (400 MHz, $CDCl_3$) δ 4.78–4.67 (m, 1H), 4.20–4.10 (m, 2H), 1.62 (ddt, *J* = 10.4, 5.4, 1.7 Hz, 1H), 1.49 (dddd, *J* = 11.5, 7.7, 5.8, 3.9 Hz, 1H), 1.32–1.22 (m, 13H); ^{13}C NMR (100 MHz, $CDCl_3$) δ 154.8, 75.1, 63.4, 35.5, 27.4, 22.4, 19.8, 14.1, 13.8. [65,66]

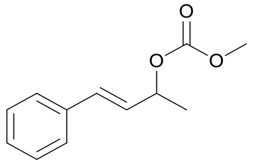

methyl 4-phenylbut-3-en-2-yl carbonate (2i): obtained according to general method with yield: 96% (1.98 g; 9.6 mmol). Gas chromatography: t_r(*S*)= 26.77 min ; t_r(*R*)= 26.84 min (according to general method 1). ^{1}H NMR (400 MHz, $CDCl_3$) δ 7.43–7.35 (m, 2H), 7.35–7.29 (m, 2H), 7.28–7.21 (m, 1H), 6.66 (d, *J* = 16.0 Hz, 1H), 6.20 (dd, *J* = 16.0, 6.9 Hz, 1H), 5.44–5.34 (m, 1H), 3.78 (s, 3H), 1.47 (d, *J* = 6.5 Hz, 3H); ^{13}C NMR (100 MHz, $CDCl_3$) δ 155.1, 132.2, 128.5, 128.0, 126.6, 75.2, 54.5, 20.4. Spectroscopic data remained in agreement with literature. [61]

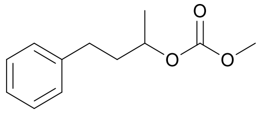

methyl 4-phenylbutan-2-yl carbonate (2j): obtained according to general method with yield: 97% (2.02 g; 9.7 mmol). Gas chromatography: t_r(*R*)= 25.04 min ; t_r(*S*)= 25.10 min (according to general method 1). ^{1}H NMR (400 MHz, $CDCl_3$) δ 7.29 (dd, *J* = 8.5, 6.5 Hz, 2H), 7.23–7.10 (m, 3H), 4.89–4.77 (m, 1H), 3.79 (s, 3H), 2.70 (qdd, *J* = 13.8, 9.9, 6.1 Hz, 2H), 2.01 (dddd, *J* = 13.5, 9.7, 7.7, 5.8 Hz, 1H), 1.92–1.79 (m, 1H), 1.33 (d, *J* = 6.1 Hz, 3H); ^{13}C NMR (100 MHz, $CDCl_3$) δ 155.4, 141.3, 128.4, 128.3, 125.9, 74.7, 54.4, 37.6, 31.6, 19.9. HRMS (ESI) calcd. for $C_{12}H_{16}O_3$ $[M+H]^+$ 231.0997 , found 231.1000.

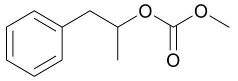

methyl 1-phenylpropan-2-yl carbonate (2k): obtained according to general method with yield: 97% (1.88 g; 9.7 mmol). Gas chromatography: t_r= 22.29 min (according to general method 1). chiral HPLC: t_r(*S*)= 6.92 min ; t_r(*R*)= 8.93 min (hexane : isopropanol = 98:2; 15 °C) ^{1}H NMR (400 MHz, $CDCl_3$) δ 7.37–7.13 (m, 5H), 4.97 (q, *J* = 6.4 Hz, 1H), 3.74 (s, 3H), 2.90 (ddd, *J* = 90.8, 13.6, 6.6 Hz, 2H), 1.28 (d, *J* = 6.3 Hz, 3H); ^{13}C NMR (101 MHz, $CDCl_3$) δ 155.26, 137.19, 129.43, 128.41, 126.58, 75.76, 54.46, 42.25, 19.36. HRMS (ESI) calcd. for $C_{11}H_{14}O_3$ $[M+Na]^+$ 217.0841, found 217.0844.

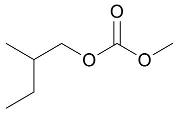

methyl 2-methyl-butan-1-yl carbonate (2l): obtained according to general method with yield: 89% (1.30 g; 8.9 mmol). Gas chromatography: t_r= 15.26 min ; (according to general method 2). Optical rotation for pure *S*-enantiomer: $[\alpha]_D^{25}$=+9.14 (c 1.03, $CHCl_3$) >99 % *ee*. ^{1}H NMR (400 MHz, $CDCl_3$) δ 4.04–3.86 (m, 2H), 3.73 (s, 3H), 1.74–1.65 (m, 1H), 1.42 (ddd, *J* = 13.3, 7.5, 5.7 Hz, 1H), 1.27 (d, *J* = 8.4 Hz, 1H), 1.20–1.11 (m, 1H), 0.83-0.92 (m, 5H); ^{13}C NMR (100 MHz, $CDCl_3$) δ 155.9, 72.6, 54.5, 22.6, 16.1, 14.0. elemental analysis calc. for $C_7H_{14}O_3$: C 57.51 %; H 9.61 %; O 32.83 %, found: C 57.23 %; H 9.61 % O 33.16 %.

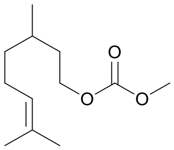

β-citronellyl methyl carbonate (2m): obtained according to general method with yield: 97% (2.07 g; 9.7 mmol). Gas chromatography: t_r= 23.33 min ; (according to general method 1). Optical rotation measurements showed that compound 2m was not obtained stereoselectively ^{1}H NMR (400 MHz, $CDCl_3$) δ 5.11–4.95 (m, 1H), 4.22–3.99 (m, 2H), 3.68 (d, *J* = 10.6 Hz, 3H), 1.91 (q, *J* = 8.7 Hz, 2H), 1.68–1.56 (m, 4H), 1.52 (d, *J* = 8.9 Hz, 4H), 1.44–1.34 (m, 1H), 1.34–1.22 (m, 1H), 1.19–1.05 (m, 1H), 0.91–0.77 (m, 3H); ^{13}C NMR (100 MHz, $CDCl_3$) δ 155.7, 131.1, 124.4, 66.4, 54.3, 36.8, 35.4, 29.1, 25.2, 19.1, 17.4. Spectroscopic data remained in agreement with literature. [67]

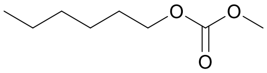

***n*-hexyl methyl carbonate (2n)**: obtained according to general method with yield: 98% (1.57 g; 9.8 mmol). Gas chromatography: t_r= 15.56 min. (according to general method 1). ^{1}H NMR (400 MHz, $CDCl_3$) δ 3.99 (t, *J* = 6.7 Hz, 2H), 3.62 (s, 3H), 1.57–1.49 (m, 2H), 1.27–1.16 (m, 6H), 0.78 (t, J = 7.0 Hz, 3H); ^{13}C NMR (100 MHz, $CDCl_3$) δ 155.6, 67.8, 54.0, 31.2, 28.5, 25.2, 22.3, 13.6. Spectroscopic data remained in agreement with literature. [68,69]

Carbamates:

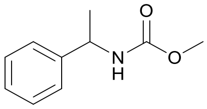

methyl (1-phenylethyl)carbamate (2o): obtained according to general method with yield: 98% (0.88 g; 4.9 mmol). Gas chromatography: tr= 24.89 min ; (according to general method 1). chiral HPLC: t_r(*S*)= 14.44 min ; t_r(*R*)= 26.43 min (hexane : isopropanol 94:6 on OM column) Optical rotation: $[\alpha]_D^{25}$ +156.8 (c 0.35, $CHCl_3$) >99 % *ee*. ^{1}H NMR (400 MHz, $CDCl_3$) δ 7.40–7.19 (m, 5H), 5.26 (s, 1H), 4.84 (s, 1H), 3.64 (s, 3H), 1.46 (d, *J* = 7.0 Hz, 3H); ^{13}C NMR (100 MHz, $CDCl_3$) δ 156.3, 143.7, 128.5, 127.2, 125.9, 52.0, 50.6, 22.4. Spectroscopic data remained in agreement with literature. [70,71]

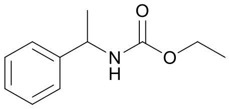

ethyl (1-phenylethyl)carbamate (3o): obtained according to general method with yield: 97% (0.94 g; 4.85 mmol). Gas chromatography: t_r(1)= 25.89 min ; t_r(2)= 25.93 min (according to general method 1). chiral HPLC: t_r(*S*)= 10.74 min ; t_r(*R*)= 14.08 min (hexane : isopropanol = 94:6; column OM) ^{1}H NMR (400 MHz, $CDCl_3$) δ 7.36–7.20 (m, 5H), 5.20 (s, 1H), 4.84 (s, 1H), 4.09 (tq, *J* = 7.1, 3.4 Hz, 2H), 1.46 (d, *J* = 7.0 Hz, 3H), 1.20 (t, *J* = 7.2 Hz, 3H); ^{13}C NMR (100 MHz, $CDCl_3$) δ 155.9, 143.8, 128.5, 127.1, 125.9, 60.7, 50.5, 22.4, 14.5. Spectroscopic data remained in agreement with literature. [72]

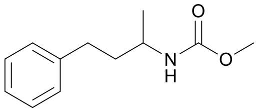

methyl (4-phenylbutan-2-yl)carbamate (2p): obtained according to general method with yield: 89% (0.92 g; 4.45 mmol). Gas chromatography: t_r= 28.82 min ; (according to general

method 1). chiral HPLC: t_r(*S*)= 20.72 min ; t_r(*R*)= 20.83 min (hexane : isopropanol = 95:5; OM column, T=15°C) ^{1}H NMR (400 MHz, $CDCl_3$) δ 7.32–7.24 (m, 2H), 7.18 (ddd, *J* = 5.5, 3.7, 2.2 Hz, 3H), 4.56 (s, 1H), 3.75 (s, 1H), 3.67 (s, 3H), 2.66 (td, *J* = 7.7, 4.4 Hz, 2H), 1.83–1.64 (m, 2H), 1.17 (d, *J* = 6.6 Hz, 3H); ^{13}C NMR (100 MHz, $CDCl_3$) δ 141.7, 128.4, 128.4, 128.3, 125.8, 51.8, 46.9, 38.9, 32.4, 21.3. HRMS (ESI) calcd. for $C_{12}H_{17}NO_2$ $[M+H]^+$ 230.1157 , found 230.1157.

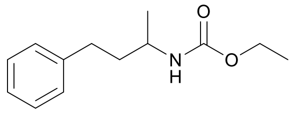

ethyl (4-phenylbutan-2-yl)carbamate (3p): obtained according to general method with yield: 71% (0.78 g; 3.55 mmol). Gas chromatography: t_r = 30.08 min (according to general method 1) ^{1}H NMR (400 MHz, $CDCl_3$) δ 7.27 (dd, *J* = 8.2, 6.9 Hz, 2H), 7.22–7.13 (m, 3H), 4.69 (s, 1H), 4.12 (q, *J* = 7.2 Hz, 2H), 3.76 (s, 1H), 2.66 (td, *J* = 7.5, 4.7 Hz, 2H), 1.74 (qd, *J* = 7.0, 6.5, 4.4 Hz, 2H), 1.24 (t, *J* = 7.1 Hz, 3H), 1.17 (d, *J* = 6.6 Hz, 3H); ^{13}C NMR (100 MHz, $CDCl_3$) δ 156.1, 141.8, 128.3, 128.3, 125.8, 60.4, 46.8, 38.9, 32.4, 21.3, 14.6. HRMS (ESI) calcd. for $C_{13}H_{19}NO_2$ $[M+H]^+$ 244.1313 , found 244.1319. To determine enantiomeric excess carbamate group was removed and amine was derived by acylation, and enantiomeric excesses were determined by chiral GC.

Amines:

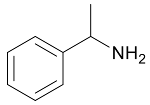

1-phenylethylamine (1o): Gas chromatography: t_r(*R*)= 16.33 min ; t_r(*S*)= 16.46 min ; (according to general method). Optical rotation: $[\alpha]_D^{25}$ -65 (c 0.82, $CHCl_3$) 81 % *ee*. Chromatographic data after amine derivation: Gas chromatography: t_r(*S*)= 25.6 min ; t_r(*R*)= 25.8 min (according to general method 1) ^{1}H NMR (400 MHz, $CDCl_3$) δ 7.42–7.28 (m, 4H), 7.27–7.18 (m, 1H), 4.10 (q, *J* = 6.6 Hz, 1H), 1.59 (s, 2H), 1.38 (d, *J* = 6.7 Hz, 3H); ^{13}C NMR (100 MHz, $CDCl_3$) δ 147.8, 128.4, 128.4, 126.7, 125.6, 125.6, 51.3, 25.6.: Spectroscopic data remained in agreement with literature. [73,74]

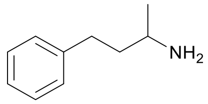

4-phenylbutan-2-amine (1p): Gas chromatography: t_r= 22.43 min ; (according to general method 1). Optical rotation: $[\alpha]_D^{25}$ + 3.78 (c 0.97, $CHCl_3$) 52 % *ee* (literature: $[\alpha]_D^{20}$ +7.1 (c 1.0, $CHCl_3$) >99 % *ee*)[37] ^{1}H NMR (400 MHz, $CDCl_3$) δ 7.33–7.21 (m, 2H), 7.21–7.11 (m, 3H), 2.89 (h, *J* = 6.3 Hz, 1H), 2.64 (qdd, *J* = 13.7, 9.4, 6.6 Hz, 2H), 1.71–1.54 (m, 2H), 1.08 (d, *J* = 6.3 Hz, 5H); ^{13}C NMR (100 MHz, $CDCl_3$) δ 142.3, 128.3, 128.3, 125.7, 46.5, 41.8, 32.8, 24.1. Spectroscopic data remained in agreement with literature. [75,76] Chromatographic data after amine derivation: Gas chromatography: t_r(*S*)= 29.64 min ; t_r(*R*)= 29.78 min (according to general method 1).

NMR spectra, HPLC and GC data:

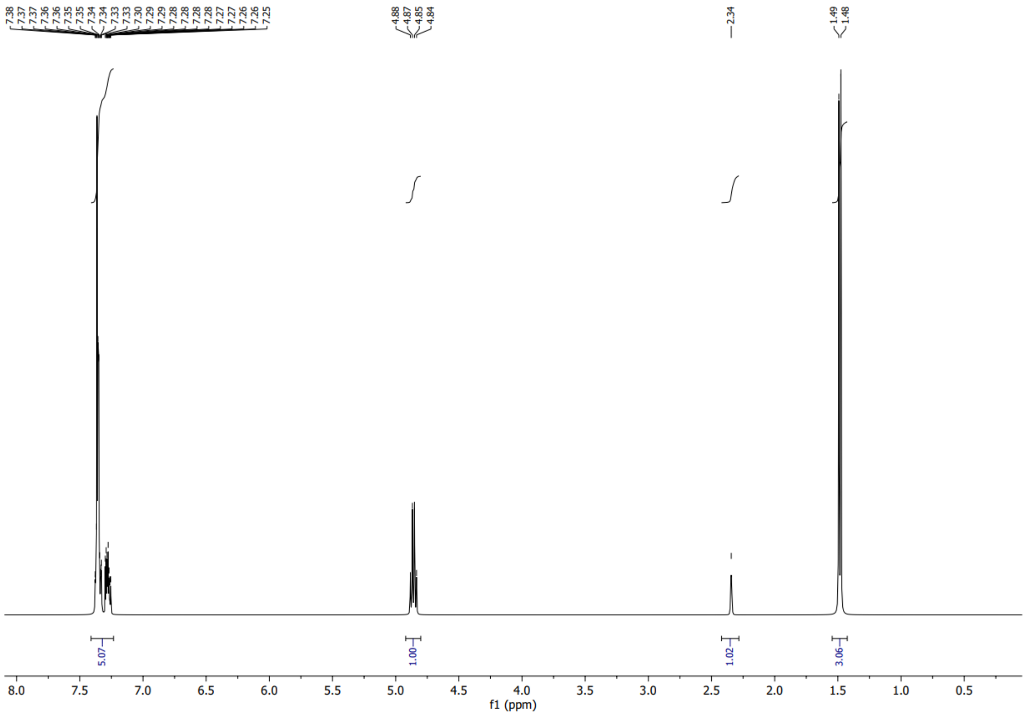

^{1}HNMR (400 MHz; $CDCl_3$) spectrum of compound **1a**.

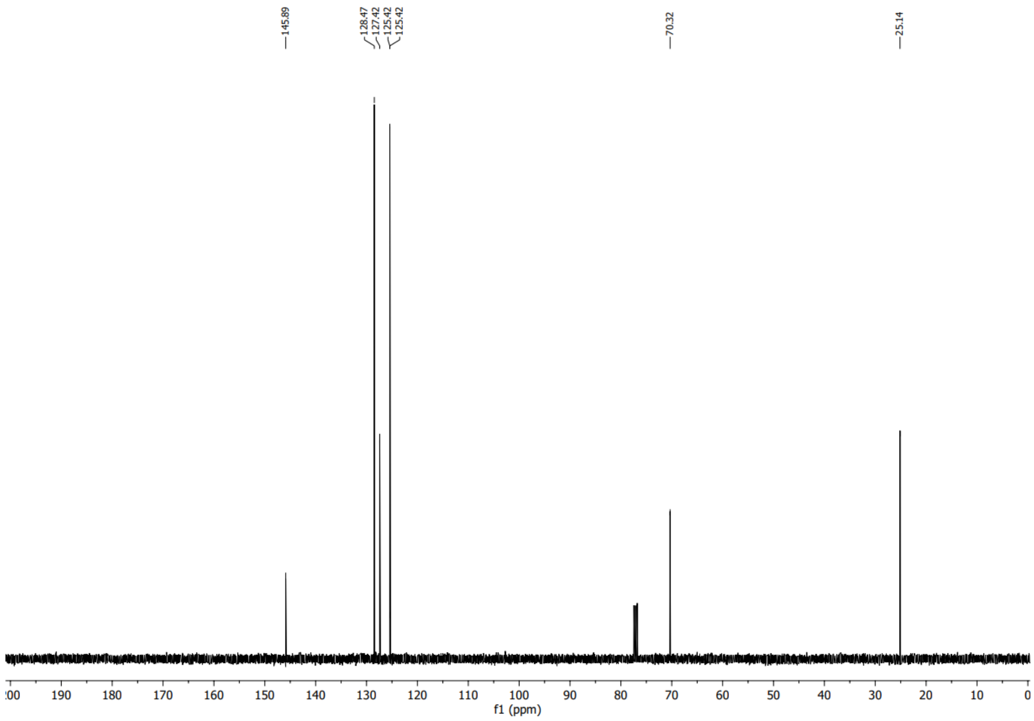

^{13}CNMR (100 MHz; $CDCl_3$) spectrum of compound **1a**

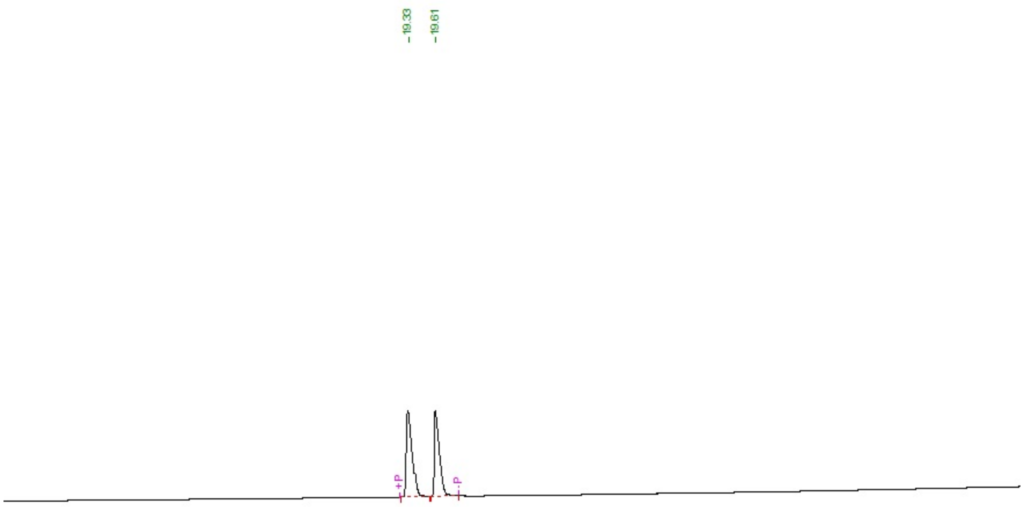

GC chromatogram (general GC method 1) of racemic compound **1a**.

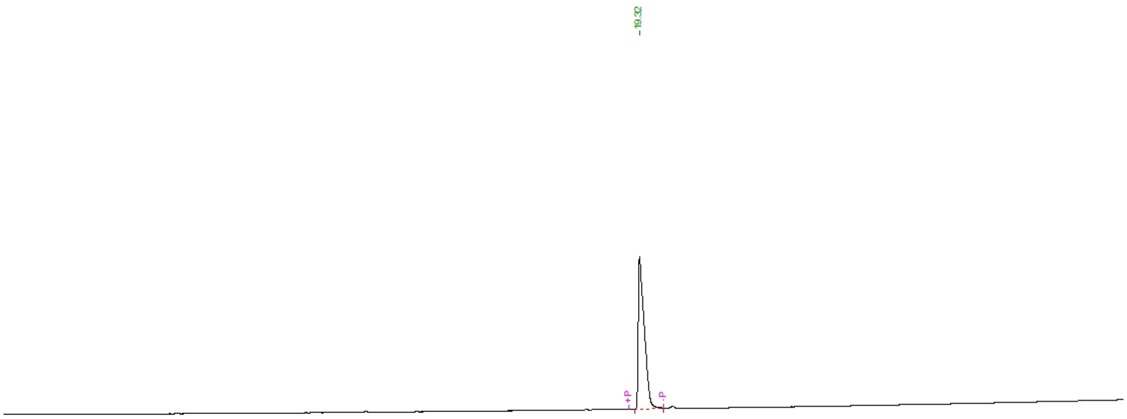

GC chromatogram (general GC method 1) of (*R*)-enantiomer compound **1a**.

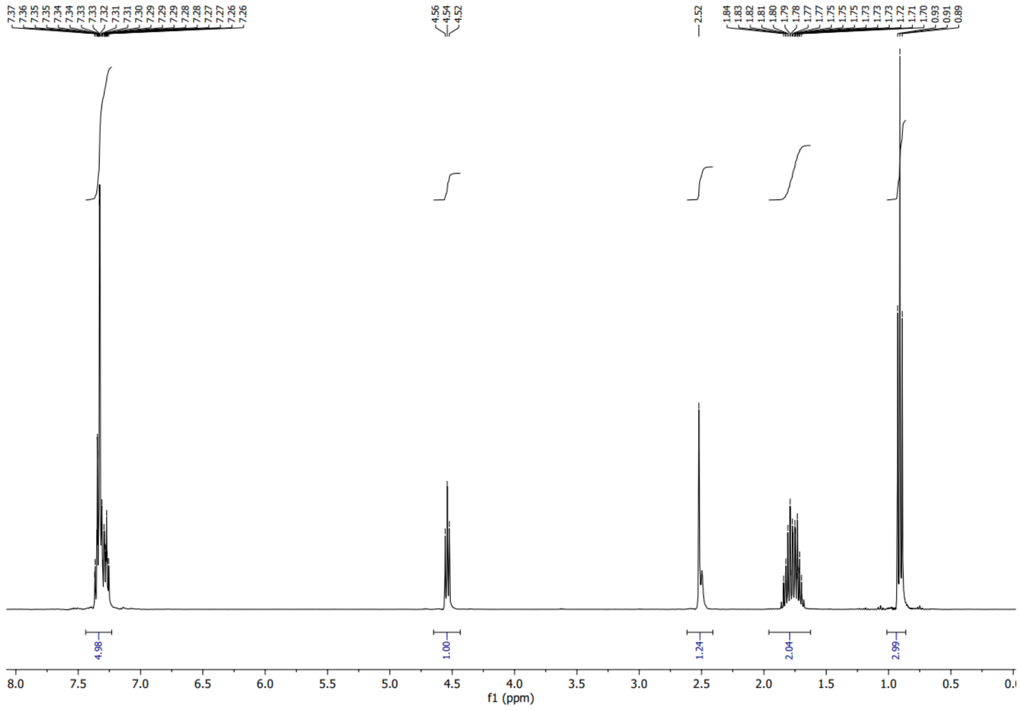

^{1}HNMR (400 MHz; CDCl3) spectrum of compound **1b**.

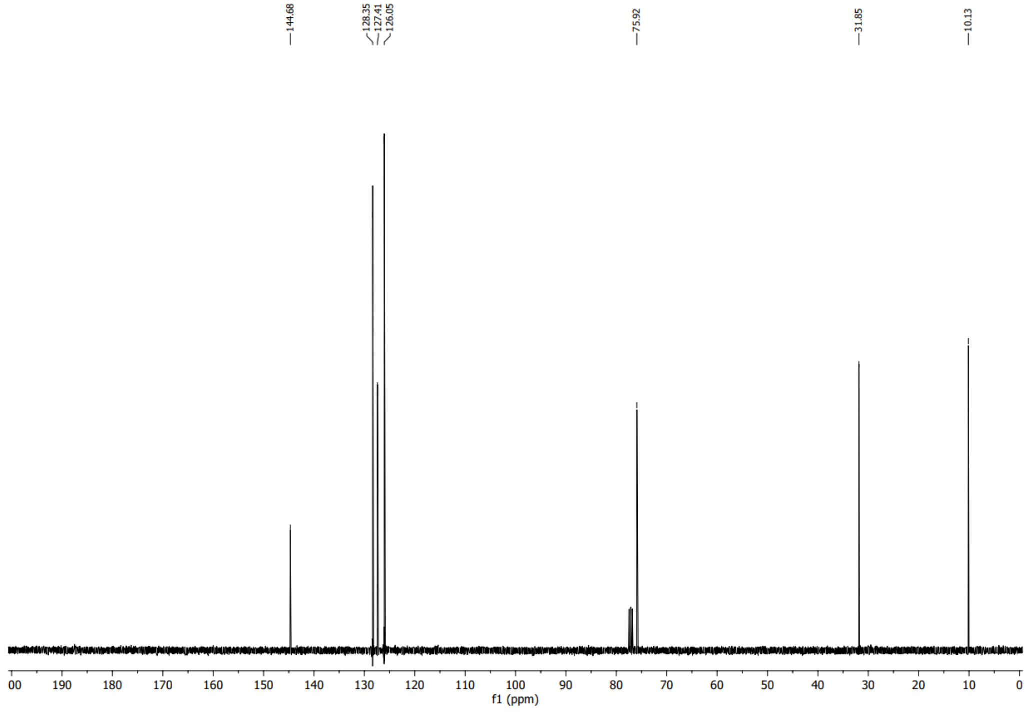

^{13}CNMR (100 MHz; CDCl3) spectrum of compound **1b**.

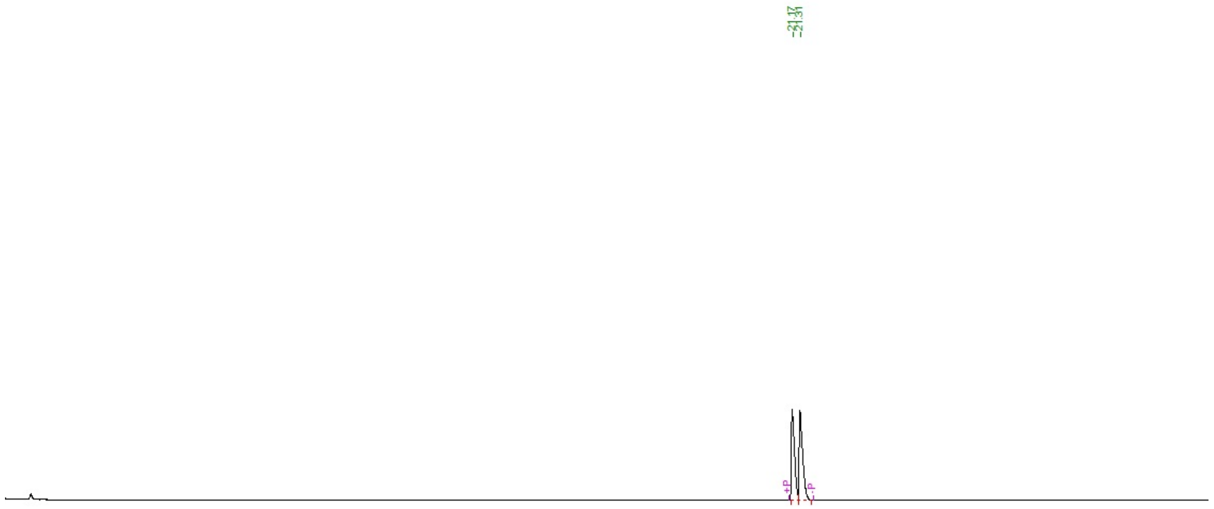

GC chromatogram (general GC method 1) of racemic compound **1b**.

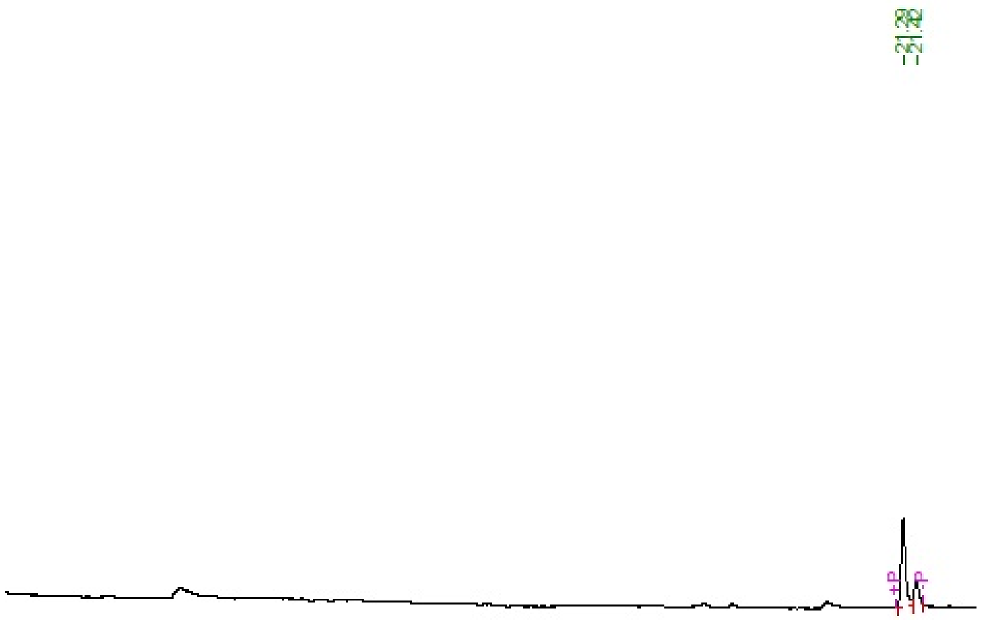

GC chromatogram (general GC method 1) of (*R*)- enantio enriched 57 % *ee* compound **1b**.

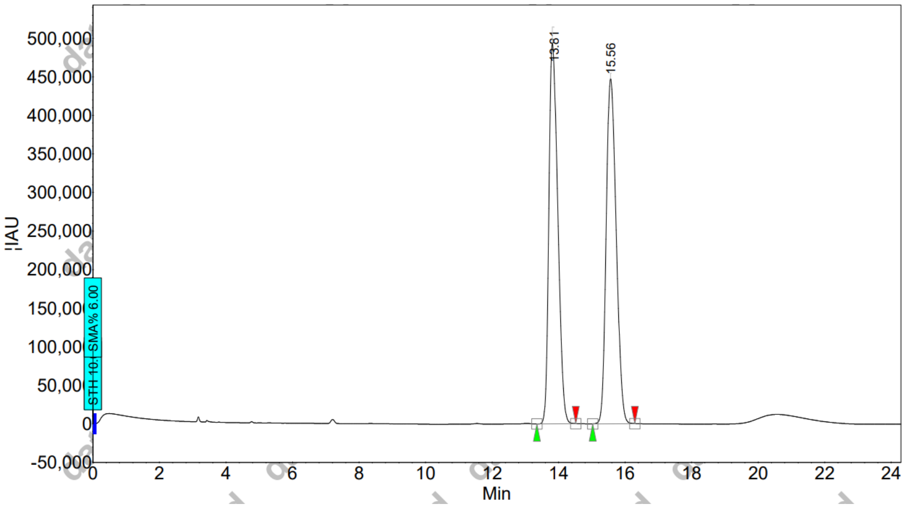

HPLC chromatogram (hexane:isopropanol 97:3, OM) of racemic compound **1b** obtained according to general method.

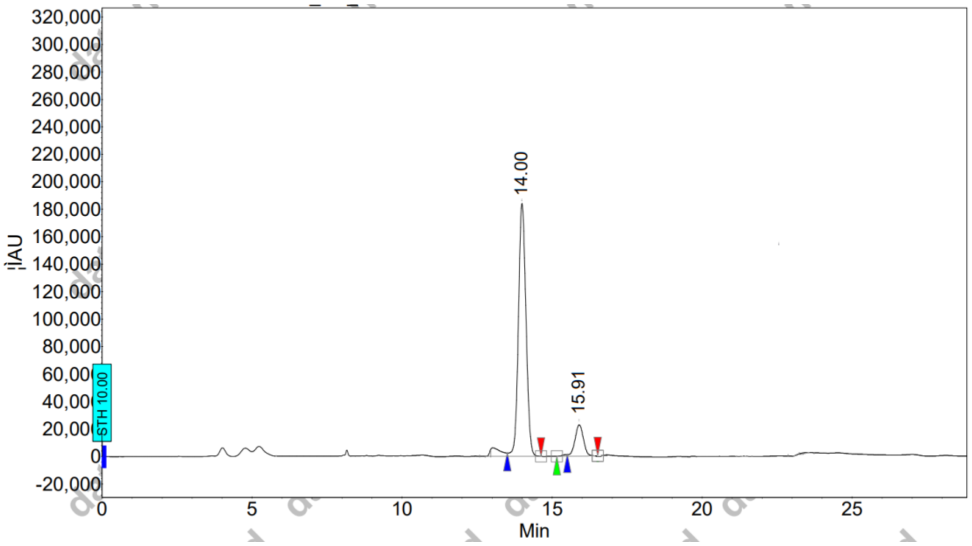

HPLC chromatogram (hexane:isopropanol 97:3, OM) of (*R*)-optically enriched (76 % *ee*) compound **1b** obtained according to general method.

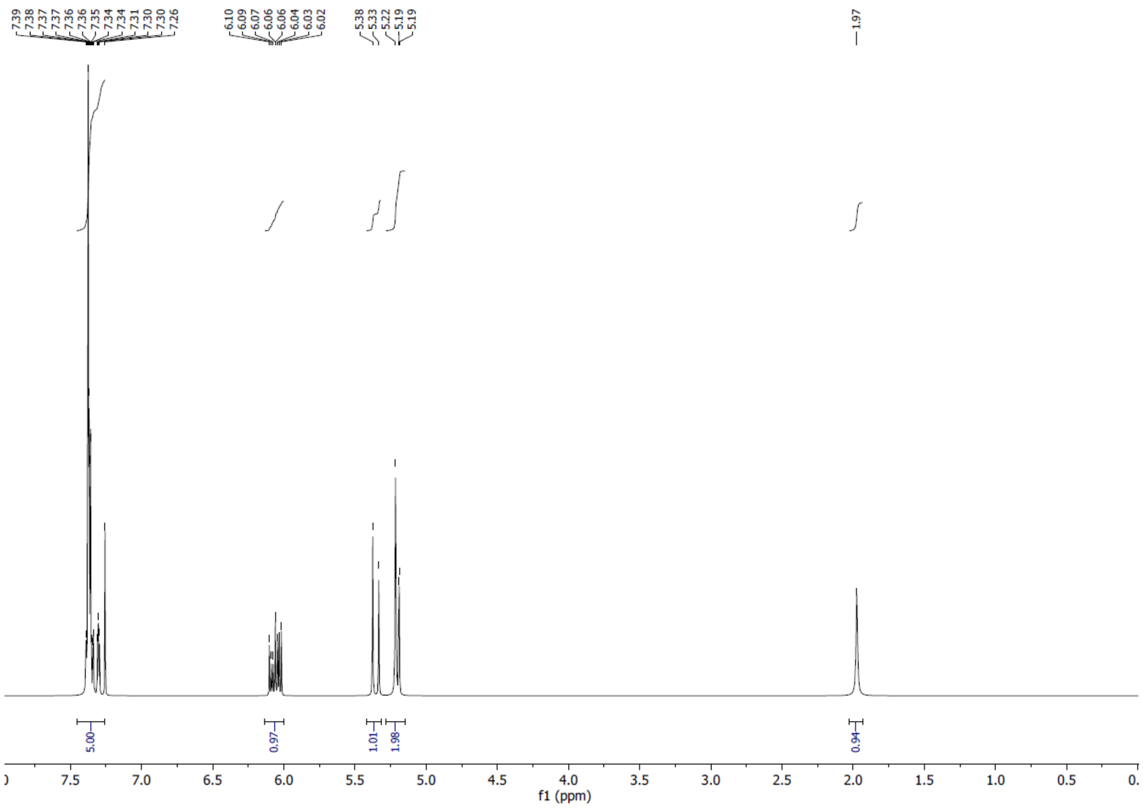

^{1}HNMR (400 MHz; $CDCl_3$) spectrum of compound **1c**.

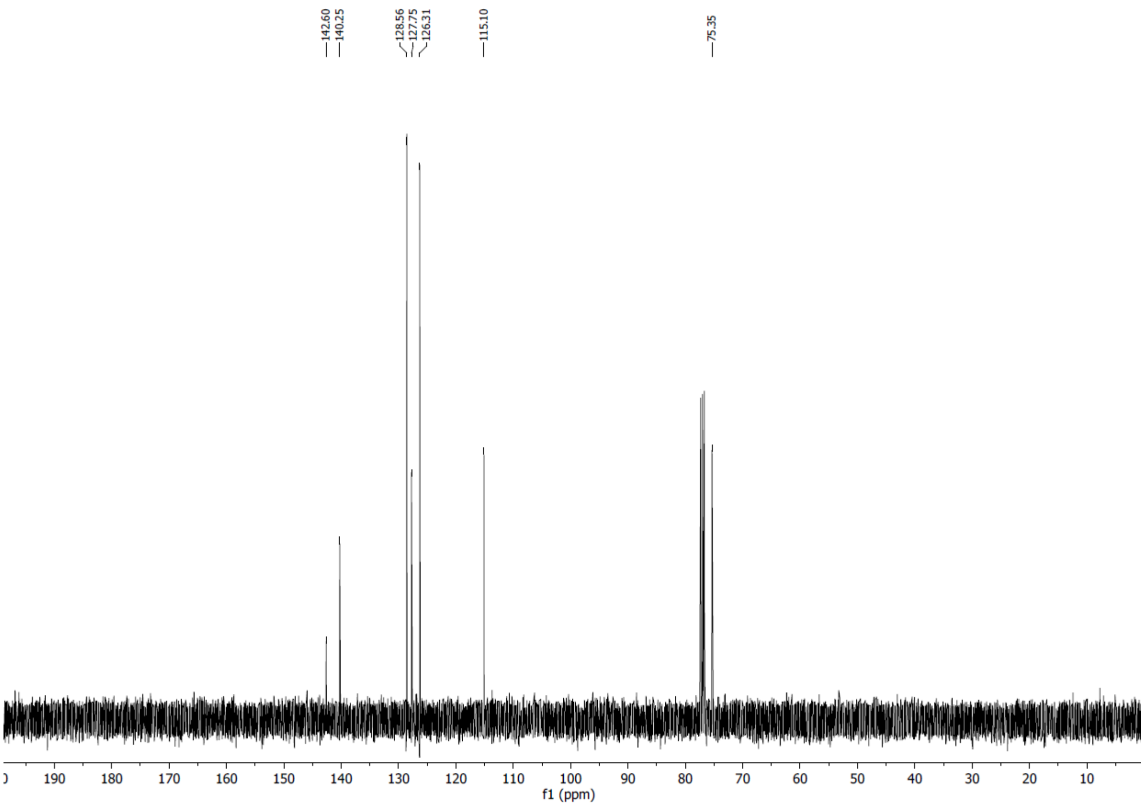

^{13}CNMR (100 MHz; $CDCl_3$) spectrum of compound **1c**.

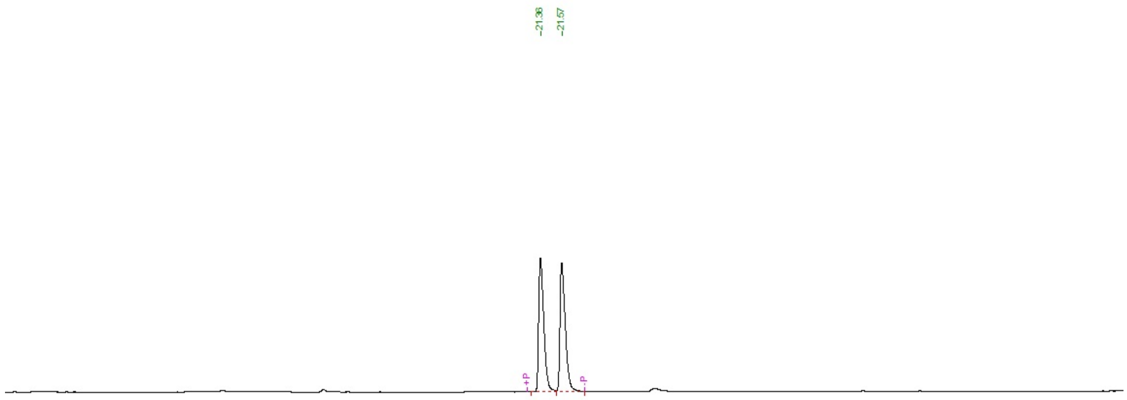

GC chromatogram (general GC method 2) of racemic compound **1c**.

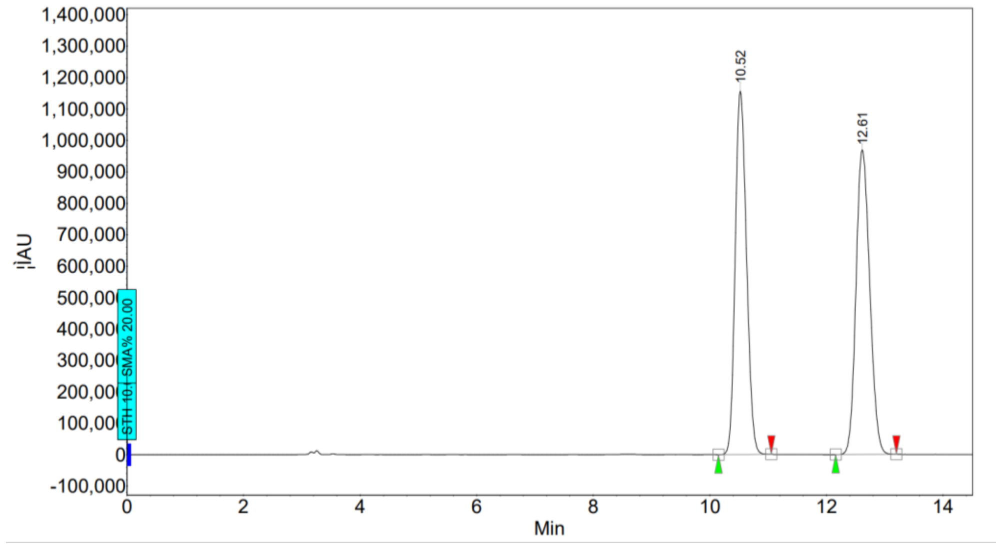

HPLC chromatogram (hexane:isopropanol 95:5; OM) of racemic compound **1c** obtained according to general method.

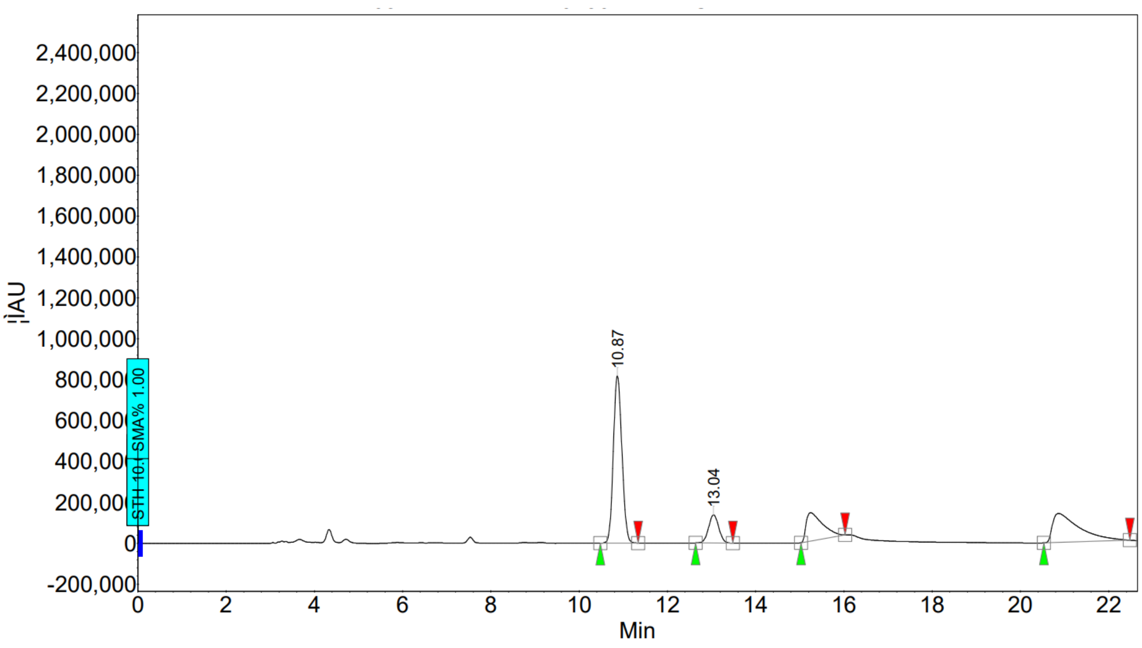

HPLC chromatogram (hexane:isopropanol 95:5, OM) of (*R*)-optically enriched (69 % *ee*) compound **1c** obtained according to general method.

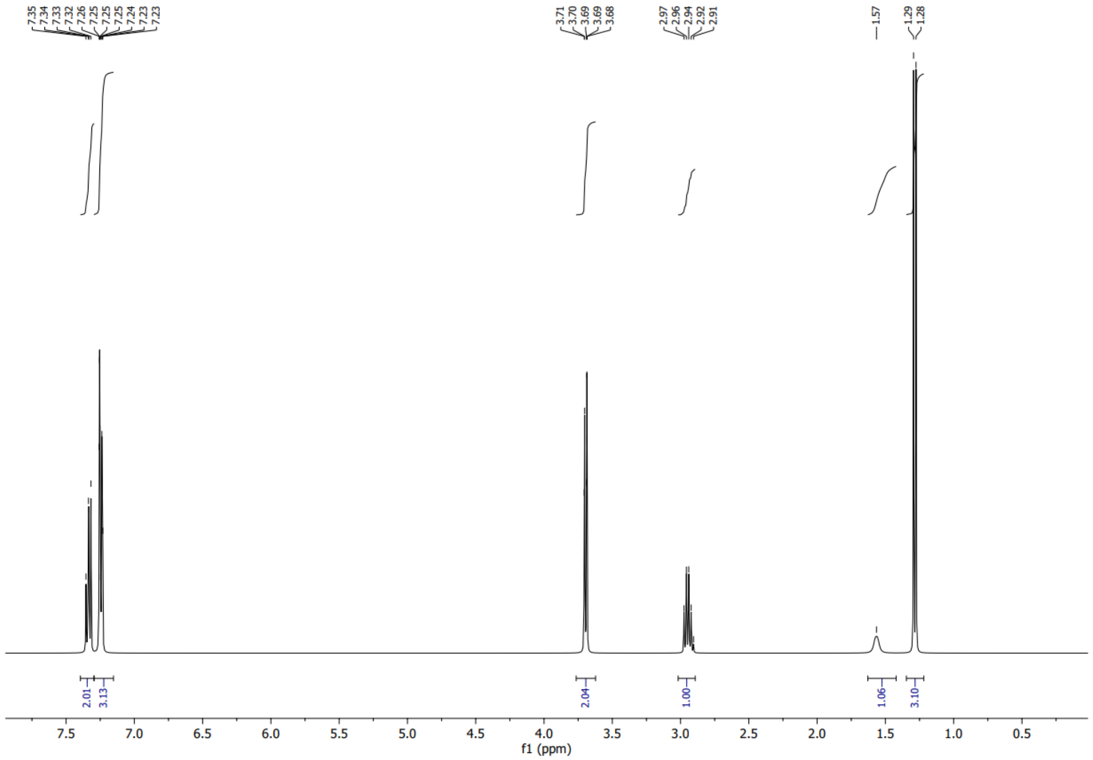

^{1}HNMR (400 MHz; $CDCl_3$) spectrum of compound **1d**.

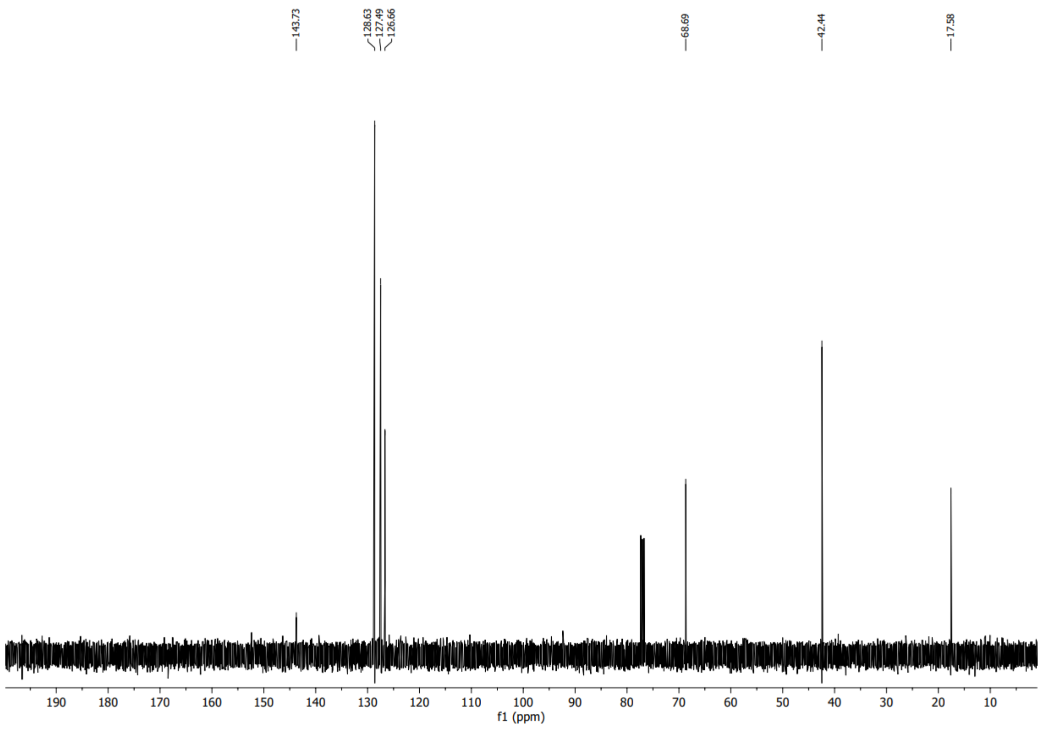

^{13}CNMR (100 MHz; $CDCl_3$) spectrum of compound **1d**.

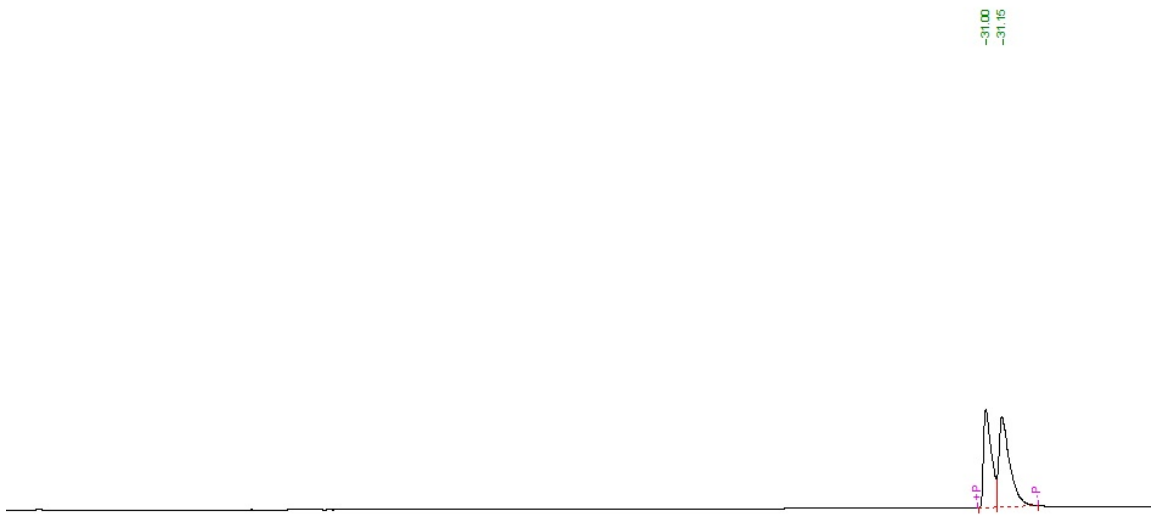

GC chromatogram (general GC method 2) of racemic compound **1d**.

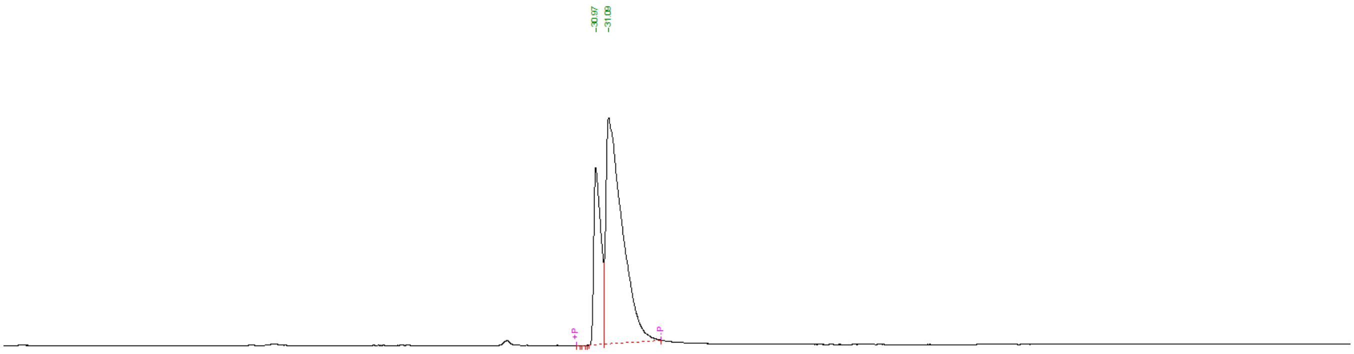

GC chromatogram (general GC method 2) of (*S*) - enantiomerically enriched (33 % *ee*)

compound **1d**.

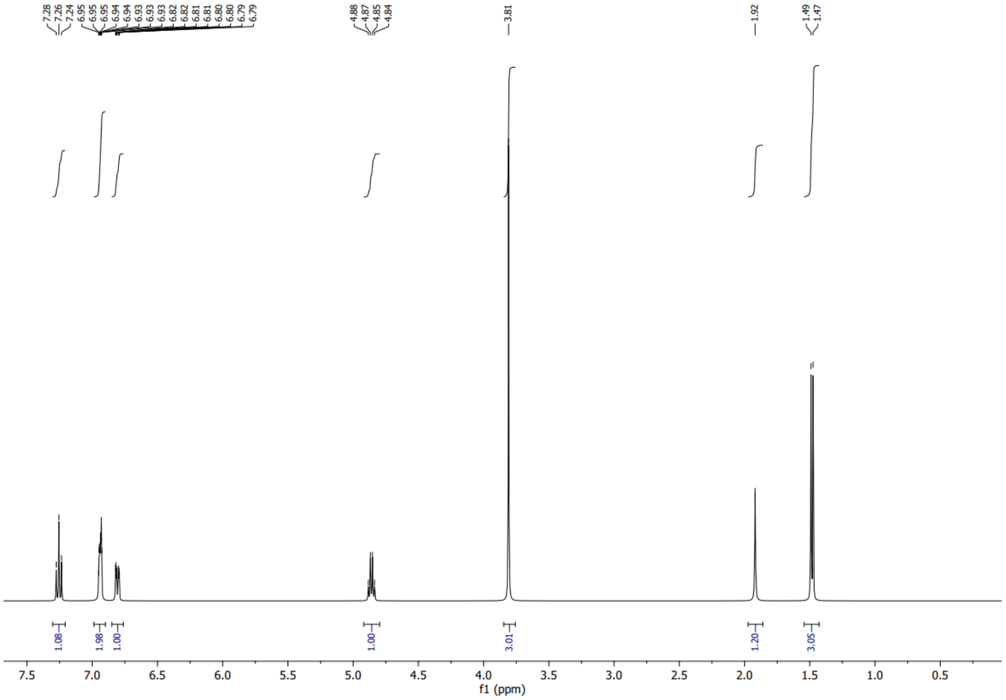

^{1}HNMR (400 MHz; $CDCl_3$) spectrum of compound **1e**.

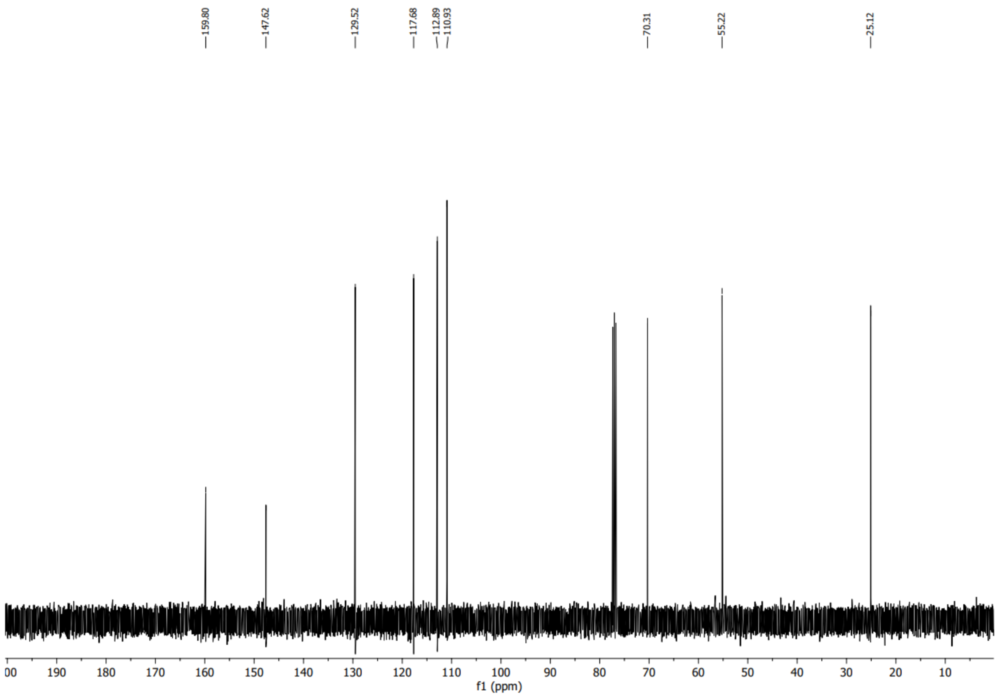

^{13}CNMR (100 MHz; $CDCl_3$) spectrum of compound **1e**.

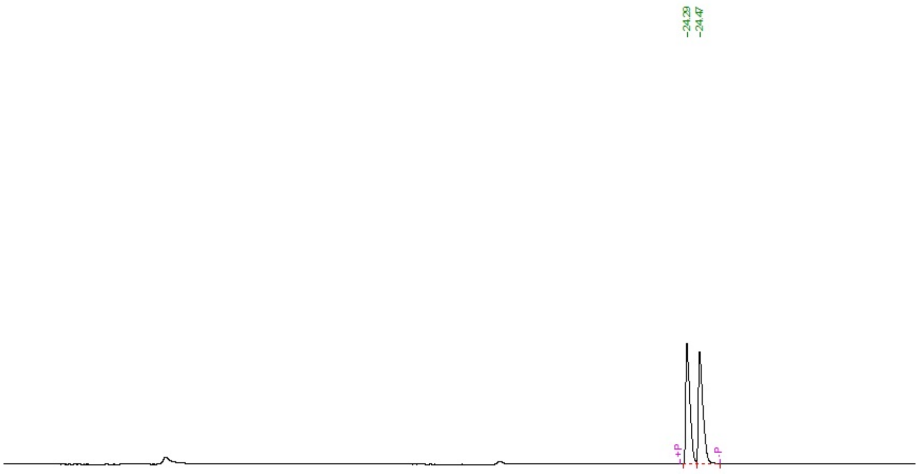

GC chromatogram (general GC method 1) of racemic compound **1e**.

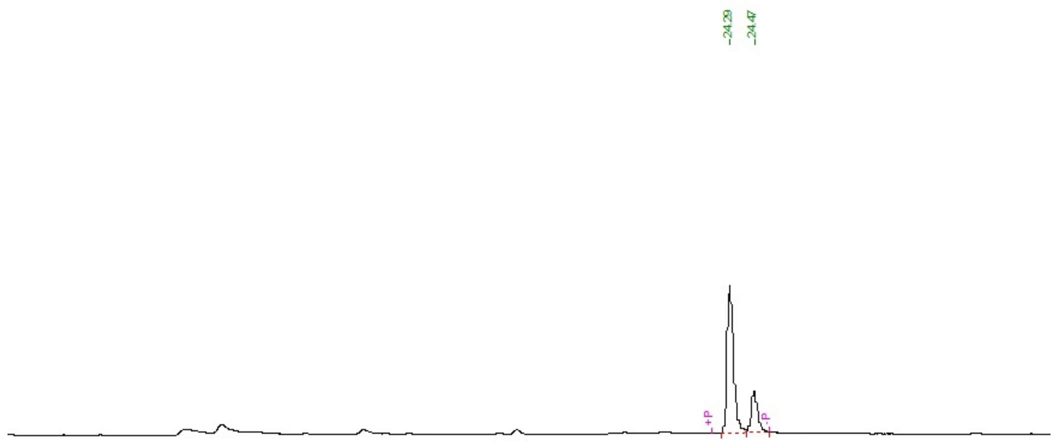

GC chromatogram (general GC method 1) of (*R*)-enantiomerically enriched (50 % *ee*) compound **1e**.

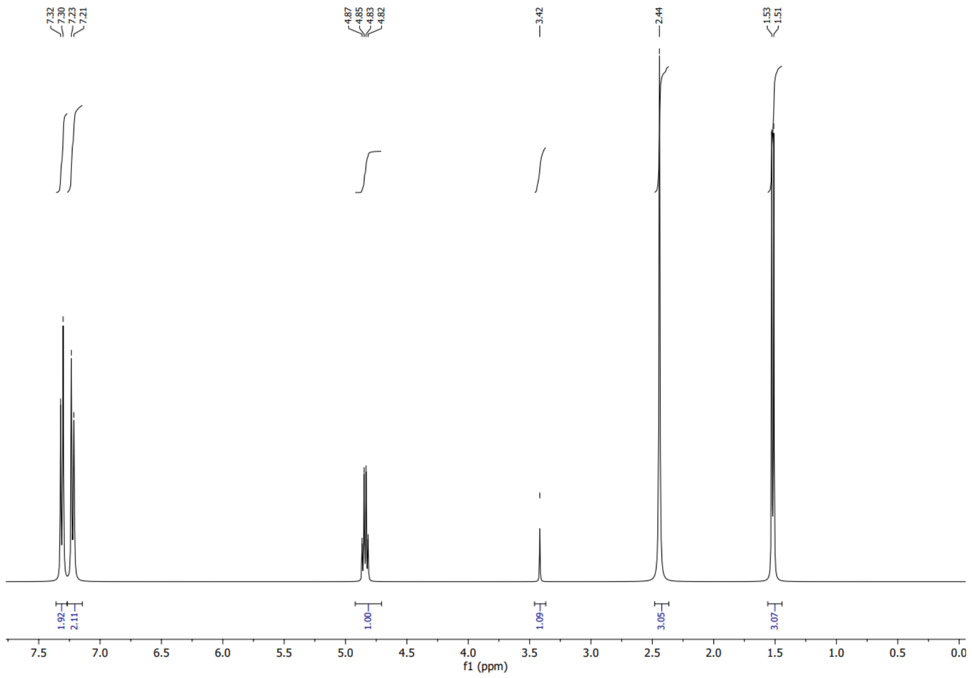

^{1}HNMR (400 MHz; $CDCl_3$) spectrum of compound **1f**.

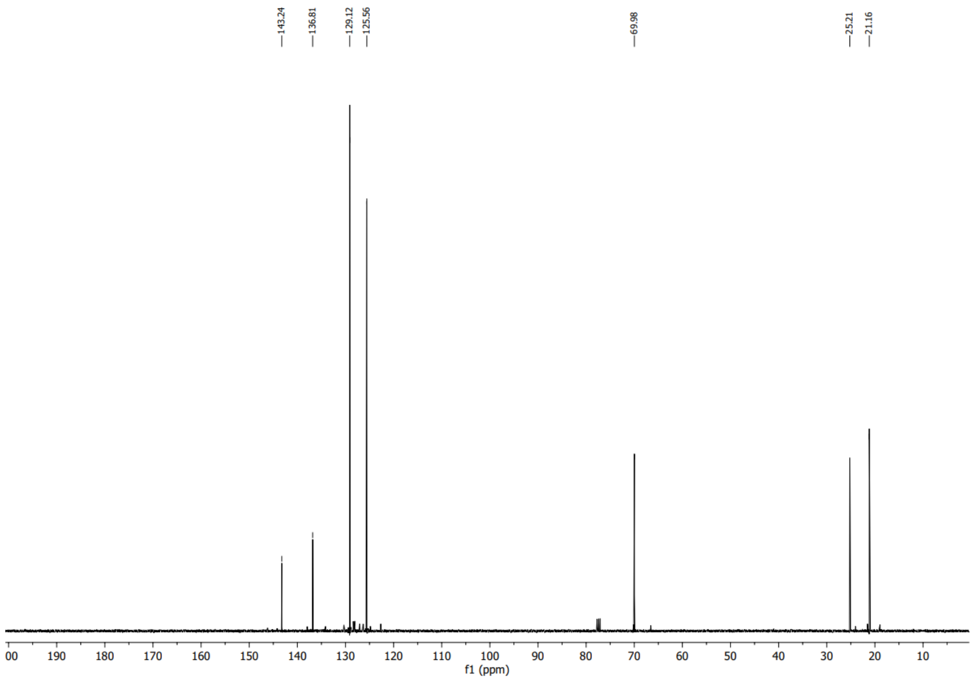

^{13}CNMR (100 MHz; $CDCl_3$) spectrum of compound **1f**.

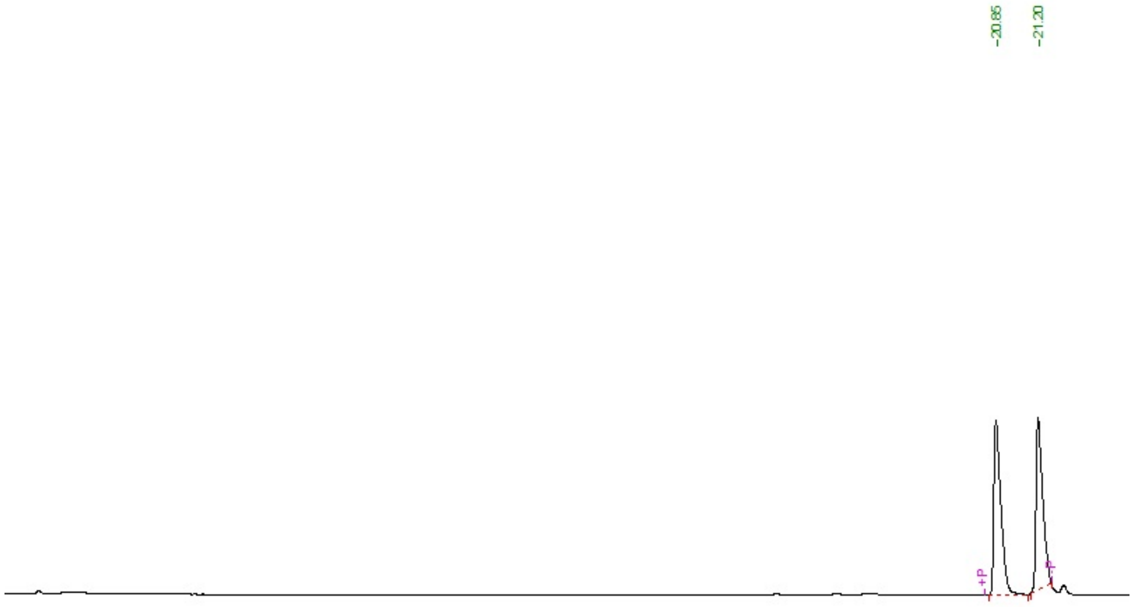

GC chromatogram (general GC method 1) of racemic compound **1f**.

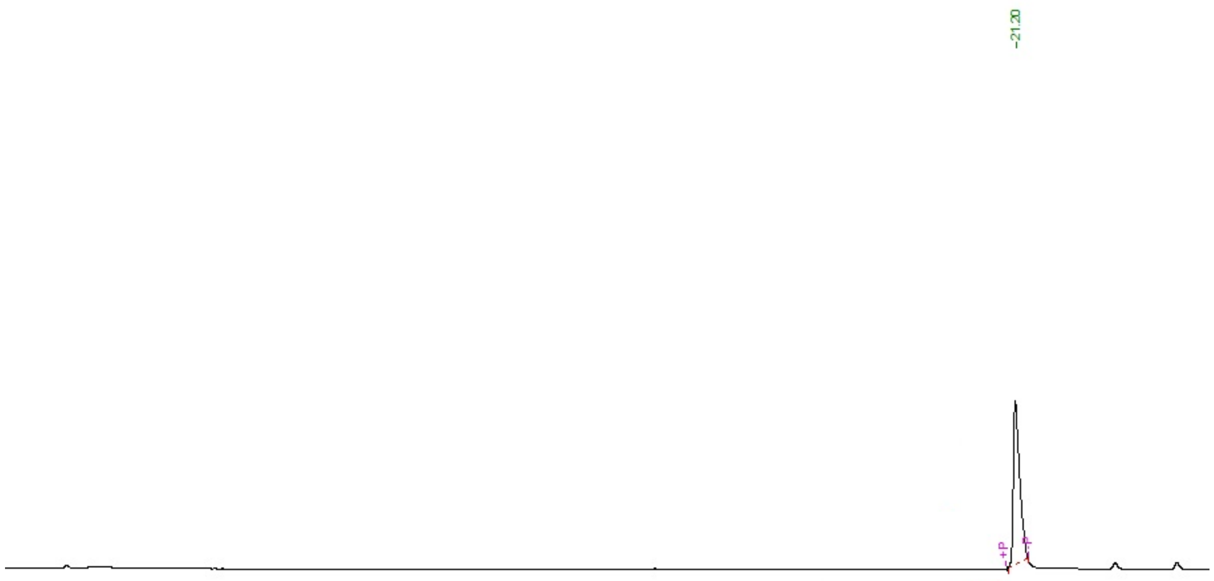

GC chromatogram (general GC method 1) of (*S*)-enantiomer compound **1f**.

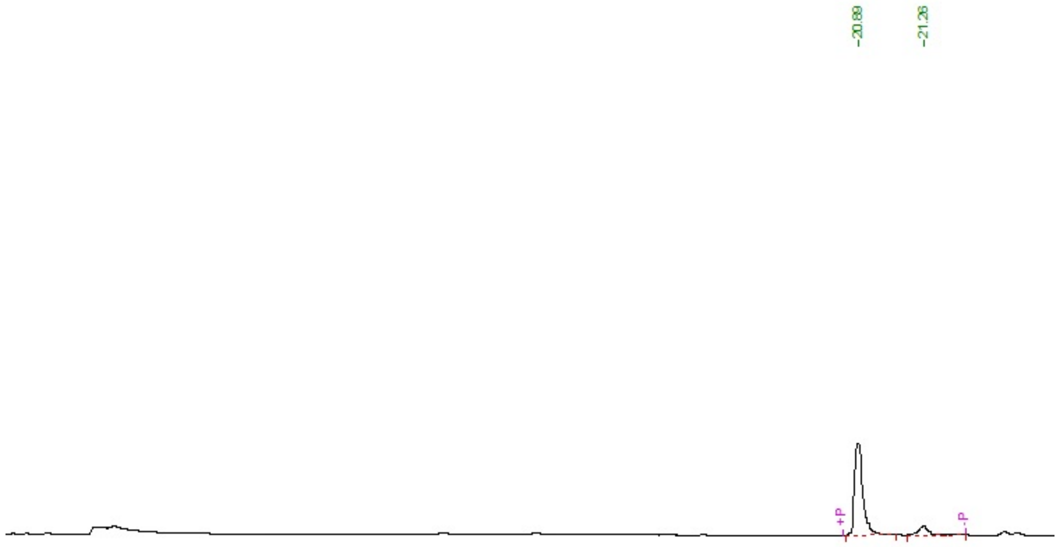

GC chromatogram (general GC method 1) of (*R*)- optically enriched (90 % *ee*) compound **1f**.

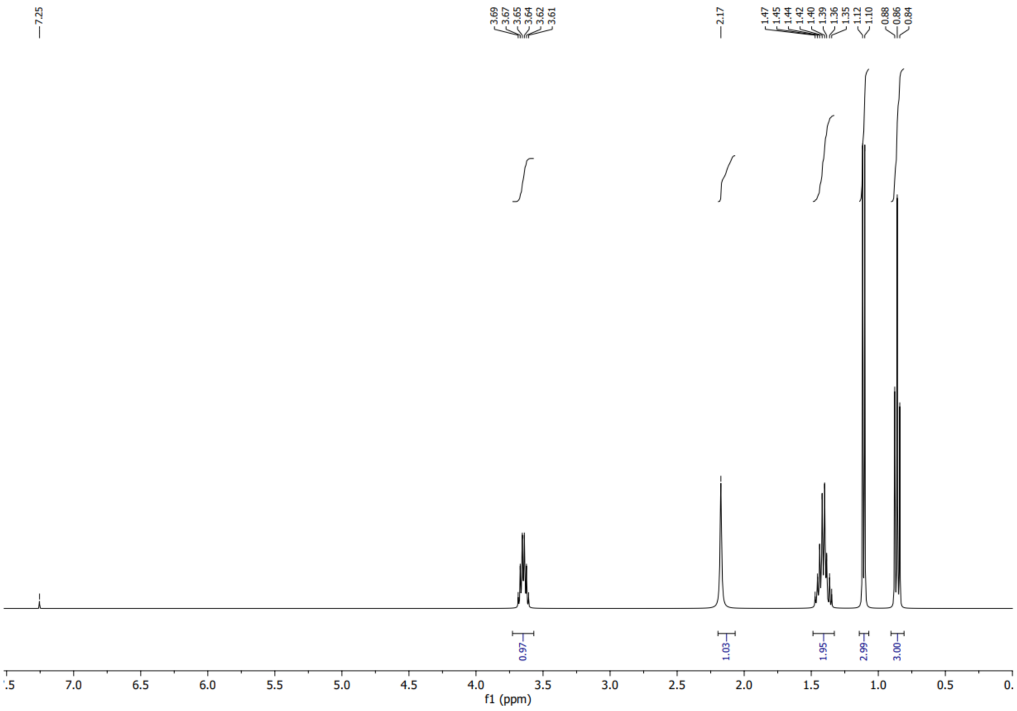

^{1}HNMR (400 MHz; $CDCl_3$) spectrum of compound **1g**.

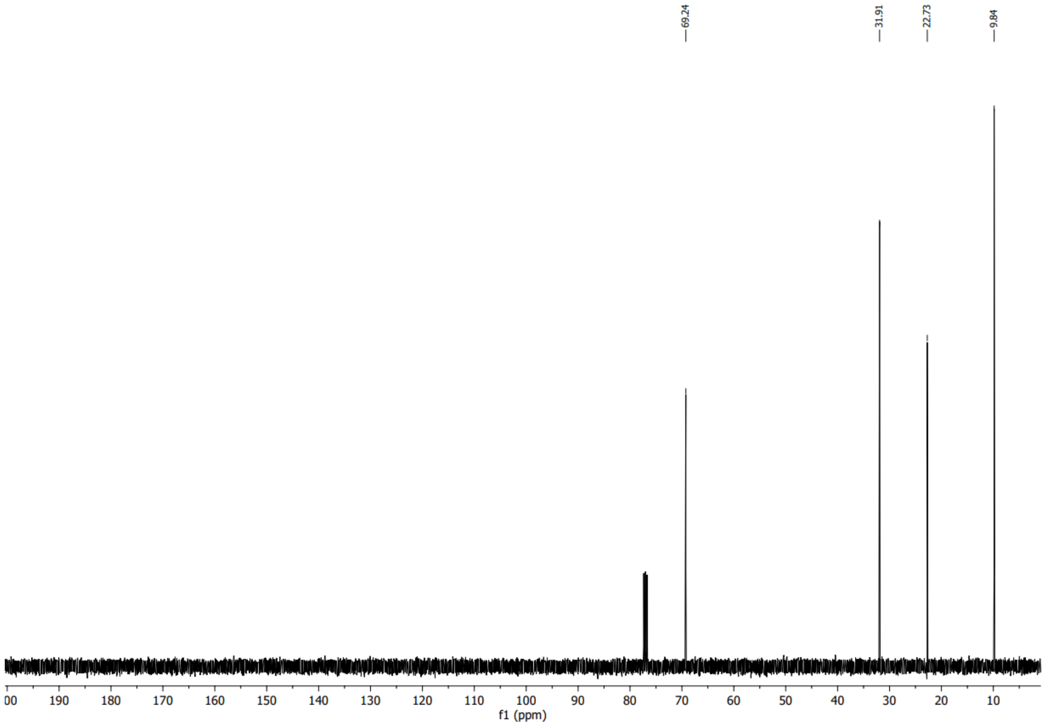

^{13}CNMR (100 MHz; $CDCl_3$) spectrum of compound **1g**.

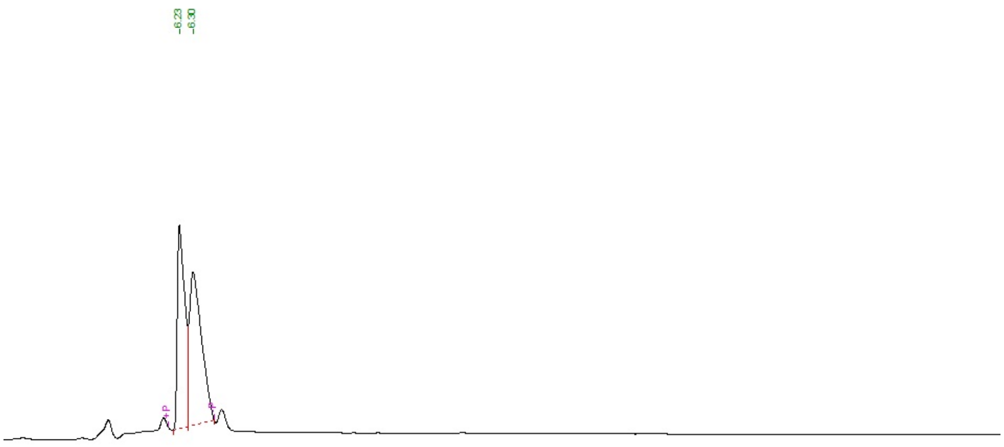

GC chromatogram (general GC method 1) of racemic compound **1g**.

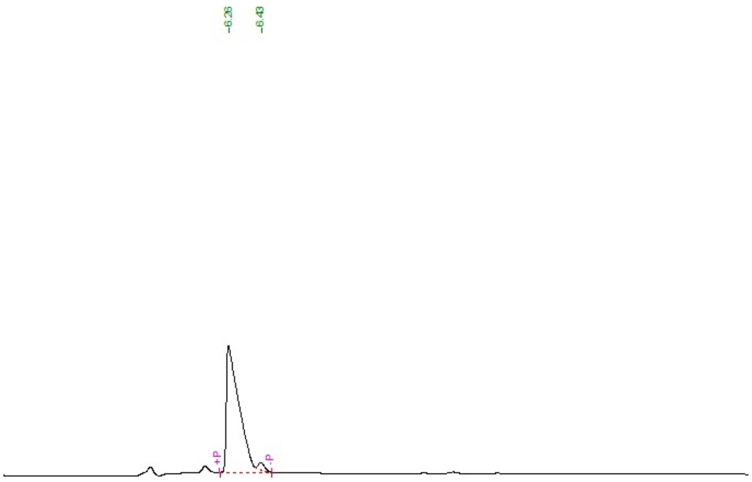

GC chromatogram (general GC method 1) of (*S*)-enantiomer of compound **1g**.

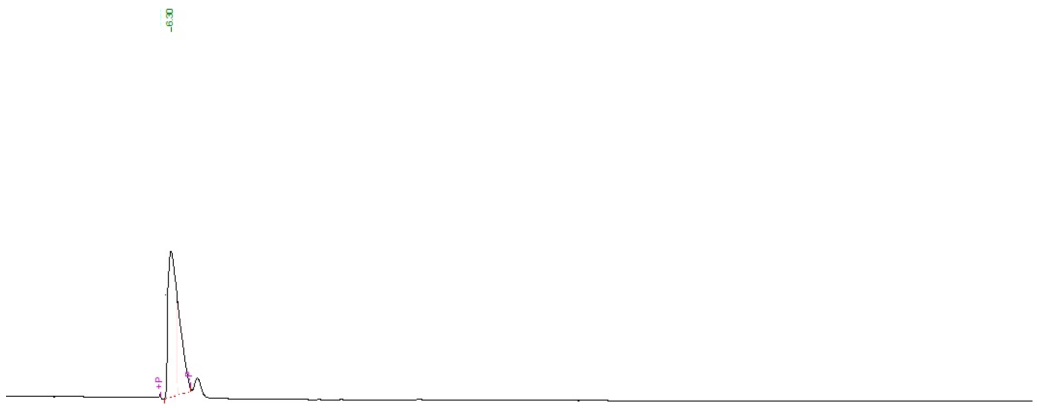

GC chromatogram (general GC method 1) of (*R*)-enantiomer of compound **1g**.

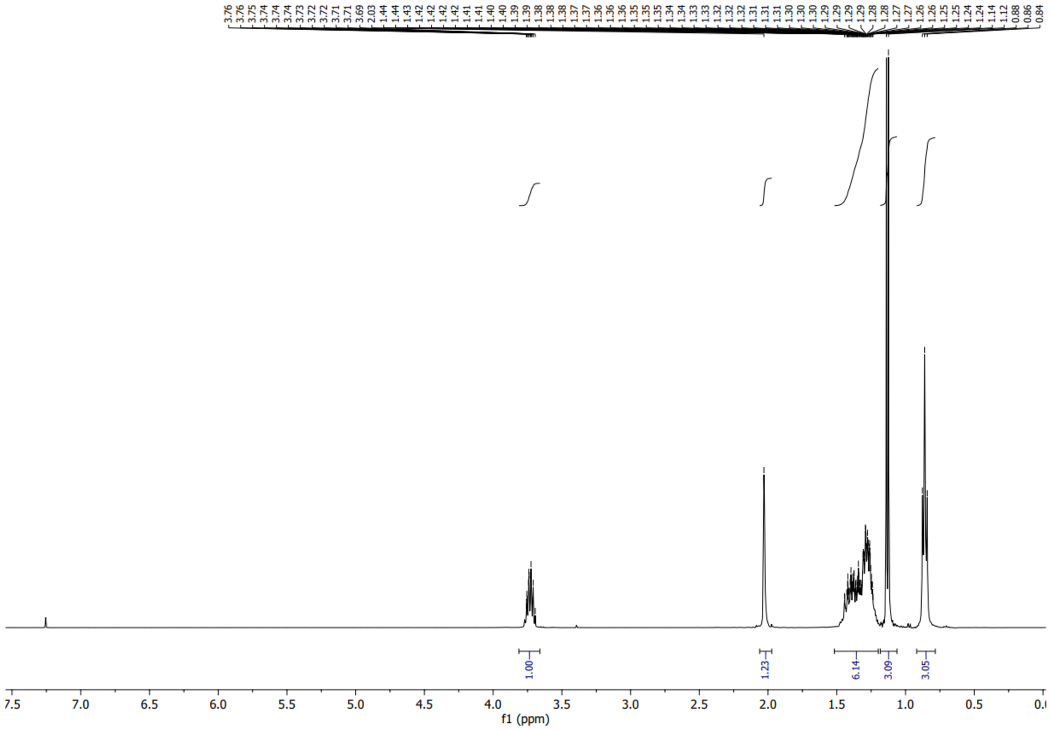

^{1}HNMR (400 MHz; $CDCl_3$) spectrum of compound **1h**.

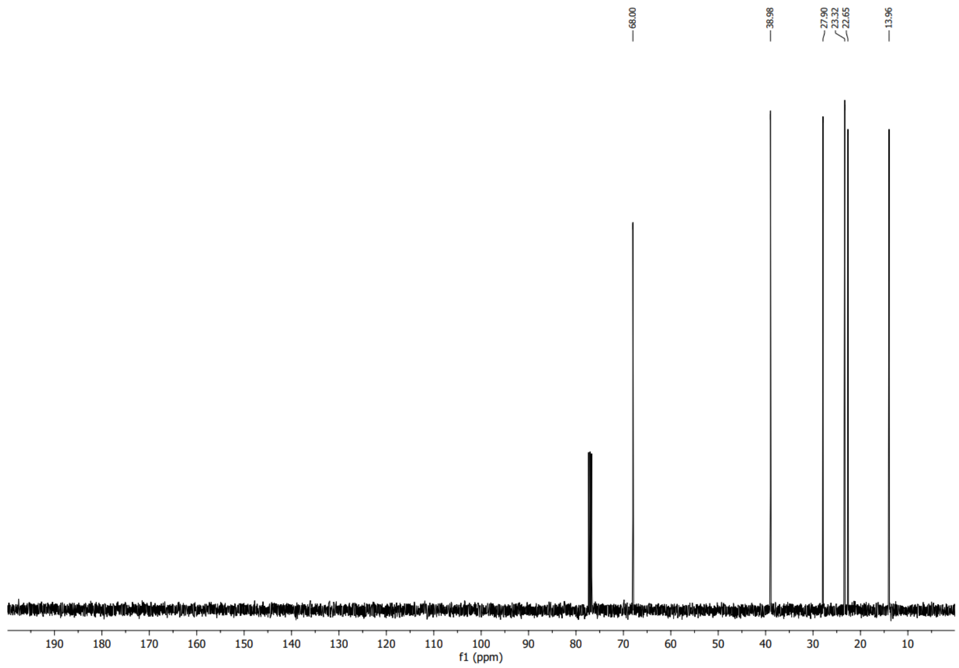

^{13}CNMR (100 MHz; $CDCl_3$) spectrum of compound **1h**.

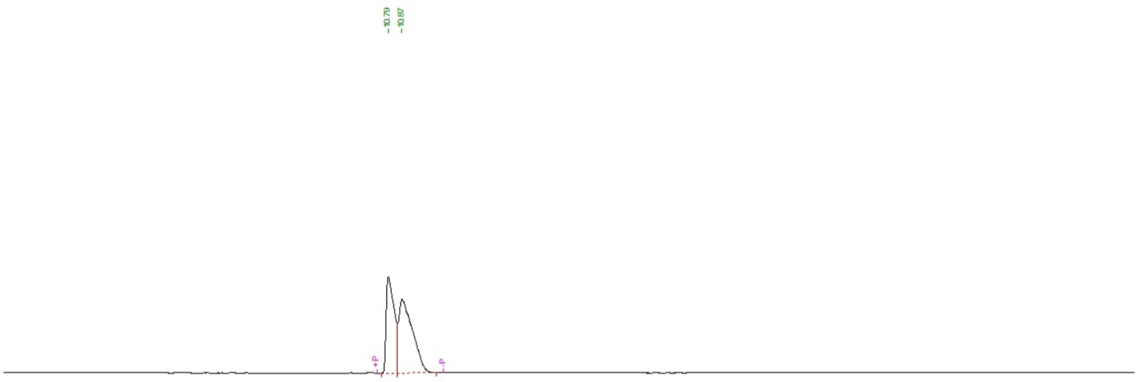

GC chromatogram (general GC method 1) of racemic compound **1h**.

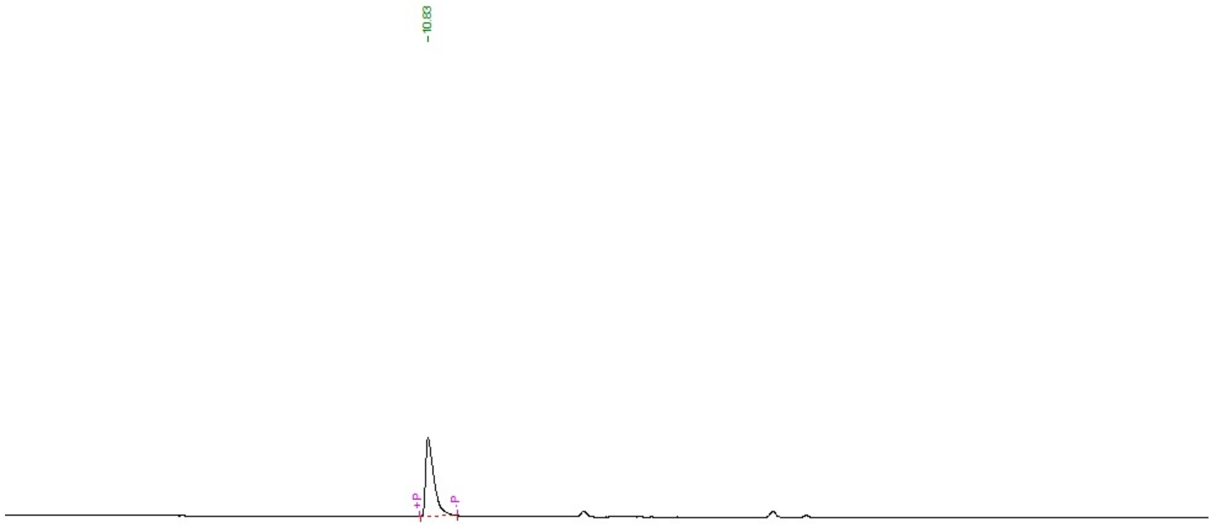

GC chromatogram (general GC method 1) of (*R*)—enantiomer of compound **1h**.

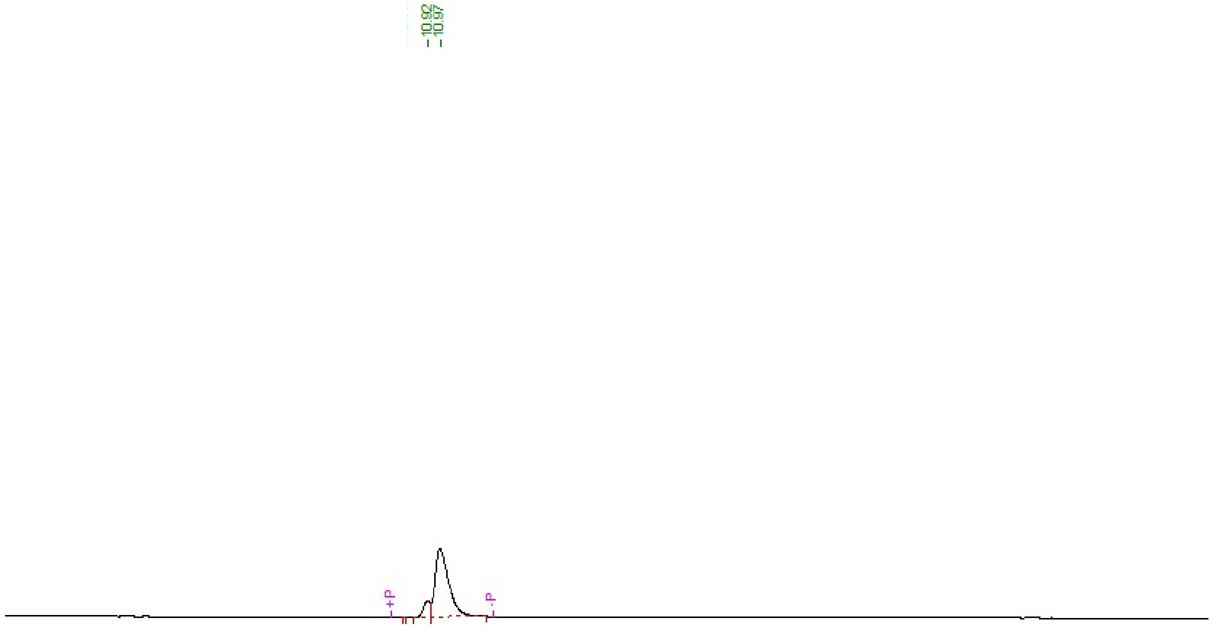

GC chromatogram (general GC method 1) (*S*)—optically enriched (78 % *ee*) compound **1h**.

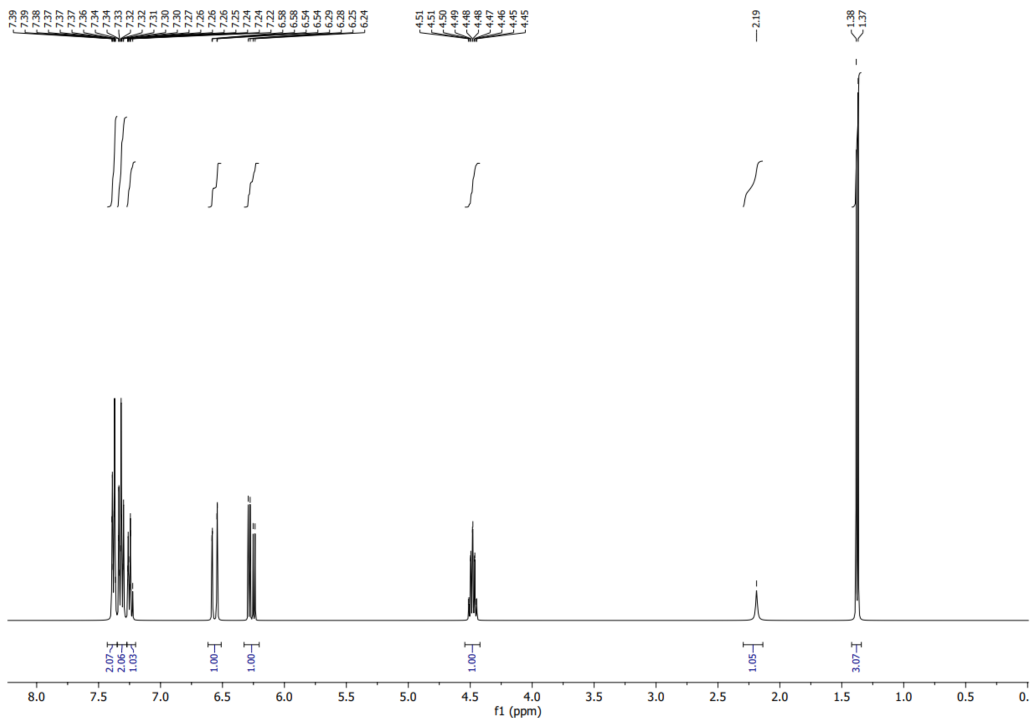

^{1}HNMR (400 MHz; $CDCl_3$) spectrum of compound **1i**.

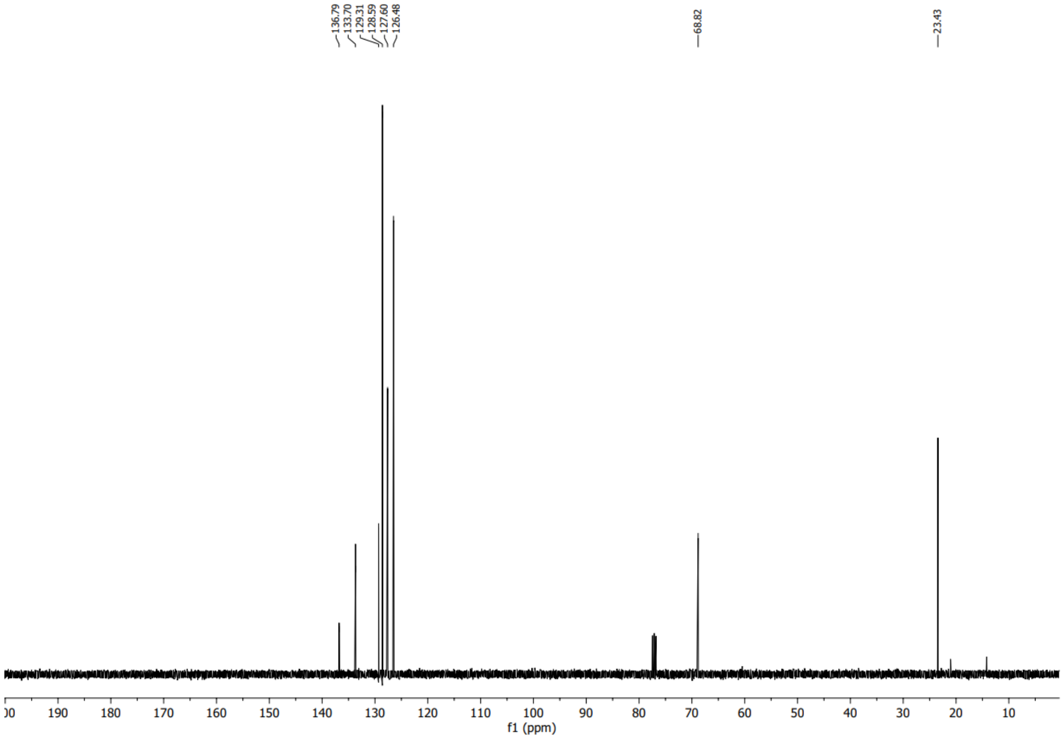

^{13}CNMR (100 MHz; $CDCl_3$) spectrum of compound **1i**.

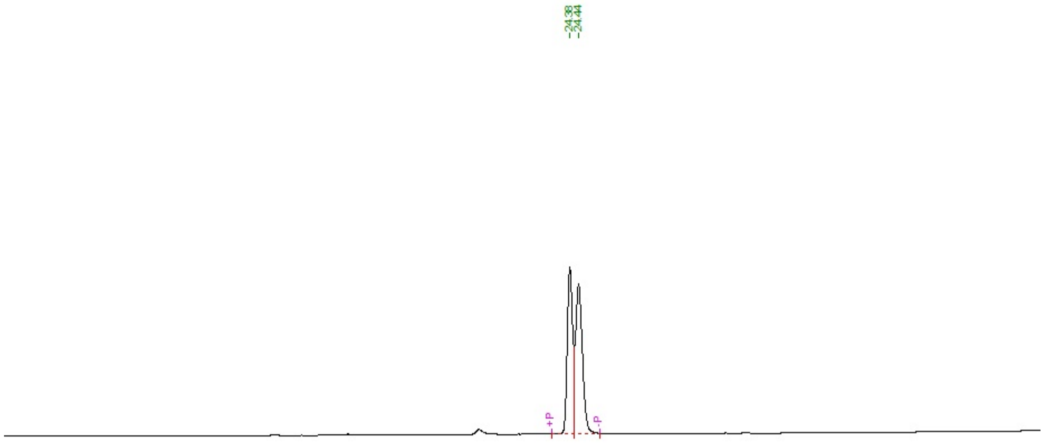

GC chromatogram (general GC method 1) of racemic compound **1i**.

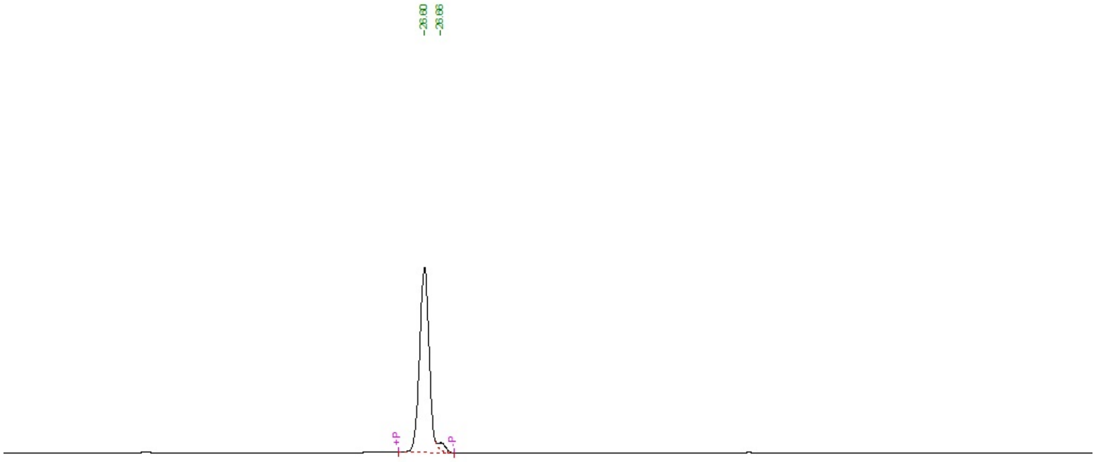

GC chromatogram (general GC method 1) of (*R*)-optically enriched (97 % *ee*) compound **1i.**

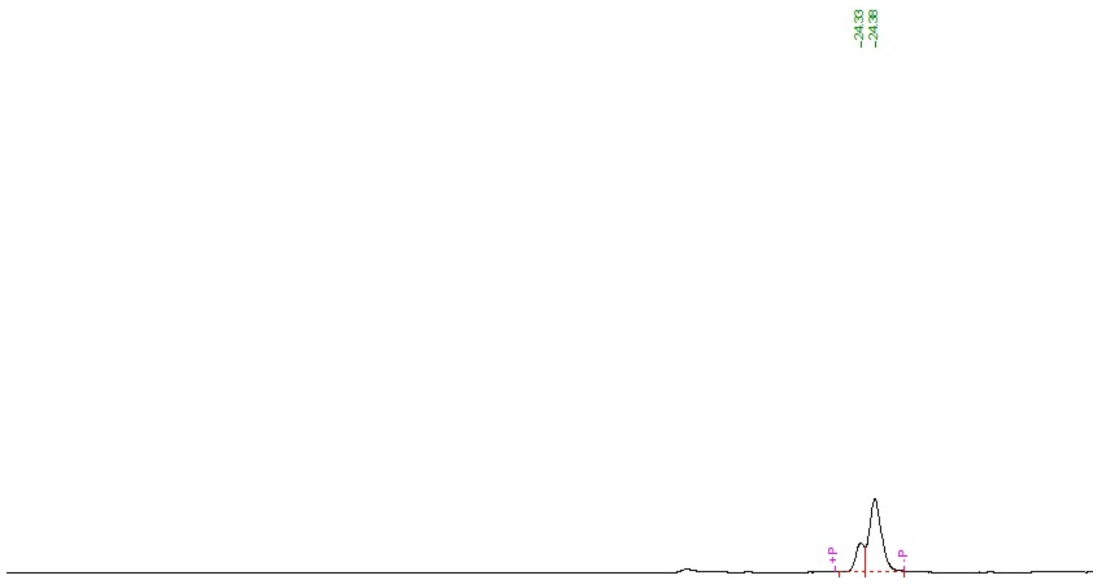

GC chromatogram (general GC method 1) of (*S*)-optically enriched (67 % *ee*) compound **1i**.

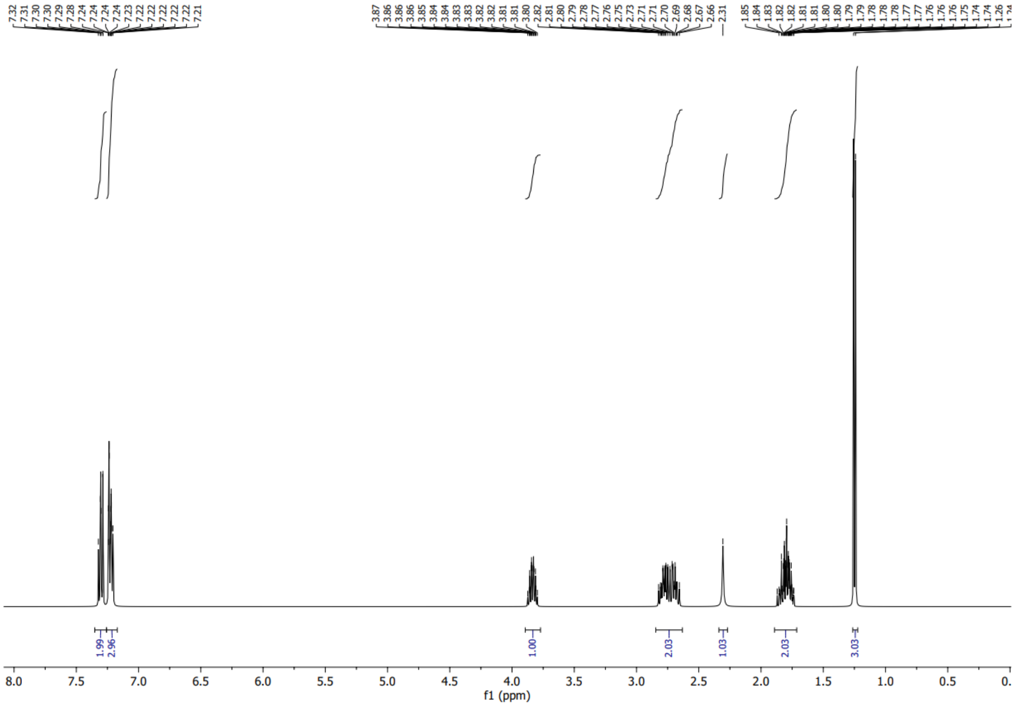

^{1}HNMR (400 MHz; $CDCl_3$) spectrum of compound **1j**.

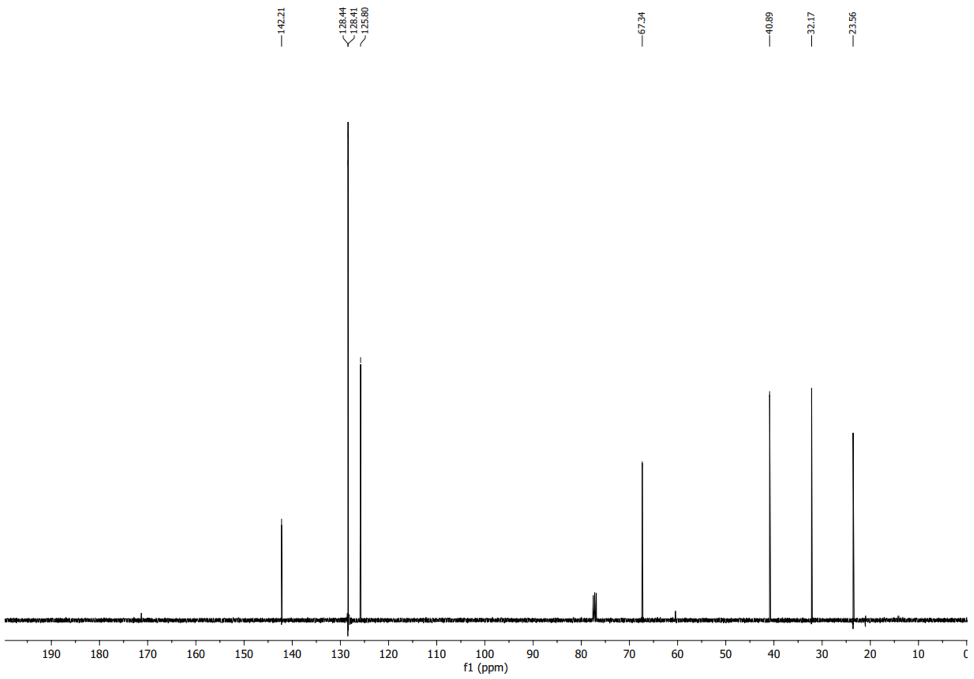

^{13}CNMR (100 MHz; $CDCl_3$) spectrum of compound **1j**.

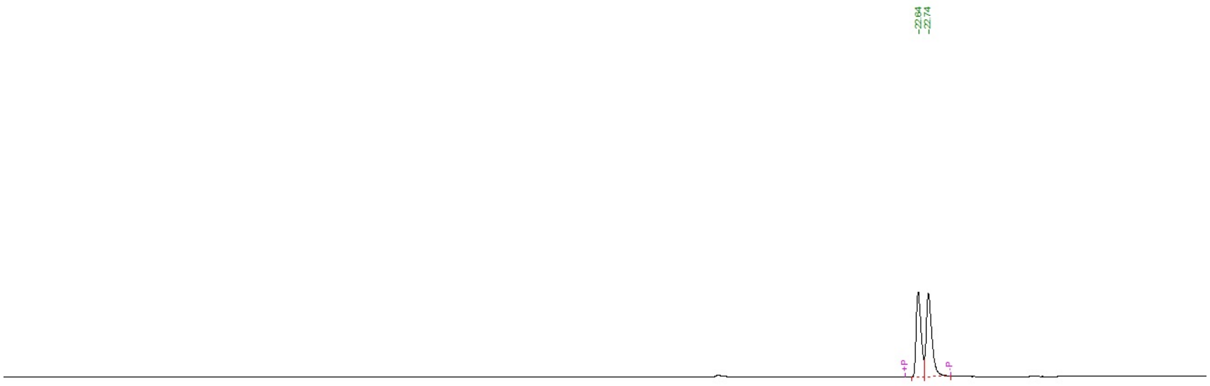

GC chromatogram (general GC method 1) of racemic compound **1j**.

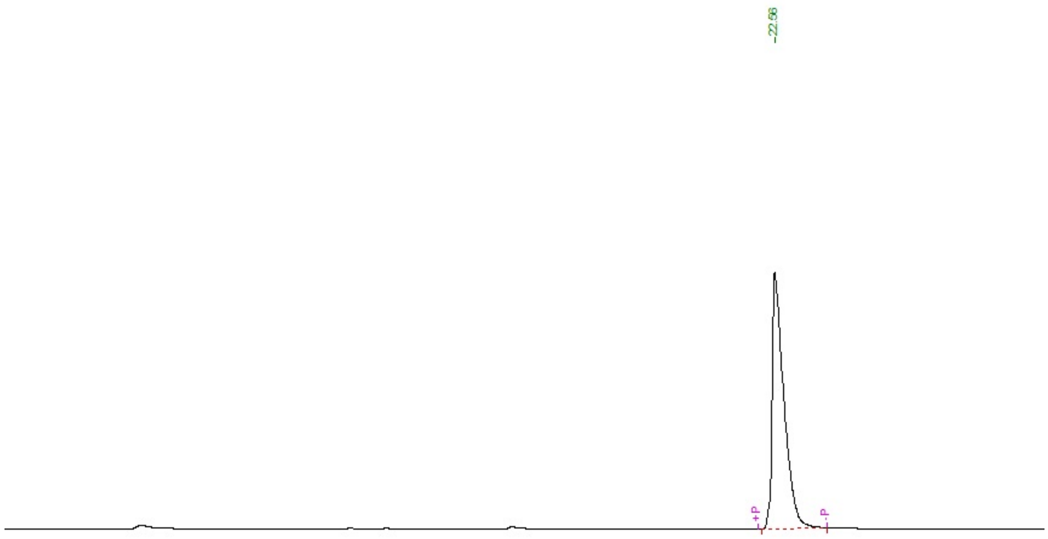

GC chromatogram (general GC method 1) of (*R*) enantiomer compound **1j**.

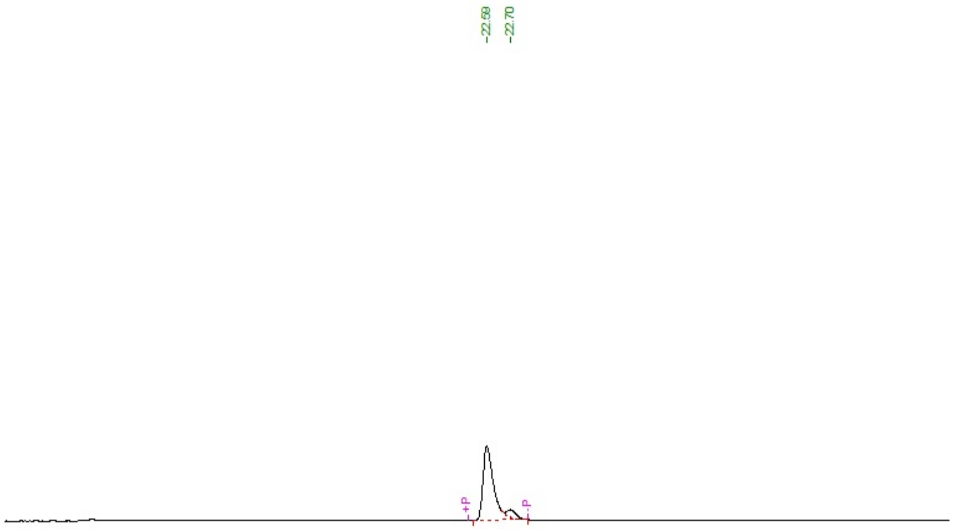

GC chromatogram (general GC method 1) of (*S*)-optically enriched (81 % *ee*) compound **1j**.

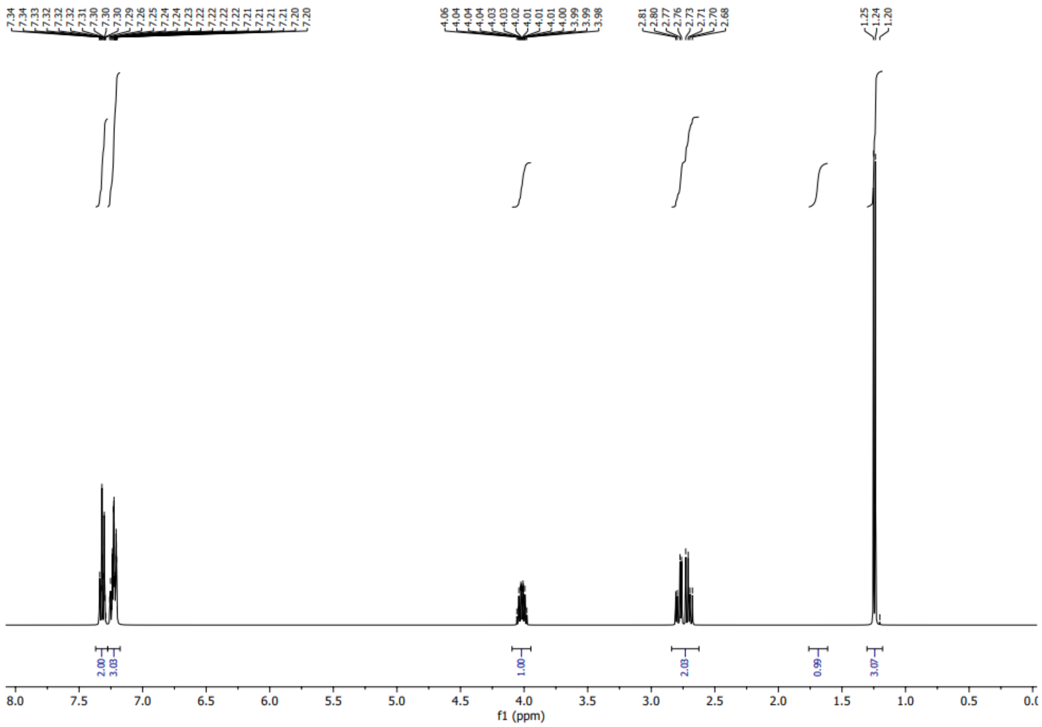

^{1}HNMR (400 MHz; $CDCl_3$) spectrum of compound **1k**.

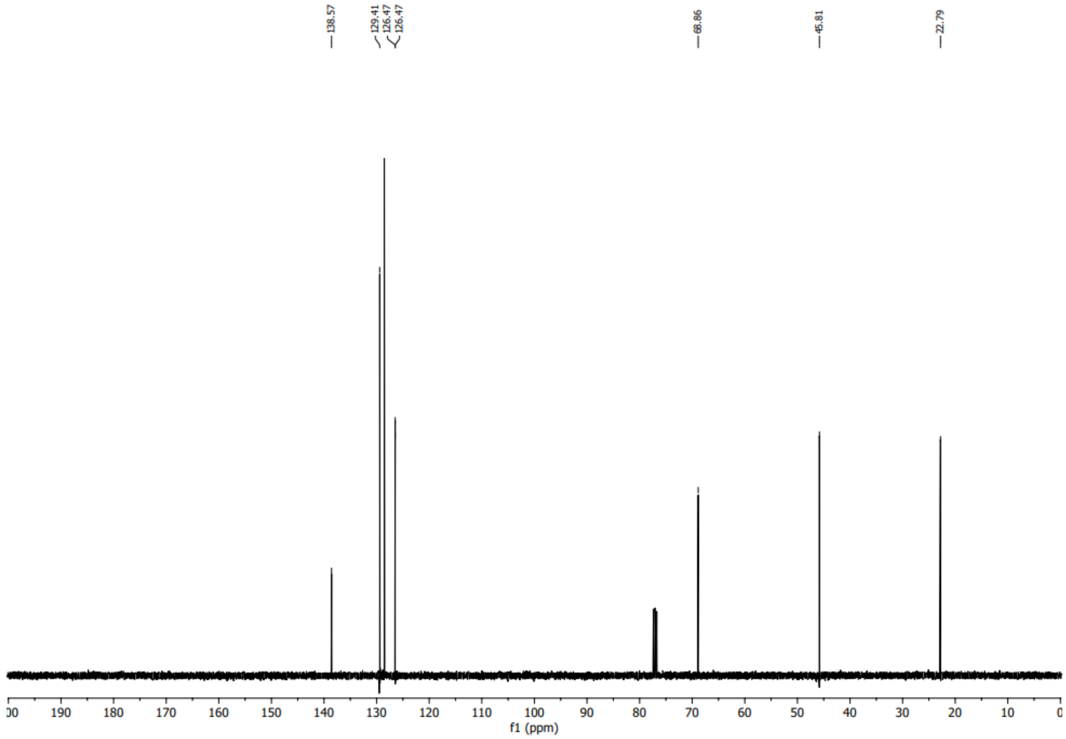

^{13}CNMR (100 MHz; $CDCl_3$) spectrum of compound **1k**.

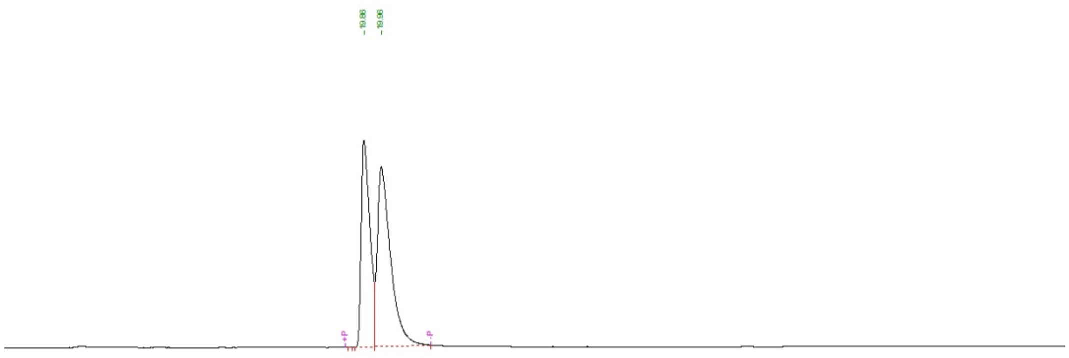

GC chromatogram (general GC method 2) of racemic compound **1k**.

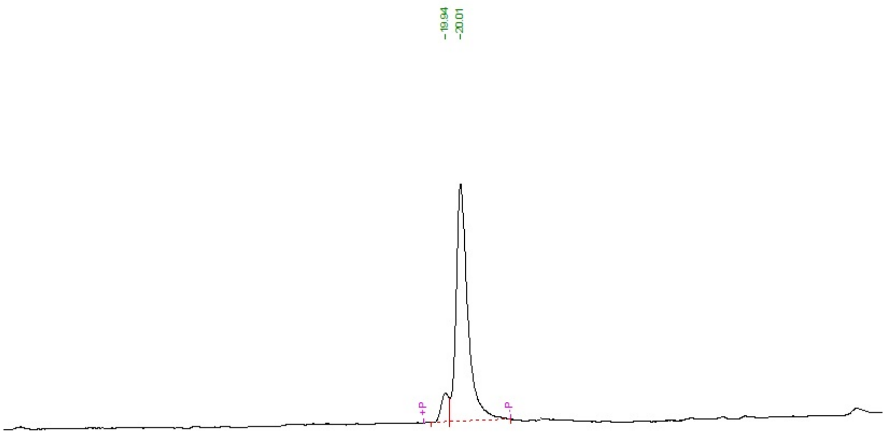

GC chromatogram (general GC method 2) of (*R*)-optically enriched (87 % *ee*) compound **1k** obtained according to general method 1.

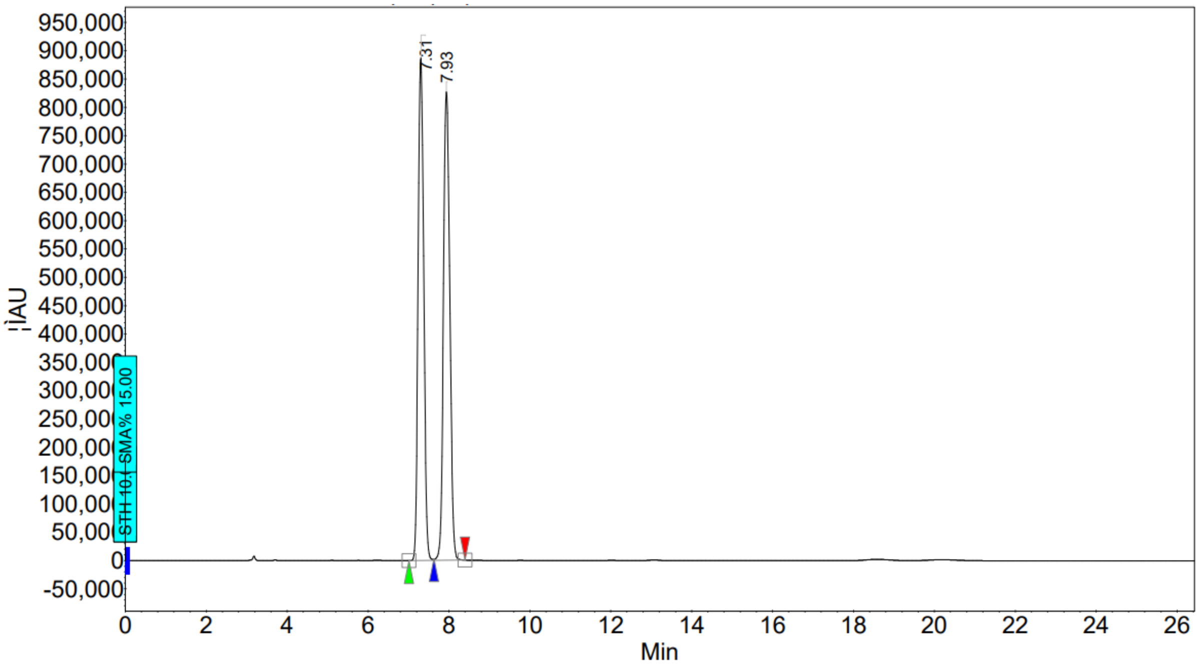

HPLC chromatogram (hexane:isopropanol 94:6, OM) of racemic compound **1k** obtained according to general method.

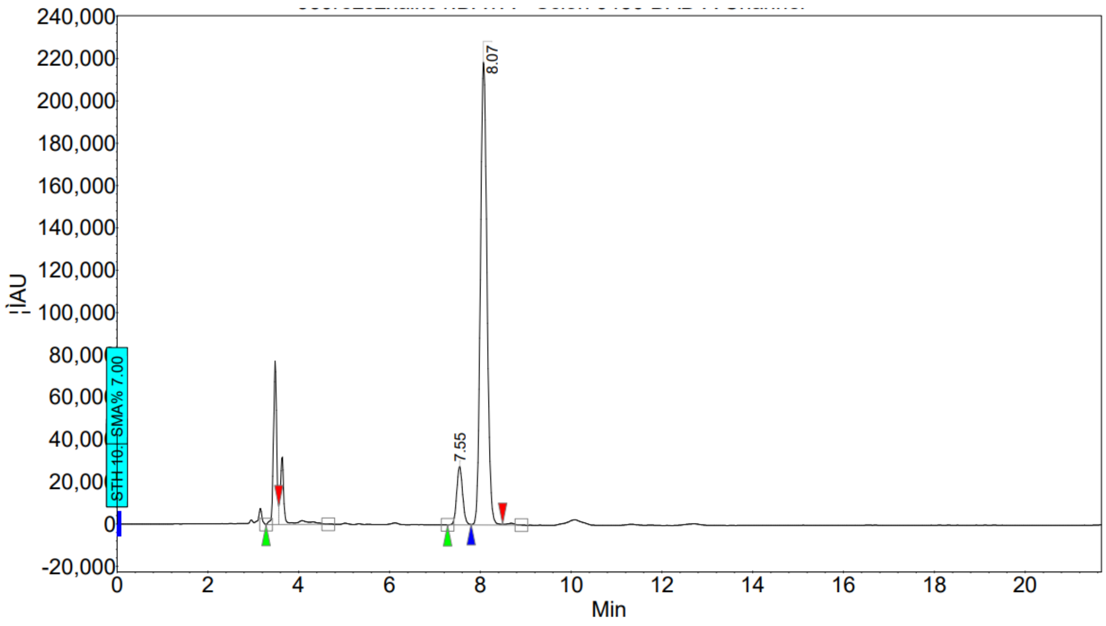

HPLC chromatogram (hexane:isopropanol 95:5, OM) of (*R*)-optically enriched (83 % *ee*) compound **1k** obtained according to general method.

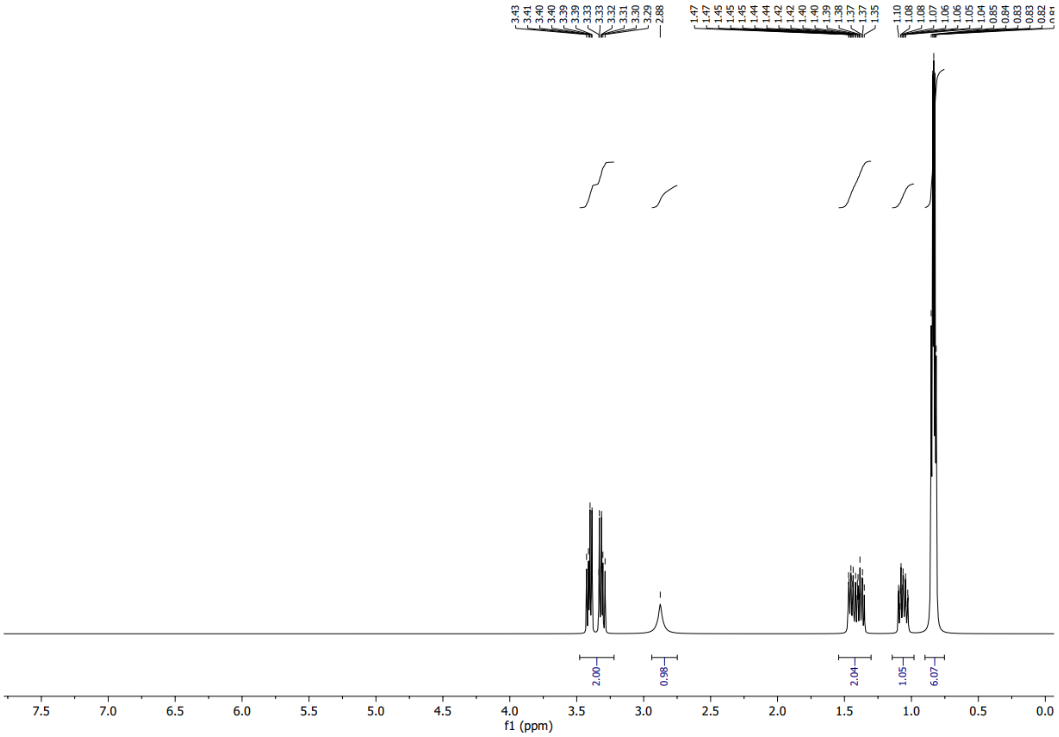

^{1}HNMR (400 MHz; $CDCl_3$) spectrum of compound **1l**.

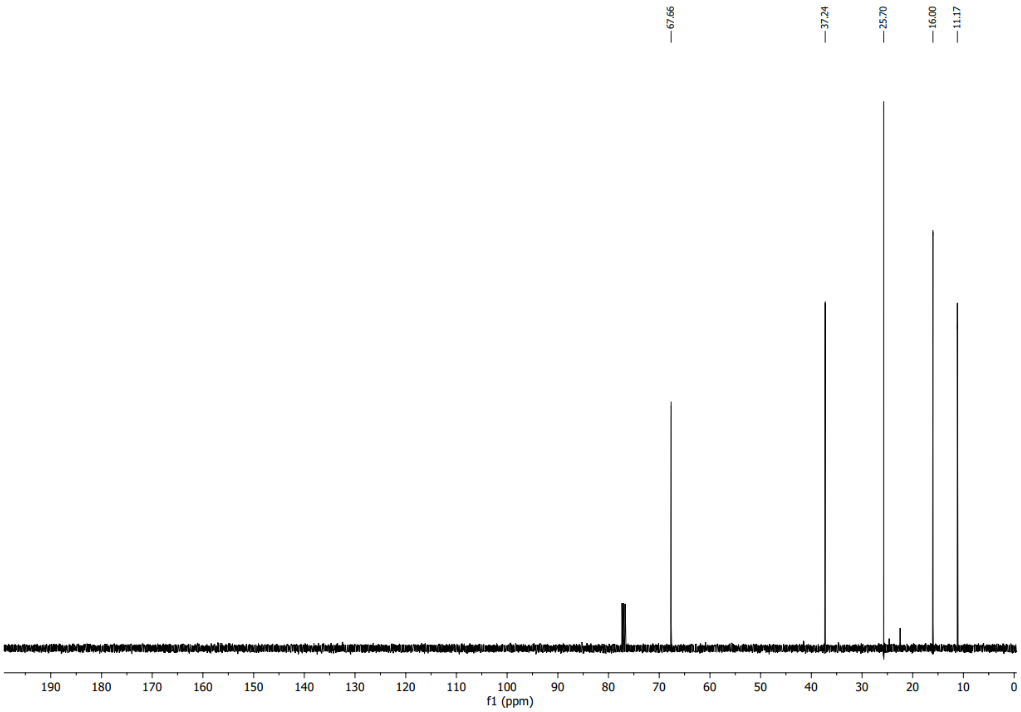

^{13}CNMR (100 MHz; $CDCl_3$) spectrum of compound **1l**.

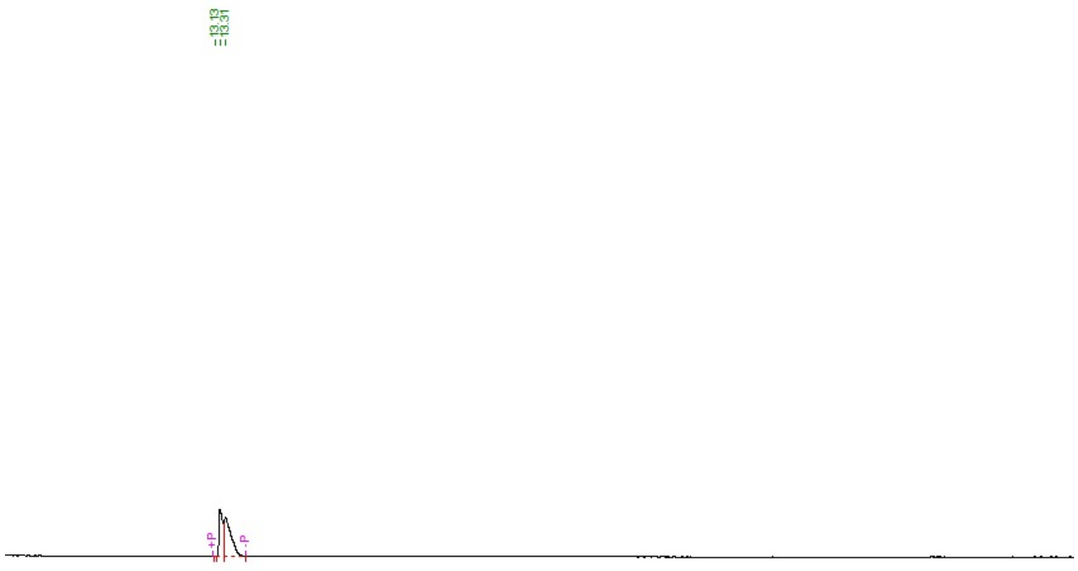

GC chromatogram (general GC method 2) of racemic compound **1l**.

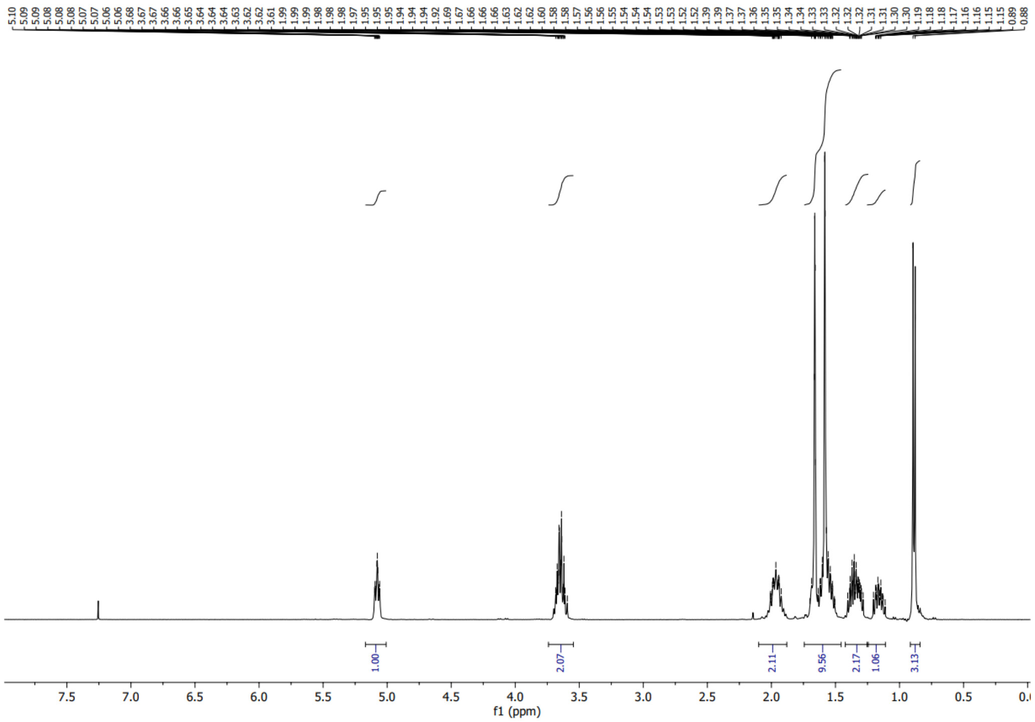

^{1}HNMR (400 MHz; $CDCl_3$) spectrum of compound **1m**.

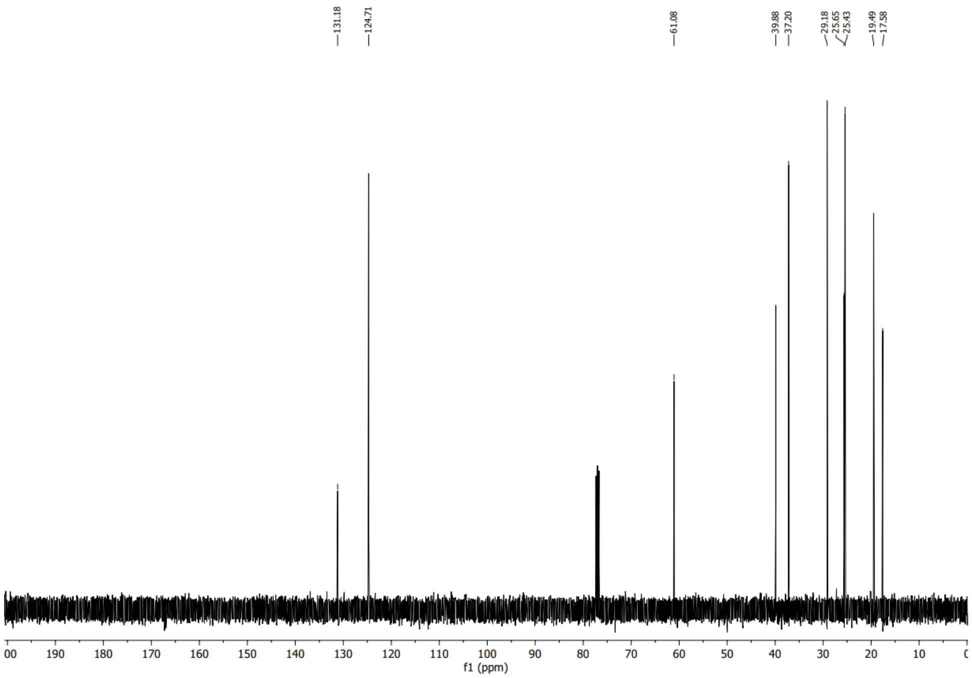

^{13}CNMR (100 MHz; $CDCl_3$) spectrum of compound **1m**.

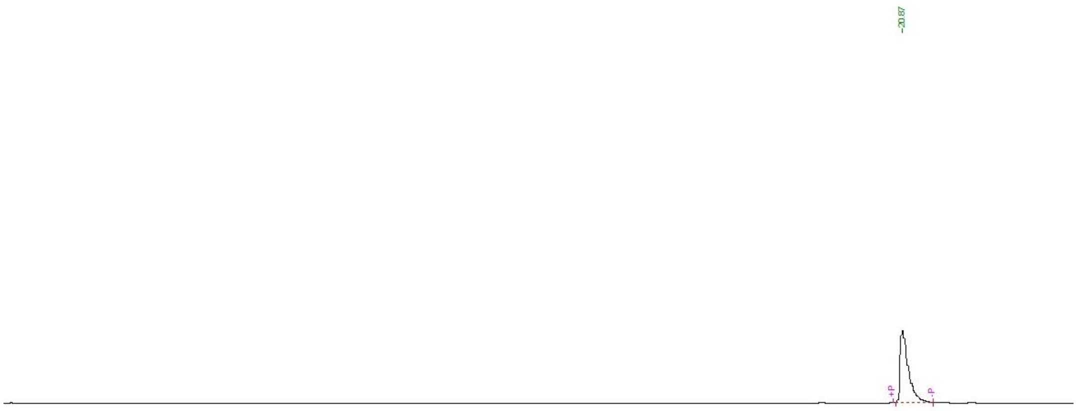

GC chromatogram (general GC method 1) of racemic compound **1m**.

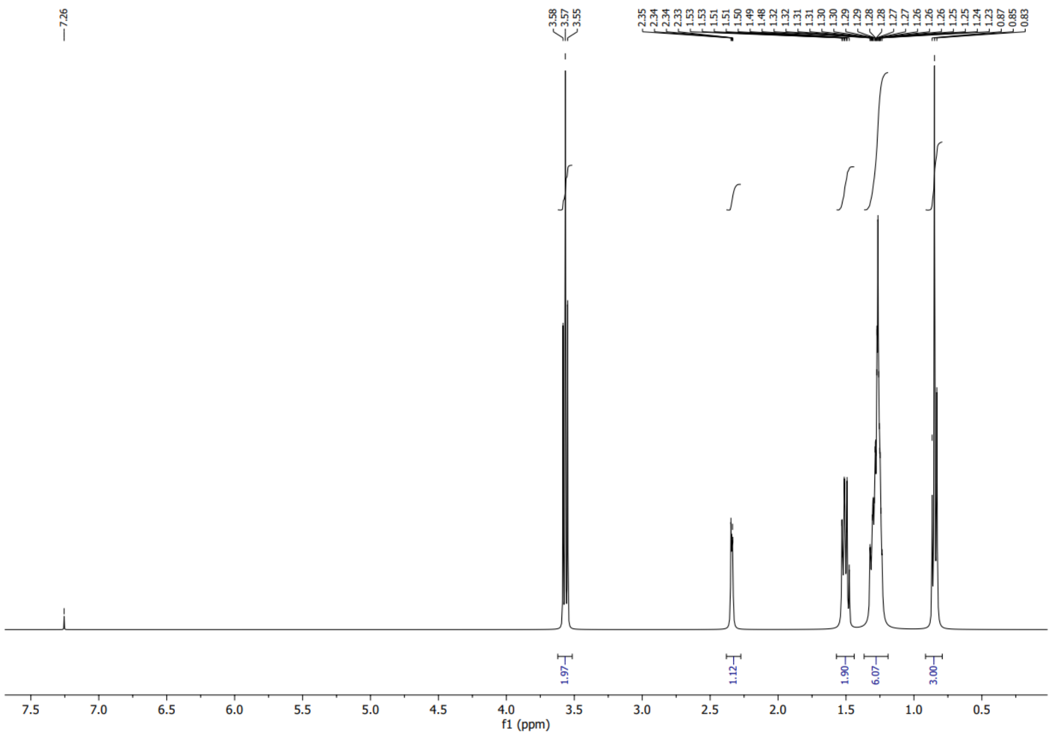

^{1}HNMR (400 MHz; $CDCl_3$) spectrum of compound **1n**.

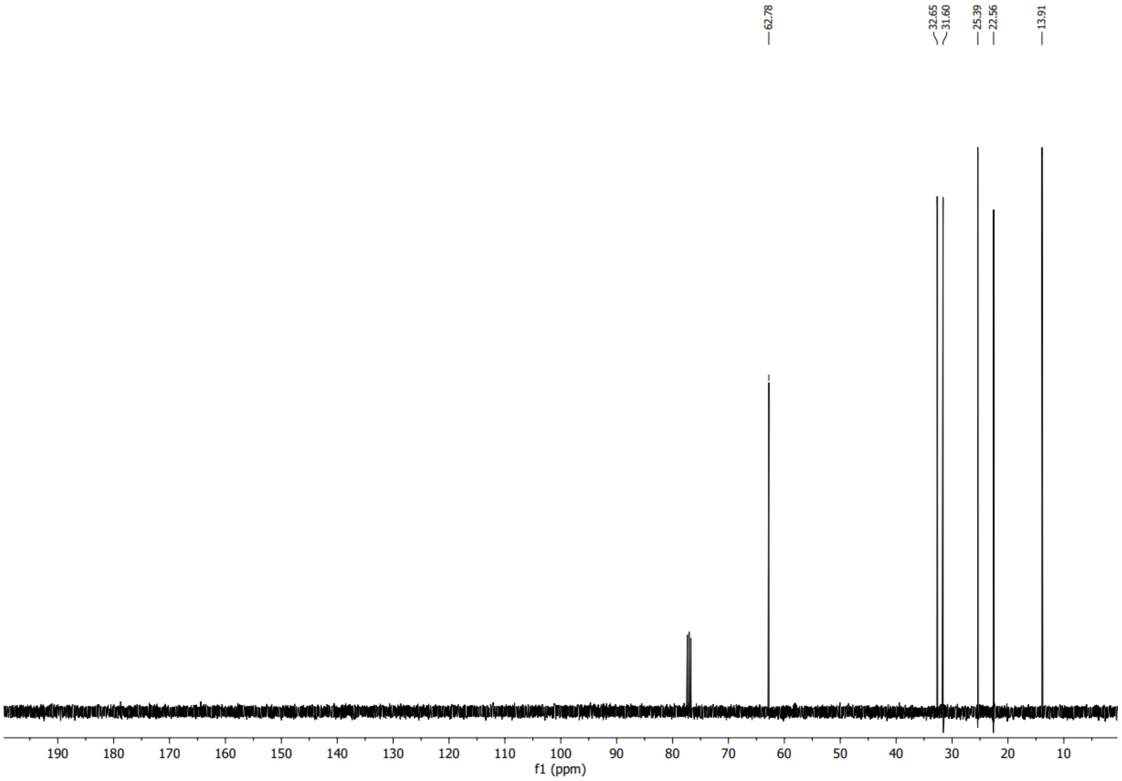

^{13}CNMR (100 MHz; $CDCl_3$) spectrum of compound **1n**.

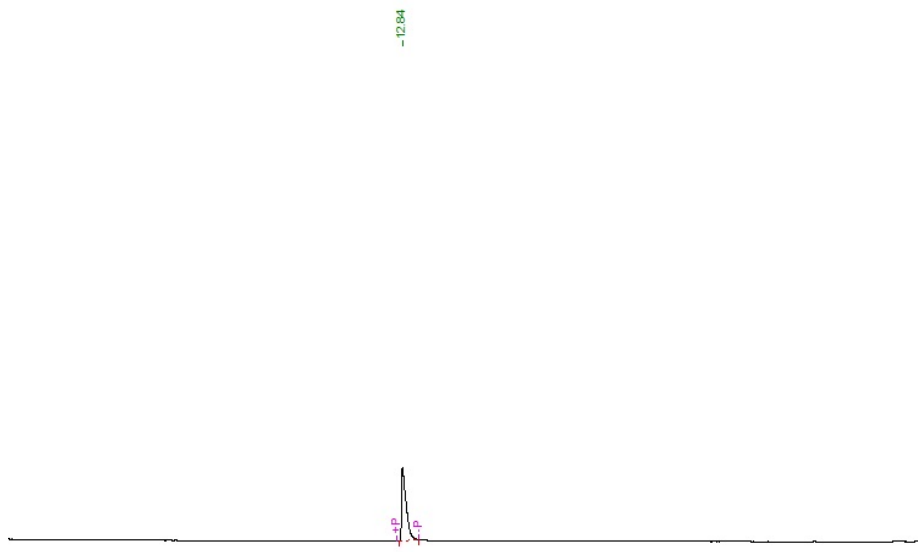

GC chromatogram (general GC method 1) of compound **1n**.

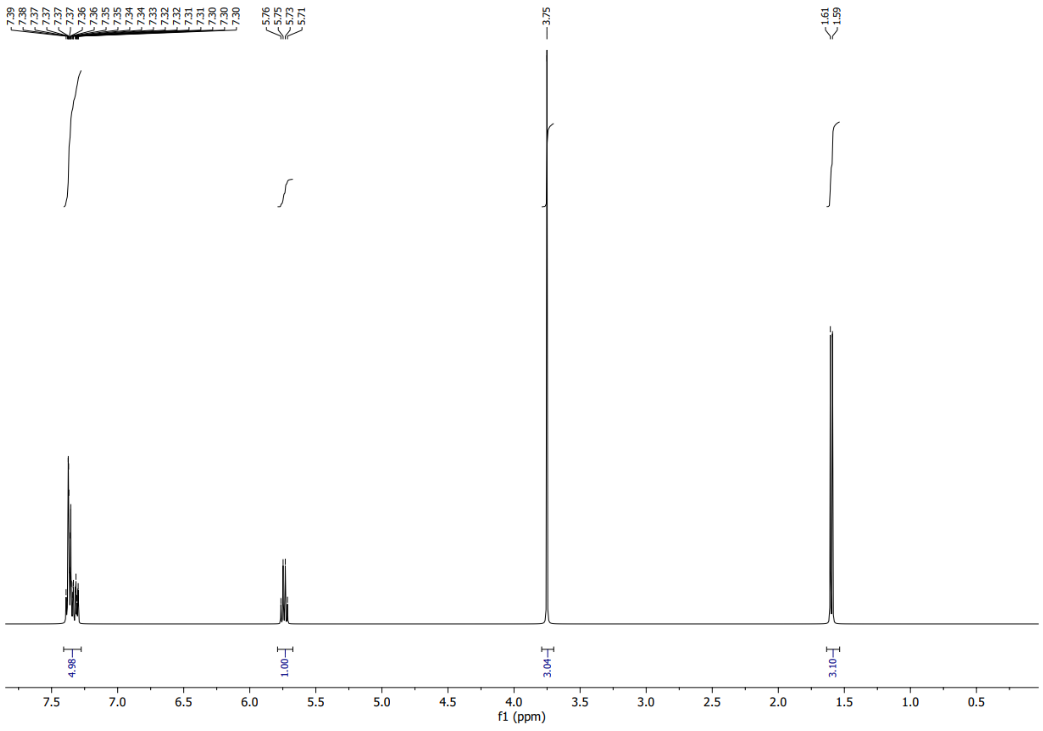

^{1}HNMR (400 MHz; $CDCl_3$) spectrum of compound **2a**.

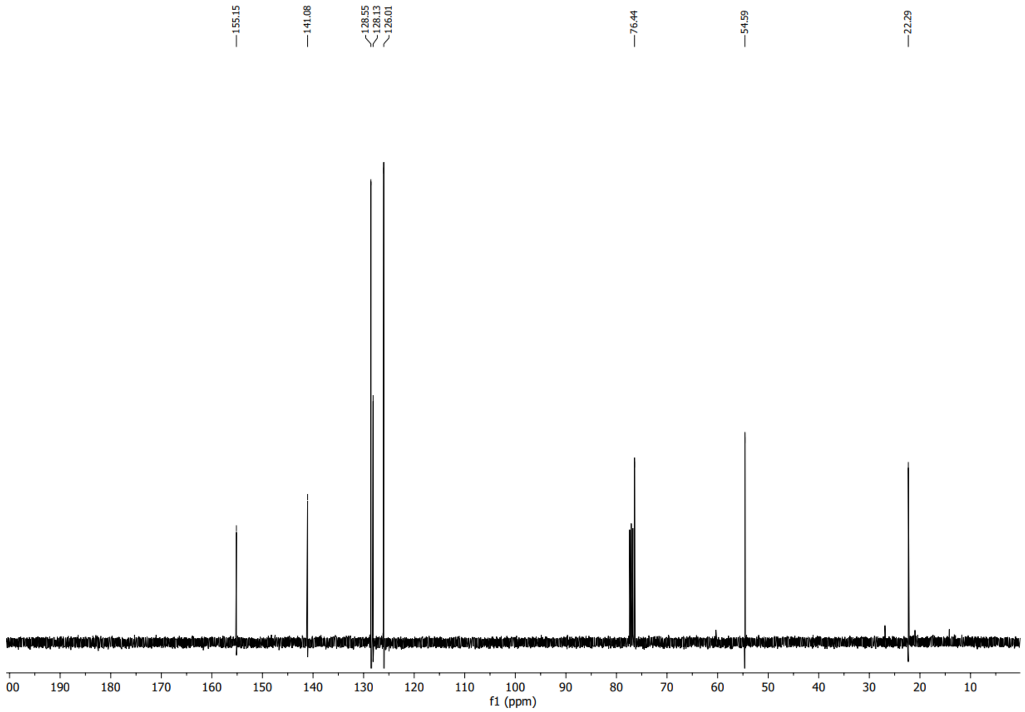

^{13}CNMR (100 MHz; $CDCl_3$) spectrum of compound **2a**.

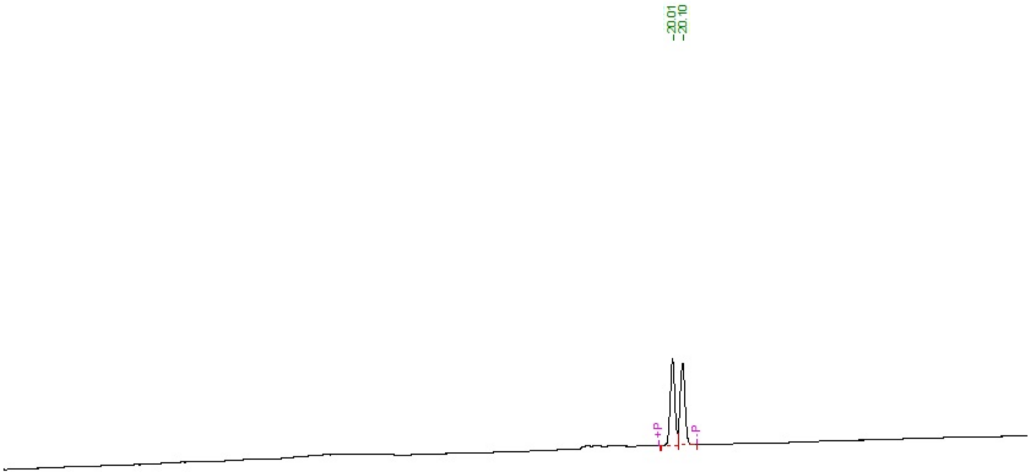

GC chromatogram (general GC method) of racemic compound **2a**.

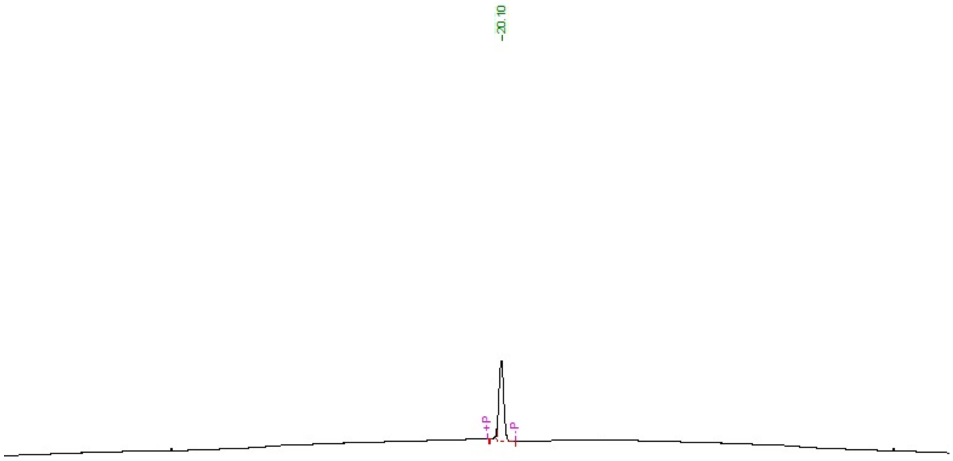

GC chromatogram (general GC method) of (*R*)—enantiomer of compound **2a**.

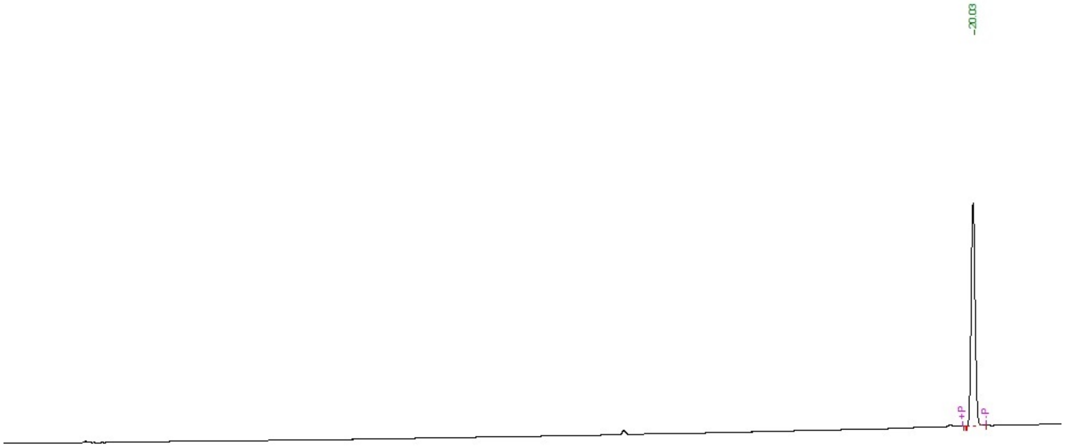

GC chromatogram (general GC method 1) of (*S*) – enantiomer of compound **2a**.

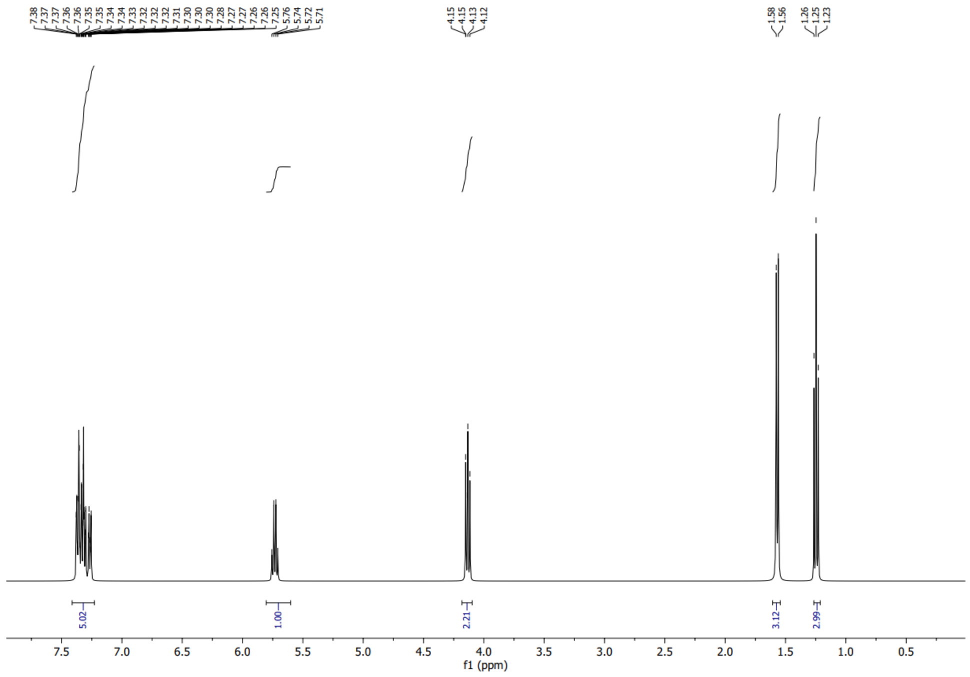

^{1}HNMR (400 MHz; $CDCl_3$) spectrum of compound **3a**.

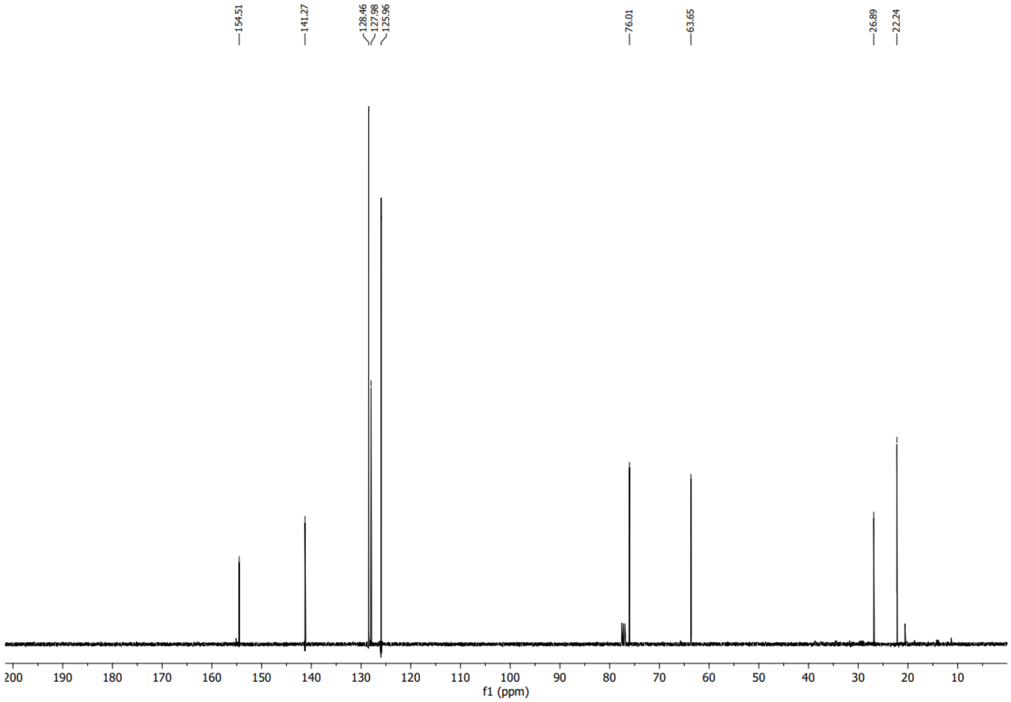

^{13}CNMR (100 MHz; $CDCl_3$) spectrum of compound **3a**.

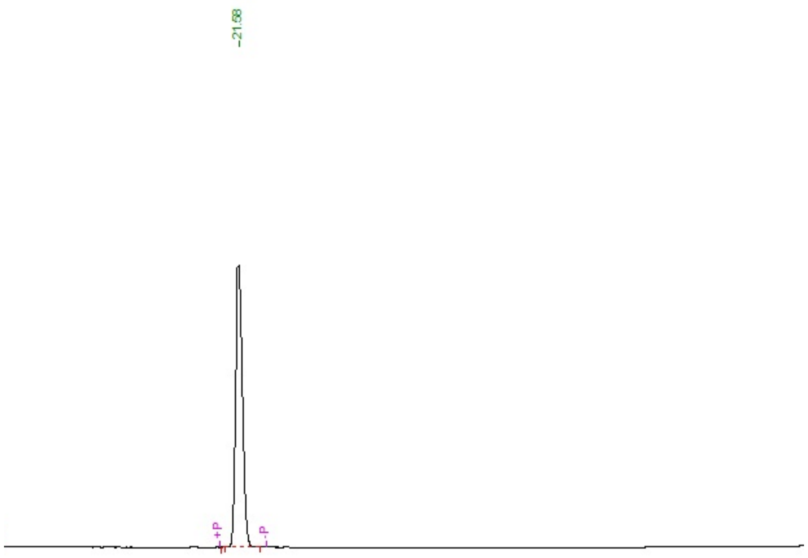

GC chromatogram (general GC method 1) of compound **3a**.

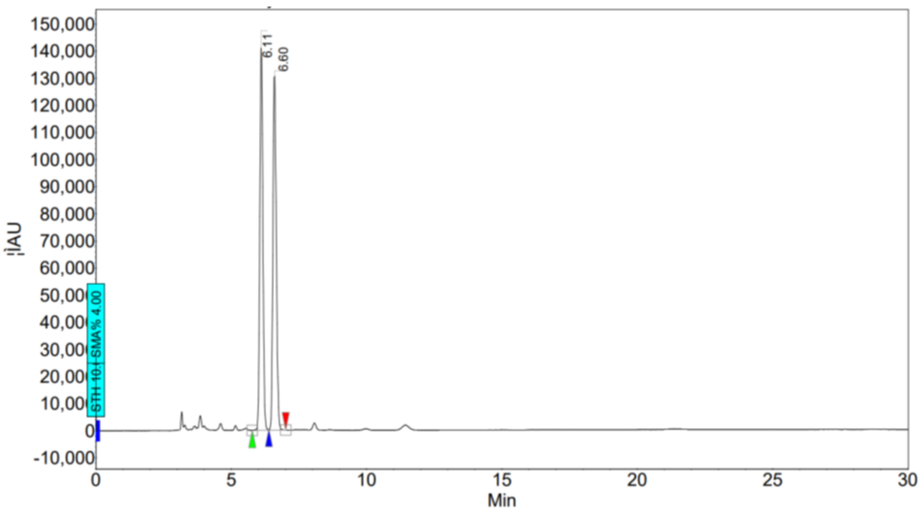

HPLC chromatogram (hexane:isopropanol : 98:2; column: OM; T=15 °C) of racemic compound **3a**.

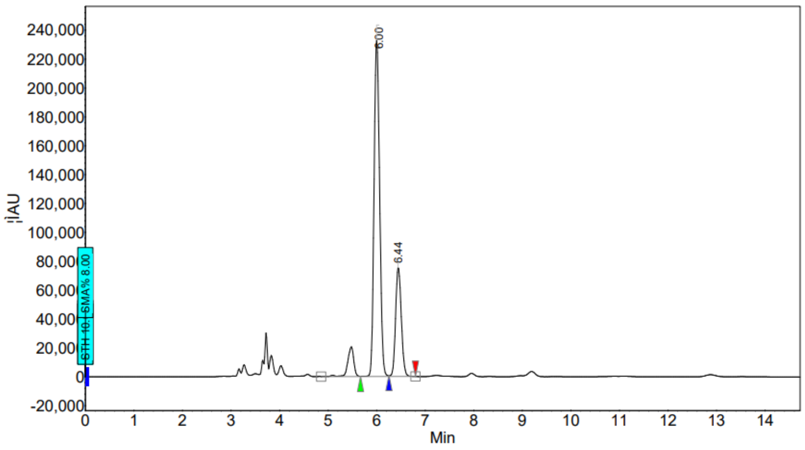

HPLC chromatogram (hexane:isopropanol : 98:2; column: OM; T=15 °C) of (*S*)-optically enriched (50 % *ee*) of compound **3a**.

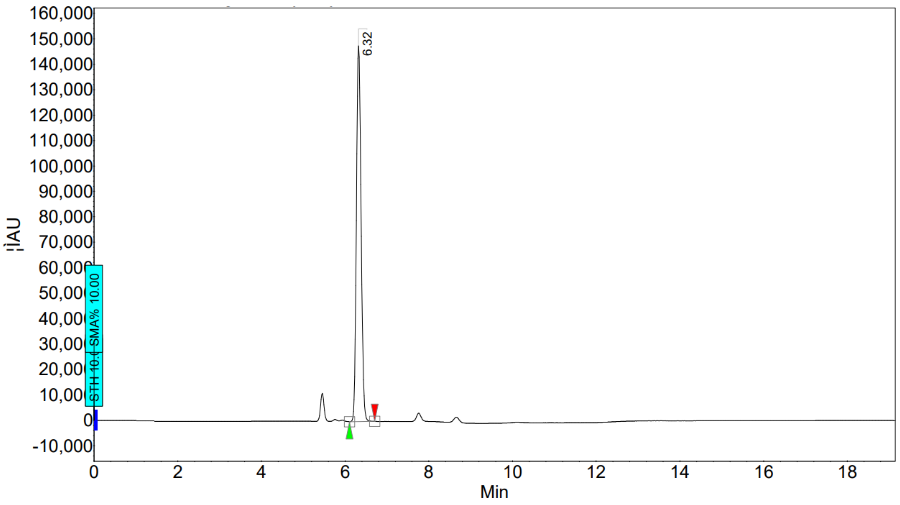

HPLC chromatogram (hexane:isopropanol : 98:2; column: OM; T=15 °C) of (*R*)-enantiomer

of compound **3a**.

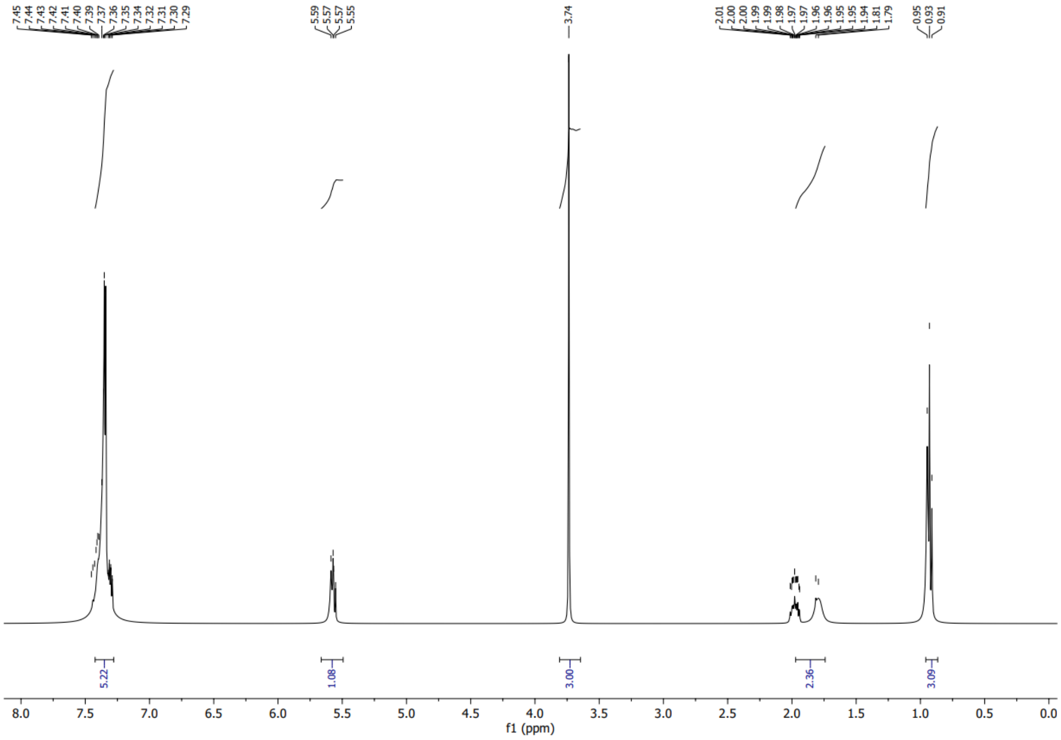

^{1}HNMR (400 MHz; $CDCl_3$) spectrum of compound **2b**.

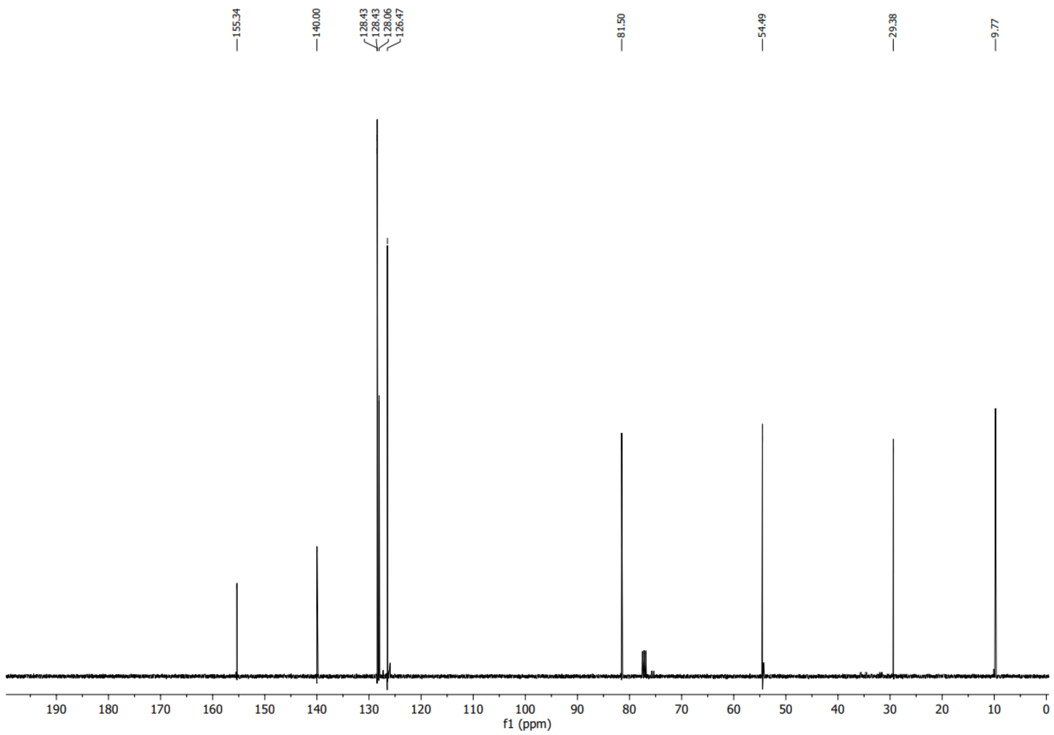

^{13}CNMR (100 MHz; $CDCl_3$) spectrum of compound **2b**.

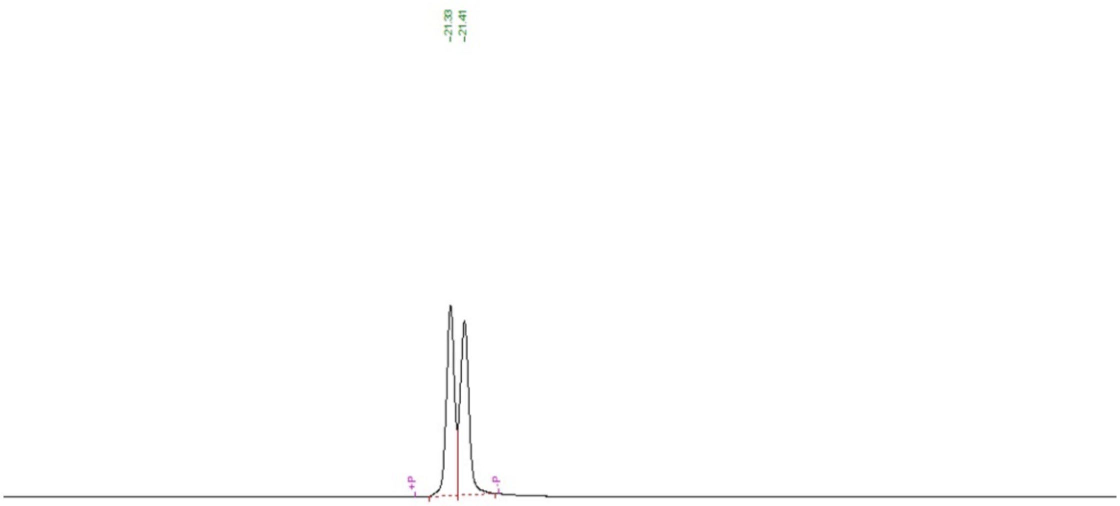

GC chromatogram (general GC method 1) of racemic **2b**.

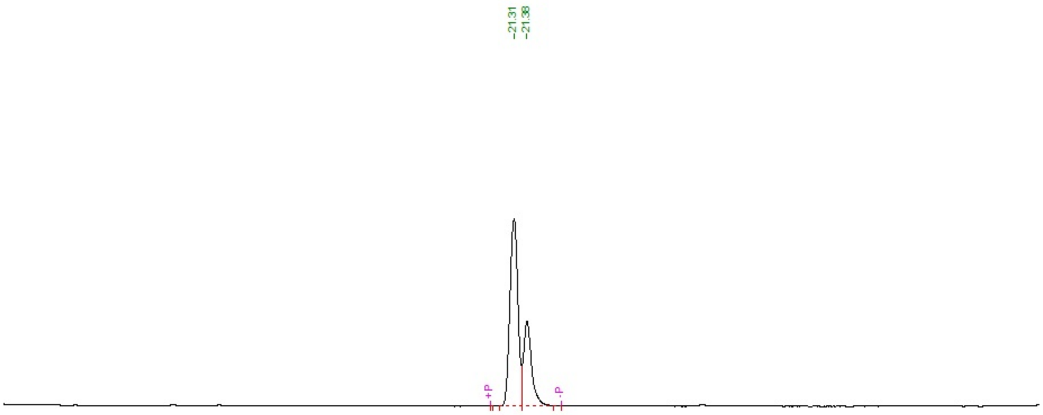

GC chromatogram (general GC method 1) of (*S*)-enantiomerically enriched (49 % *ee*) compound **2b**.

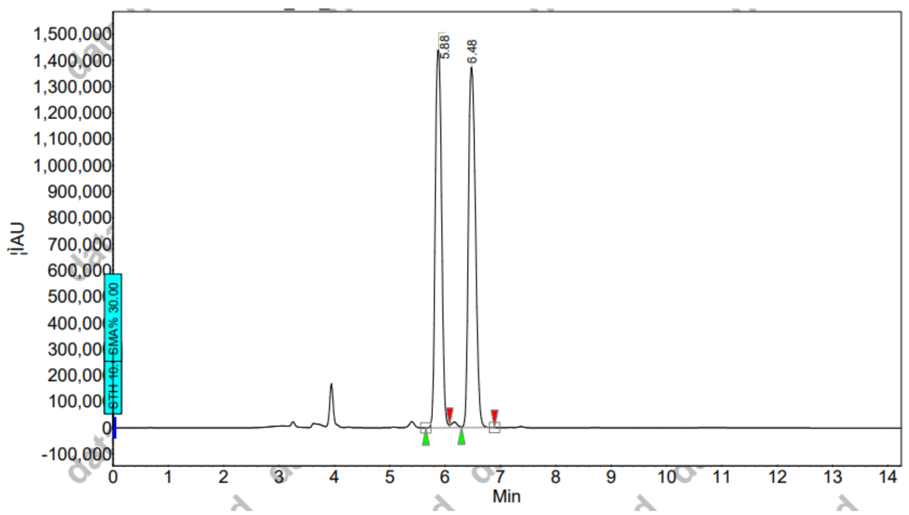

HPLC chromatogram (hexane:isopropanol : 98:2; column: OM) of racemic compound **2b**.

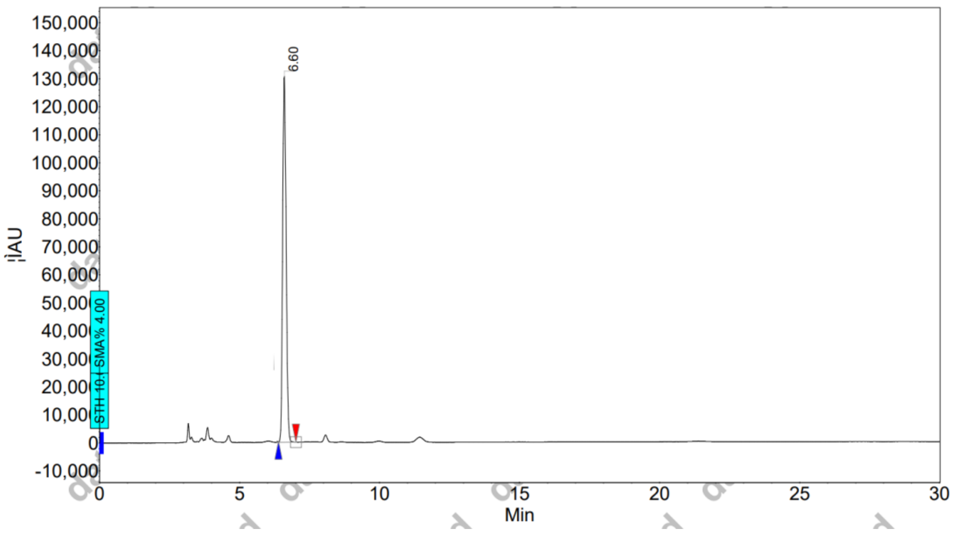

HPLC chromatogram (hexane:isopropanol : 98:2; column: OM) of (*R*)-enantiomer of compound **2b**.

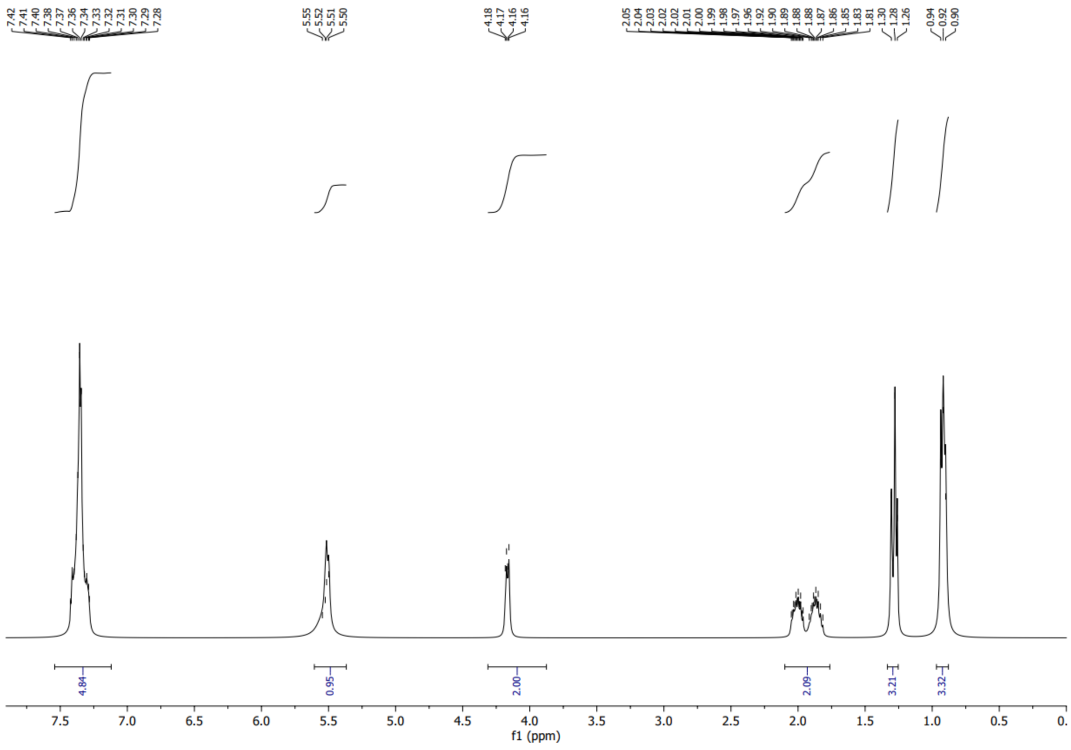

^{1}HNMR (400 MHz; $CDCl_3$) spectrum of compound **3b**.

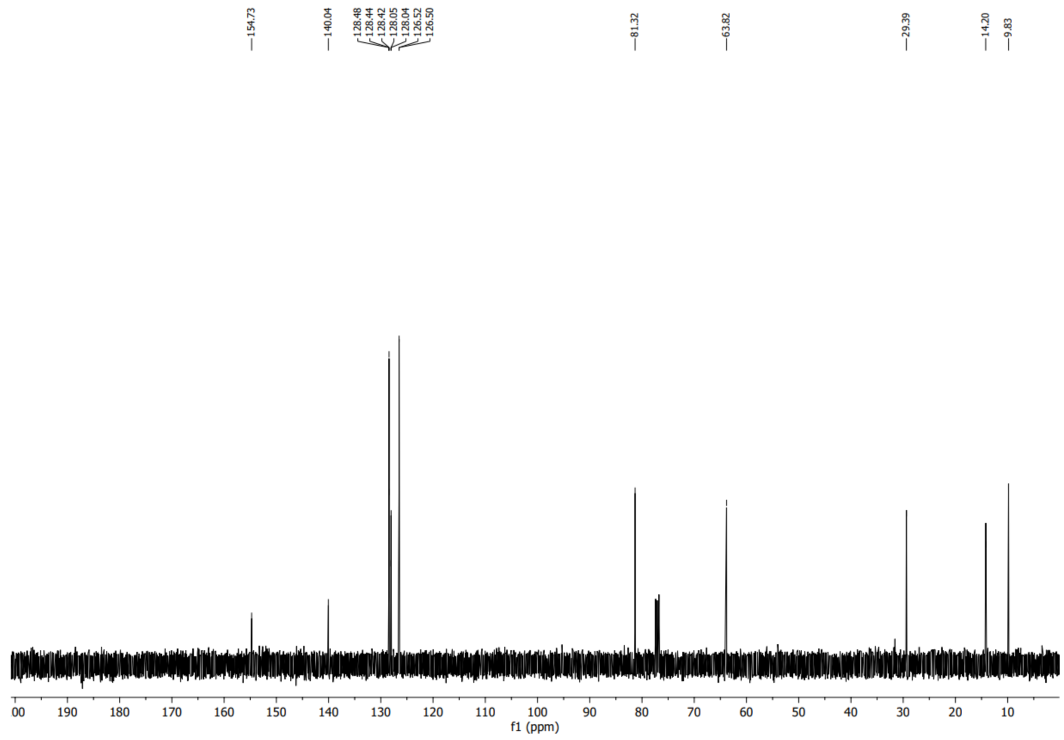

^{13}CNMR (100 MHz; $CDCl_3$) spectrum of compound **3b**.

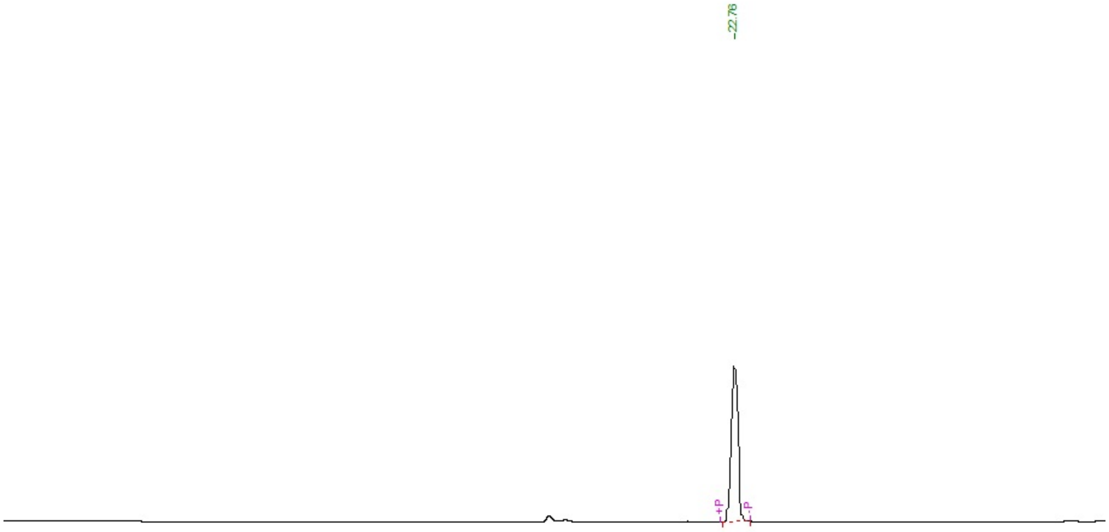

GC chromatogram (general GC method 1) of compound **3b**.

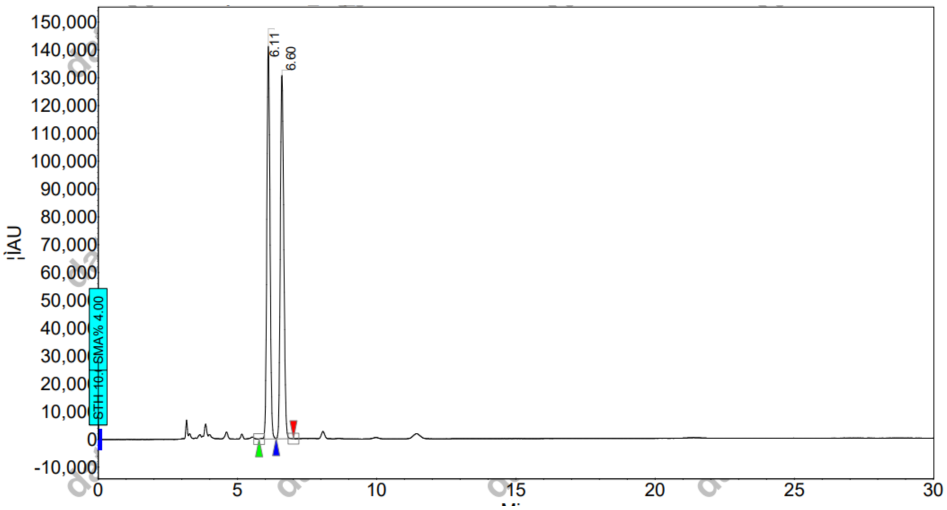

HPLC chromatogram (hexane:isopropanol : 98:2; column: OM; T=15 °C) of racemic compound **3b**.

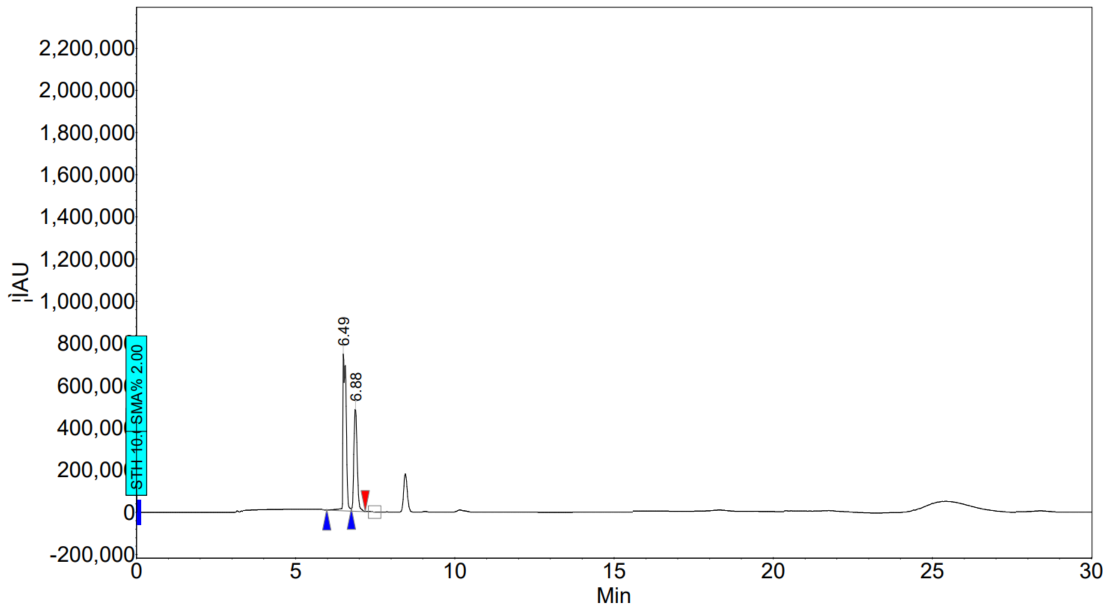

HPLC chromatogram (hexane:isopropanol : 98:2; column: OM; T=15 °C) of (*S*)-enantiomerically enriched (20 % *ee*) compound **3b**.

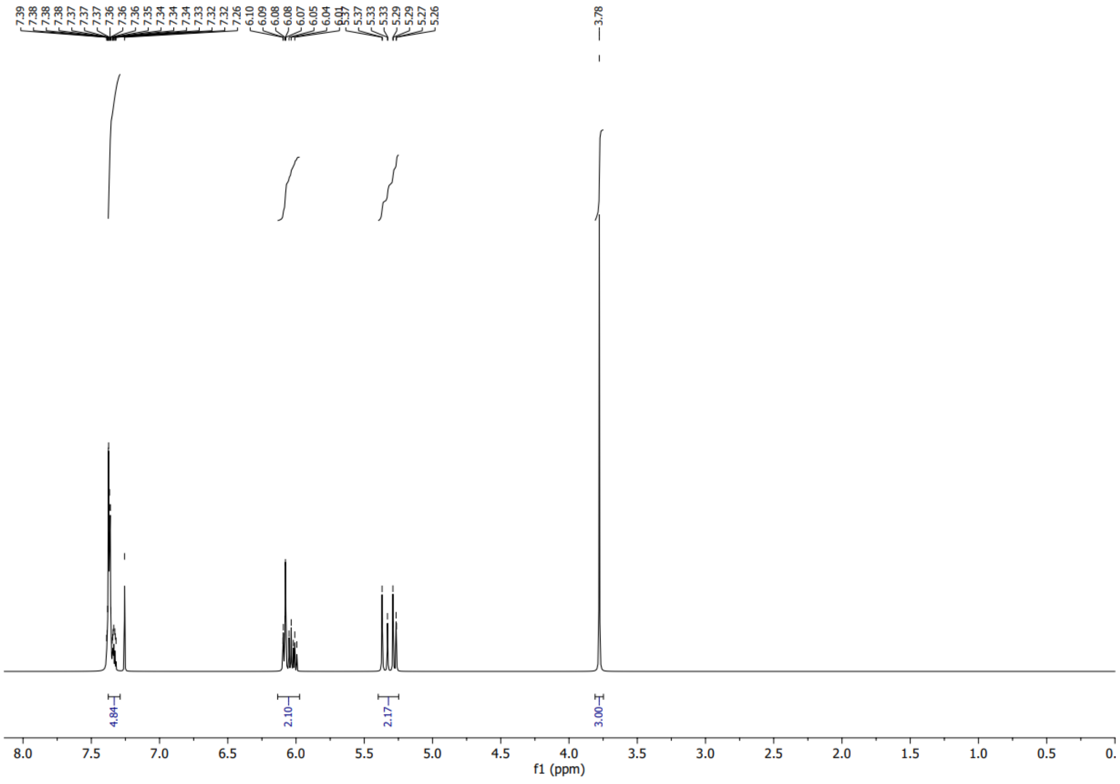

^{1}HNMR (400 MHz; $CDCl_3$) spectrum of compound **2c**.

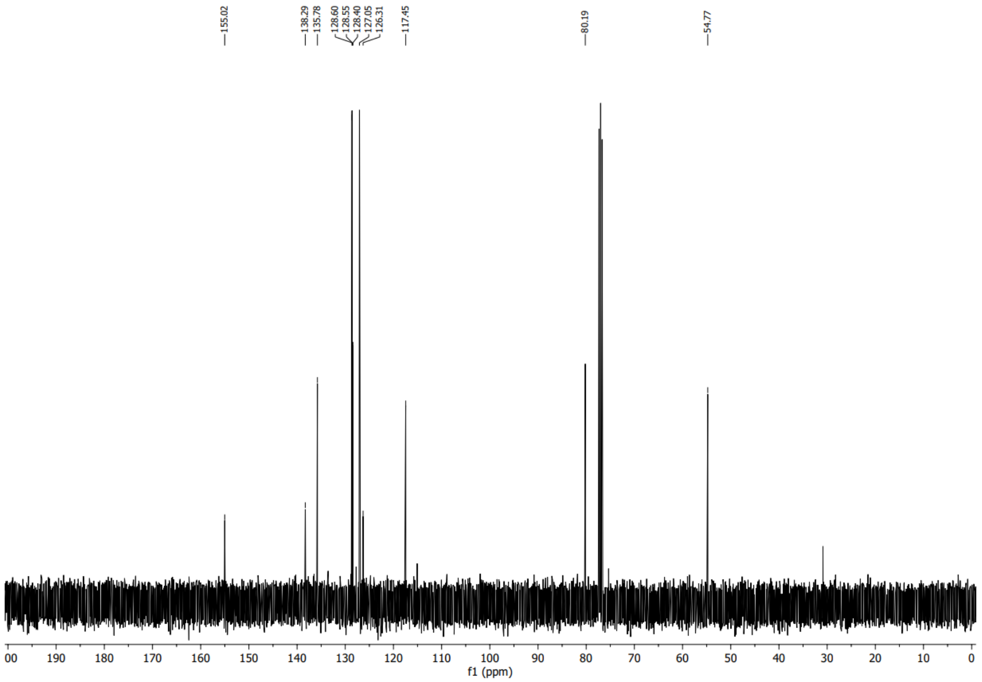

^{13}CNMR (100 MHz; $CDCl_3$) spectrum of compound **2c**.

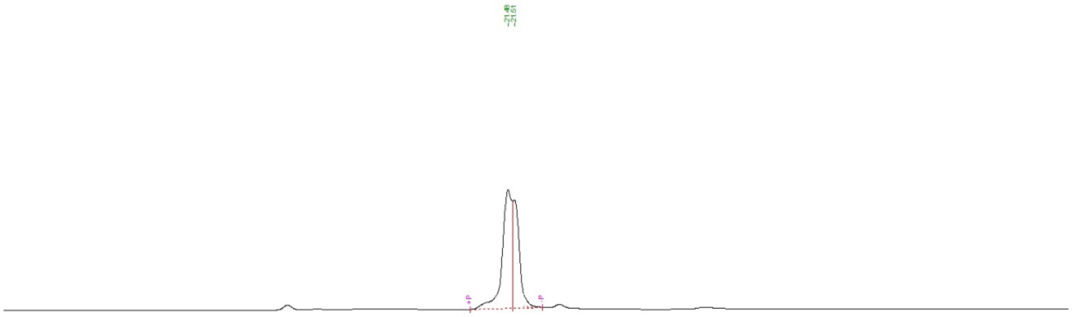

GC chromatogram (general GC method 1) of compound **2c**.

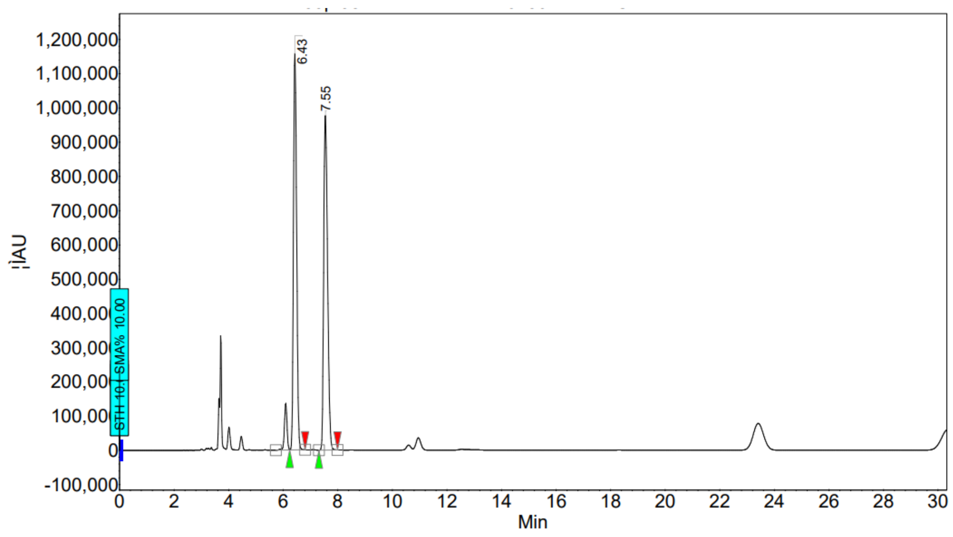

HPLC chromatogram (hexane:isopropanol 98:2, column OM; T = 15 °C) of racemic compound **2c** obtained according to general method.

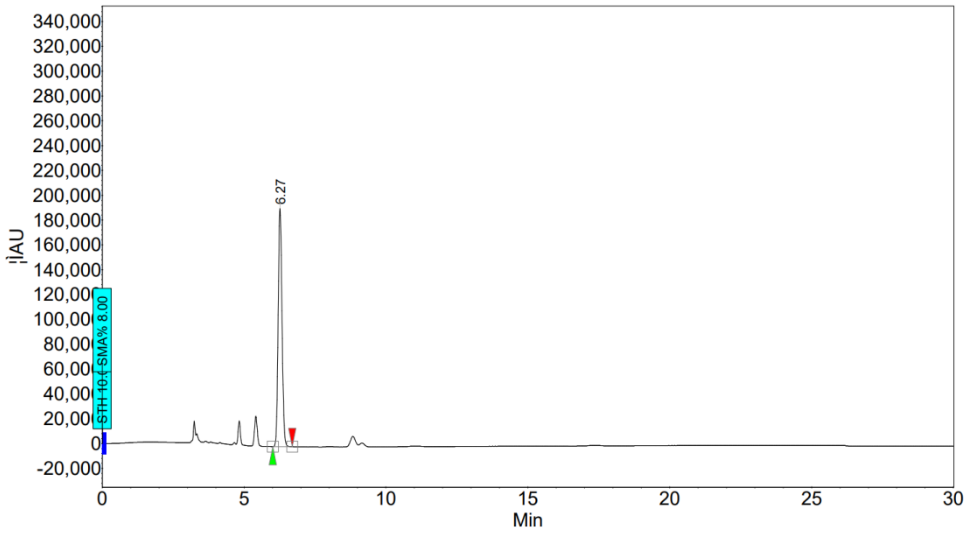

HPLC chromatogram (hexane:isopropanol 98:2, column OM; T = 15 °C) of (*R*)-enantiomer of compound **2c** obtained according to general method.

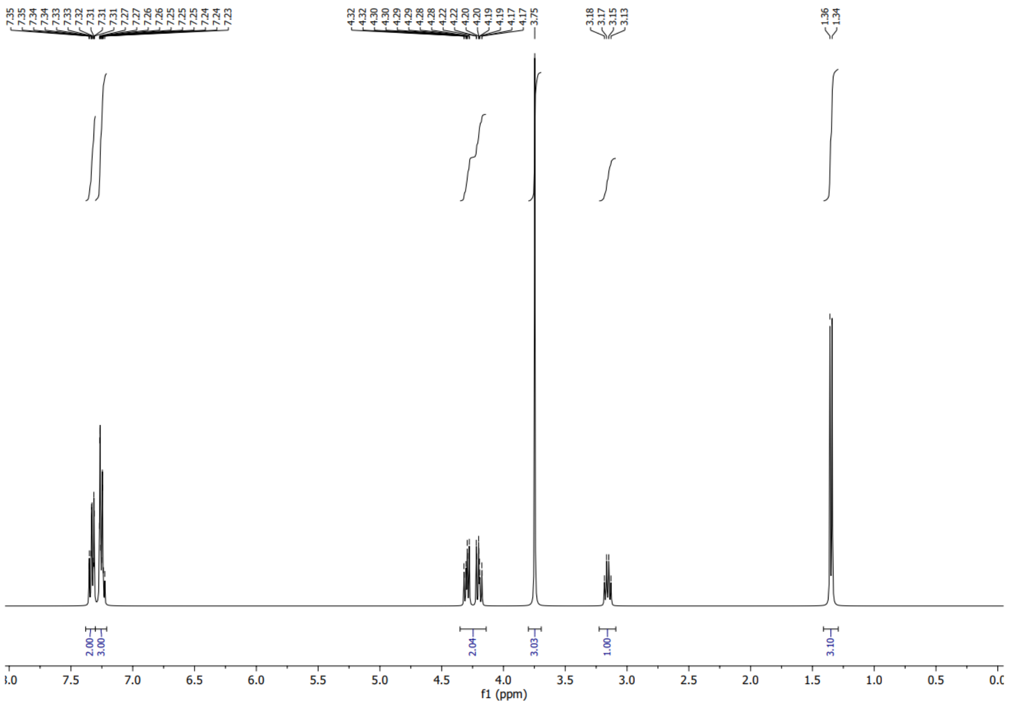

^{1}HNMR (400 MHz; $CDCl_3$) spectrum of compound **2d**.

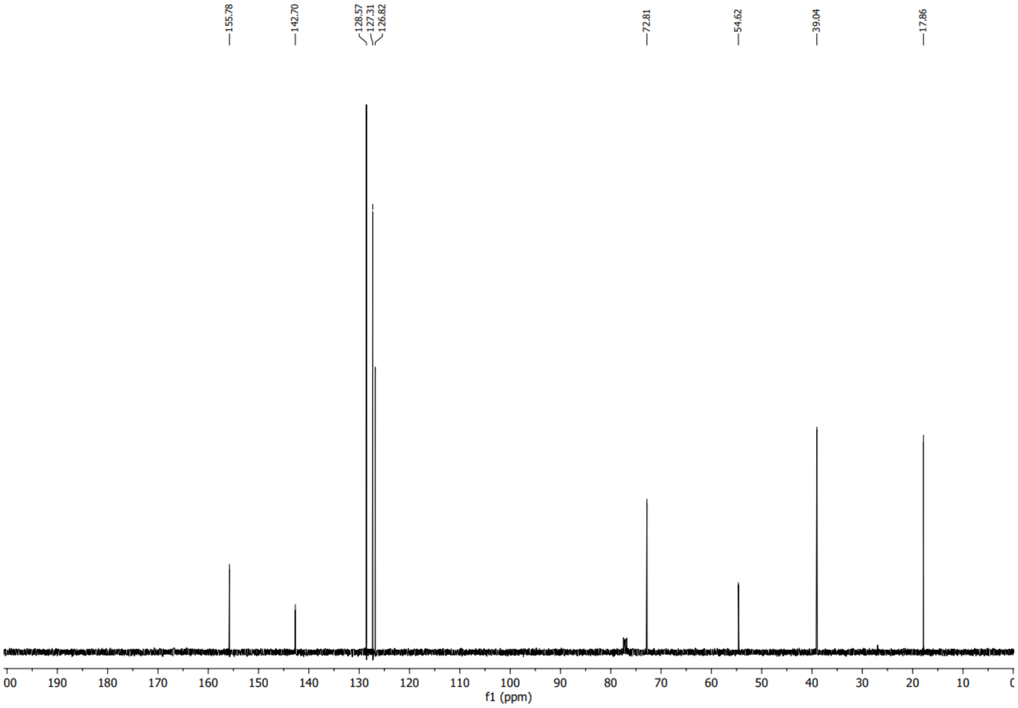

^{13}CNMR (100 MHz; $CDCl_3$) spectrum of compound **2d**.

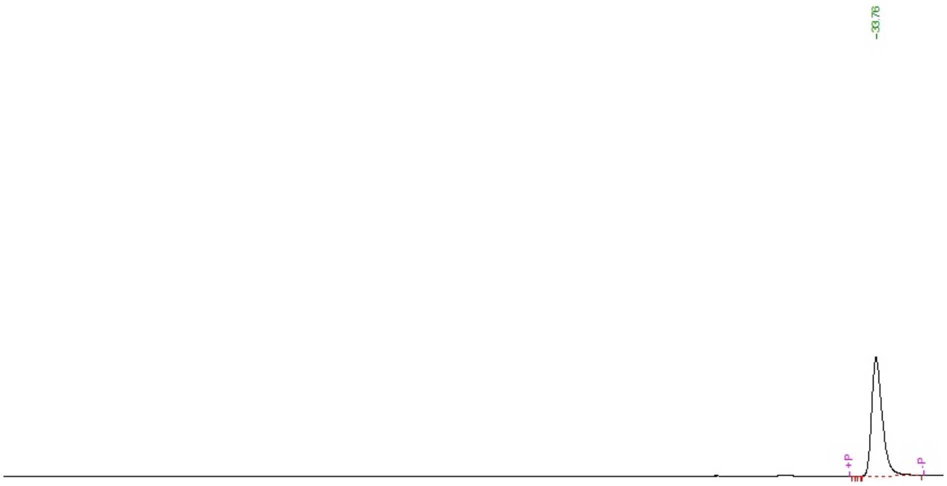

GC chromatogram (general GC method 2) of compound **2d**.

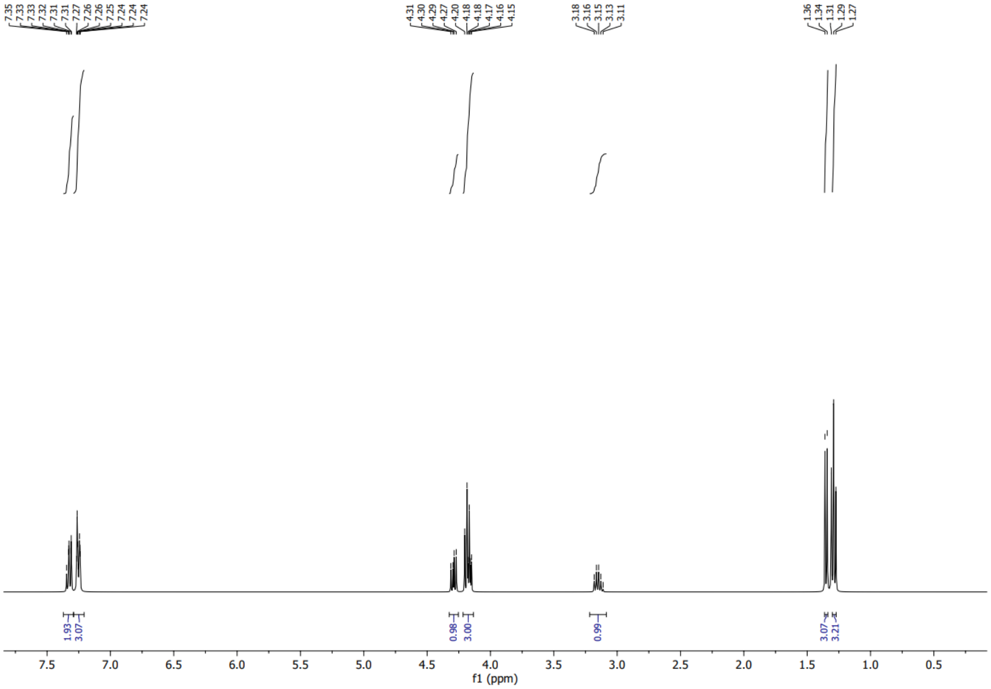

^{1}HNMR (400 MHz; $CDCl_3$) spectrum of compound **3d**.

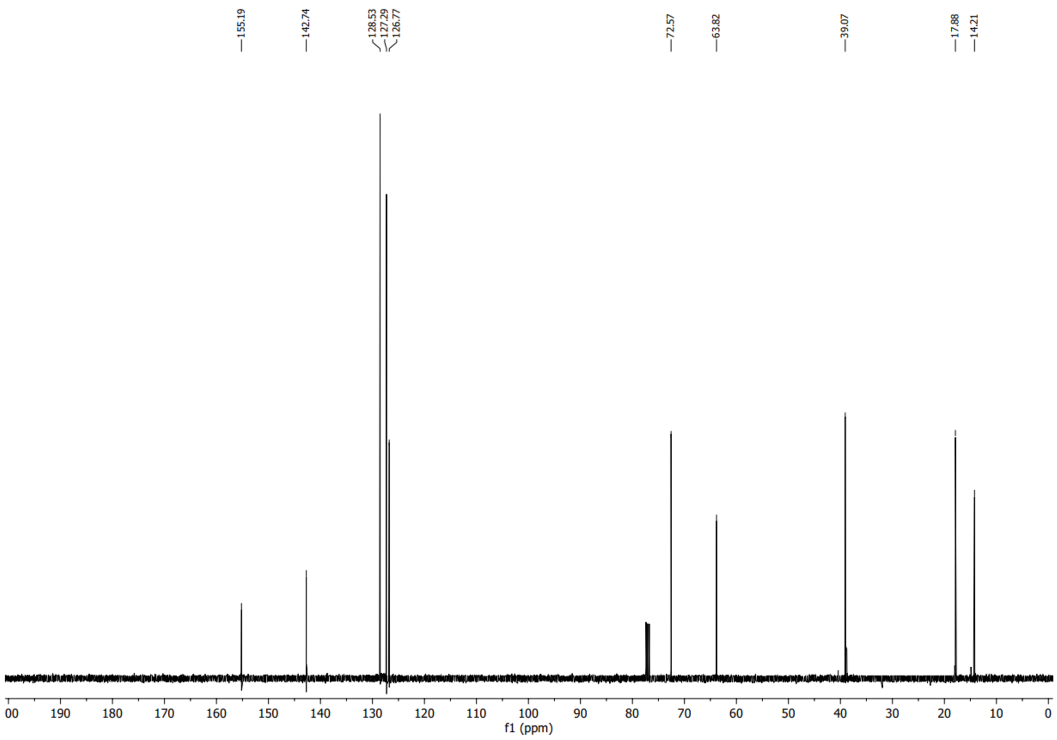

^{13}CNMR (100 MHz; $CDCl_3$) spectrum of compound **3d**.

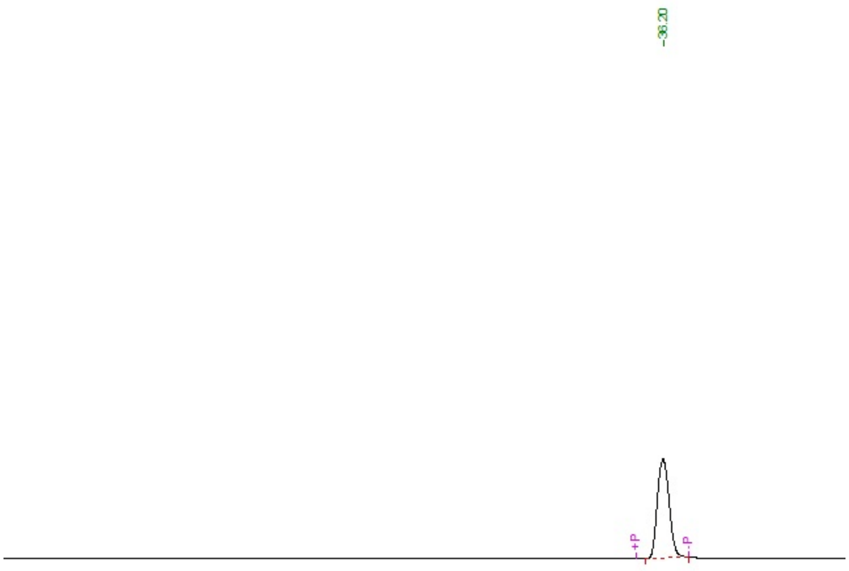

GC chromatogram (general GC method 2) of compound **3d**.

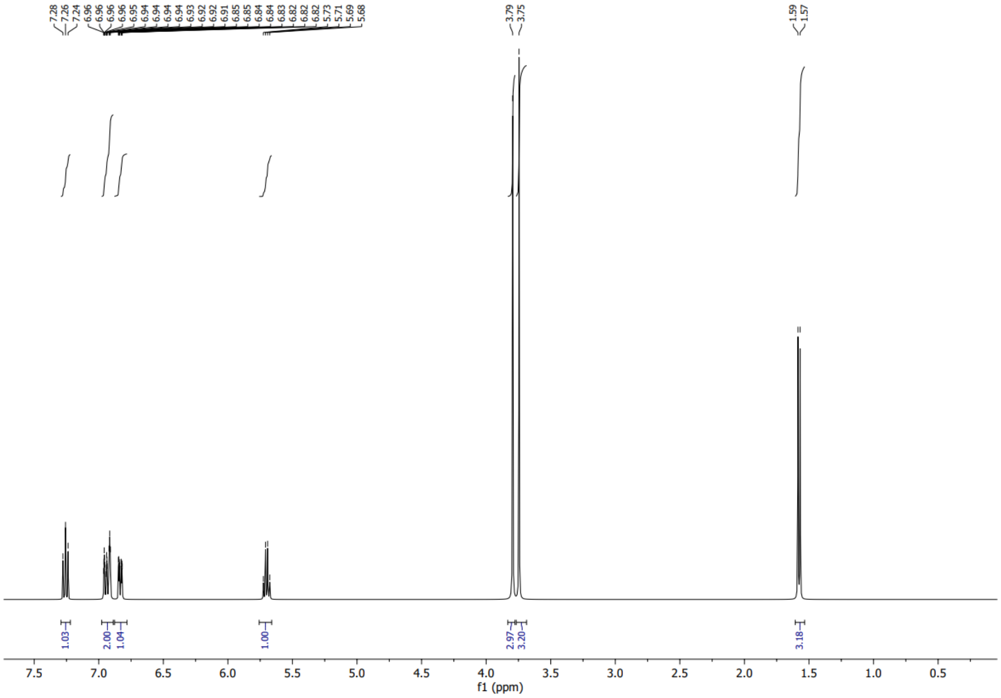

^{1}HNMR (400 MHz; $CDCl_3$) spectrum of compound **2e**.

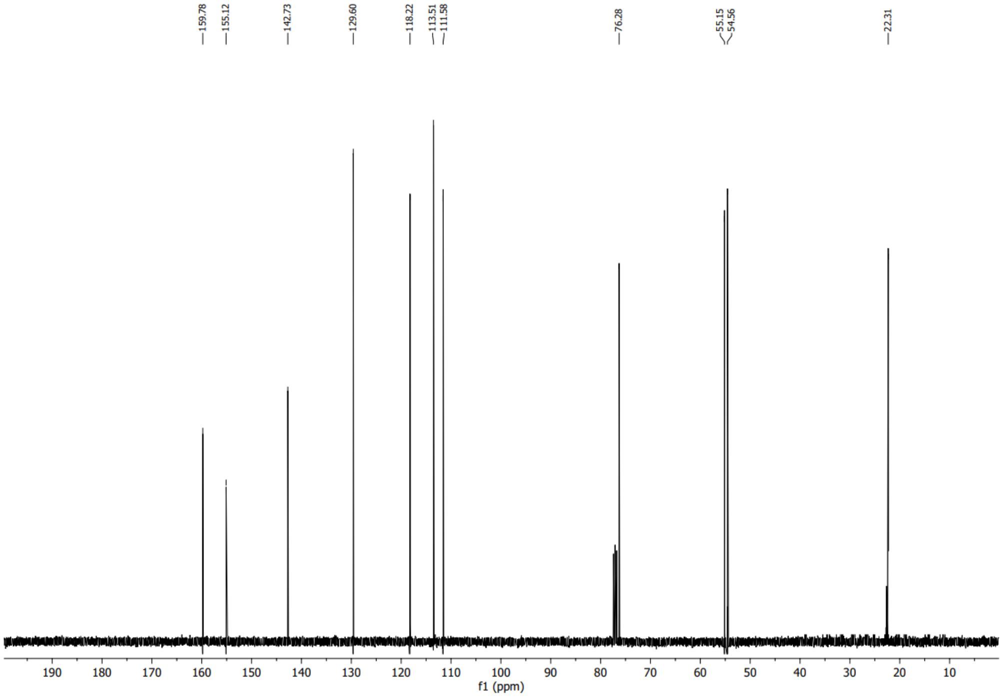

^{13}CNMR (100 MHz; $CDCl_3$) spectrum of compound **2e**.

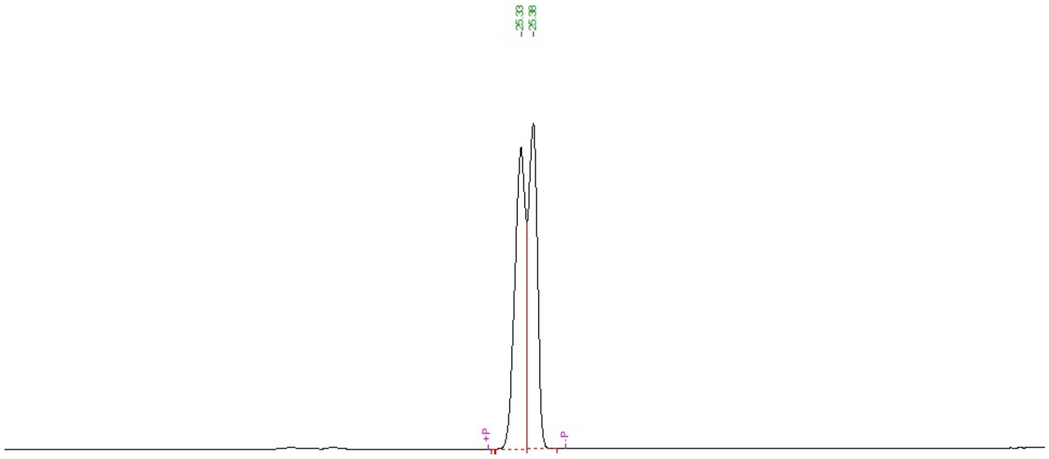

GC chromatogram (general GC method 1) of racemic compound **2e**.

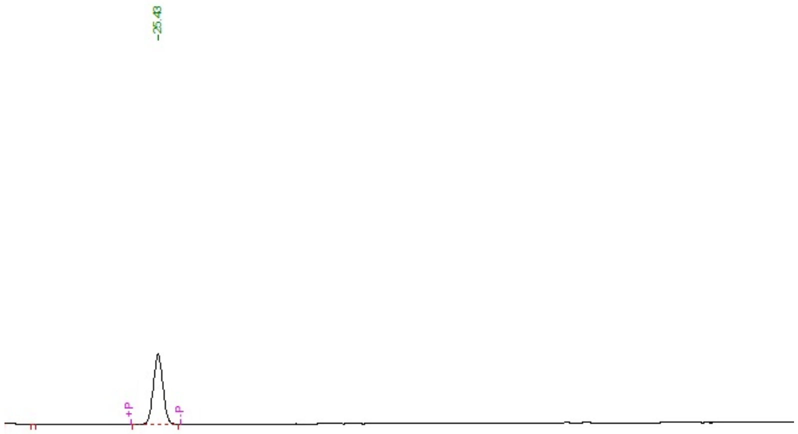

GC chromatogram (general GC method 1) of (*R*) enantiomer of compound **2e**.

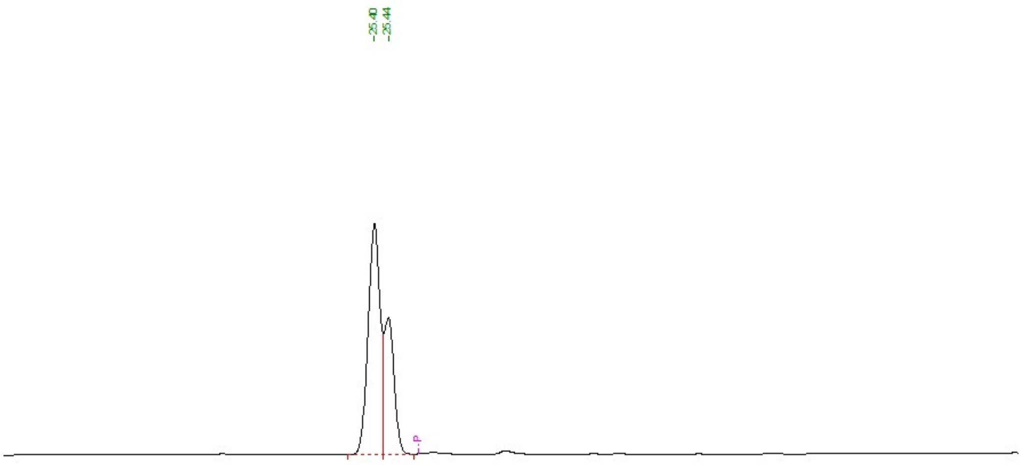

GC chromatogram (general GC method 1) of (*S*) optically enriched (36 % *ee*) of compound **2e**.

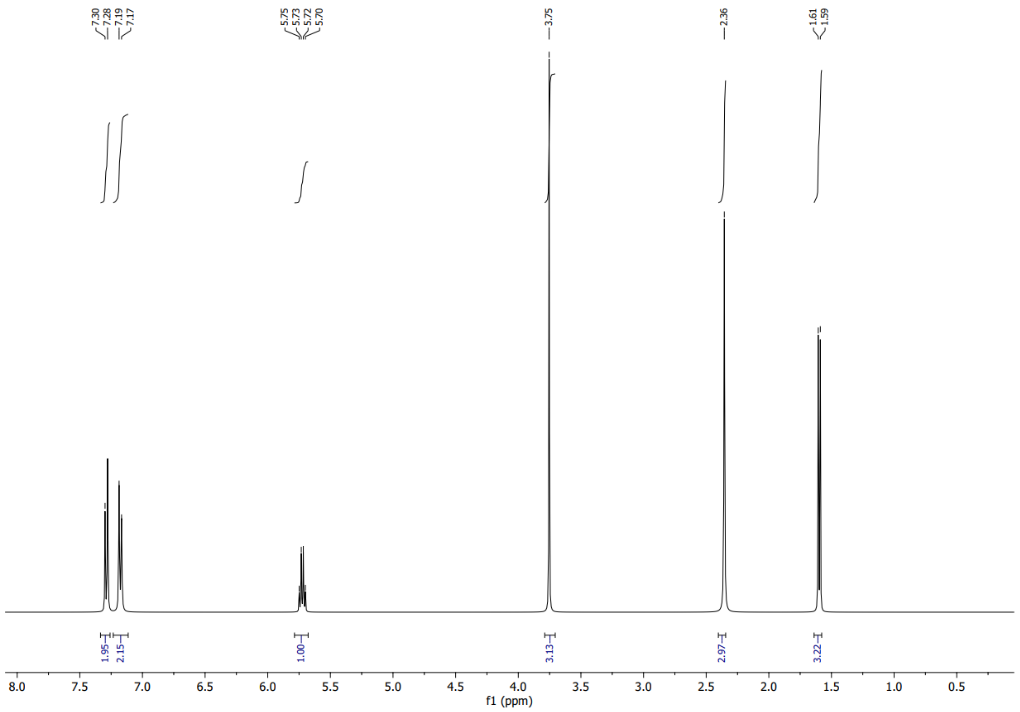

^{1}HNMR (400 MHz; $CDCl_3$) spectrum of compound **2f**.

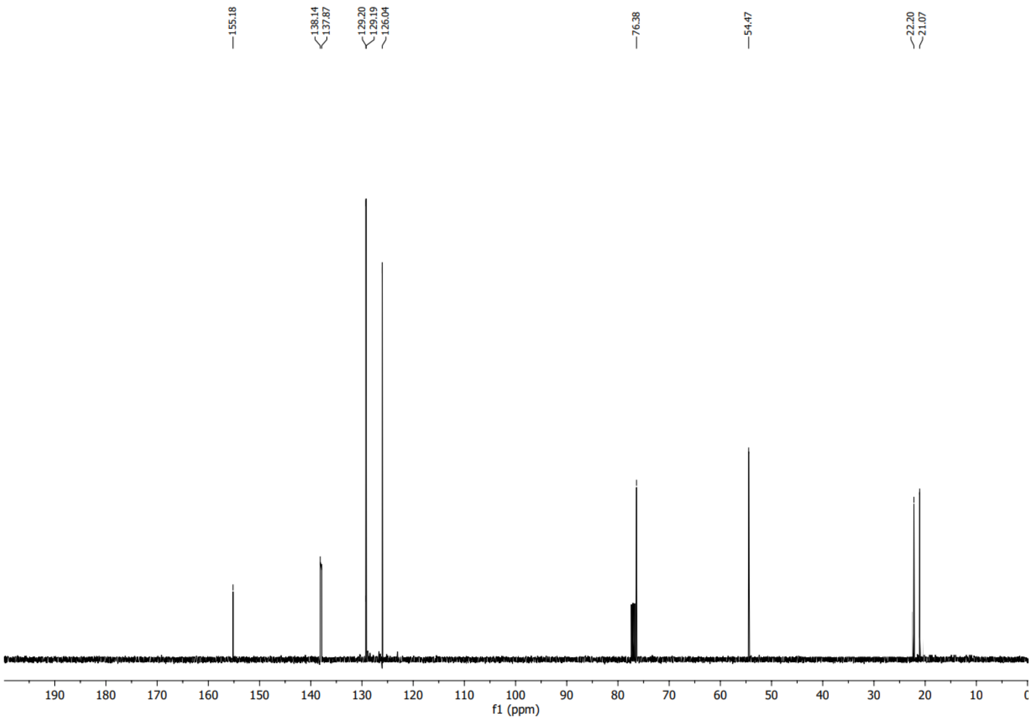

^{13}CNMR (100 MHz; $CDCl_3$) spectrum of compound **2f**.

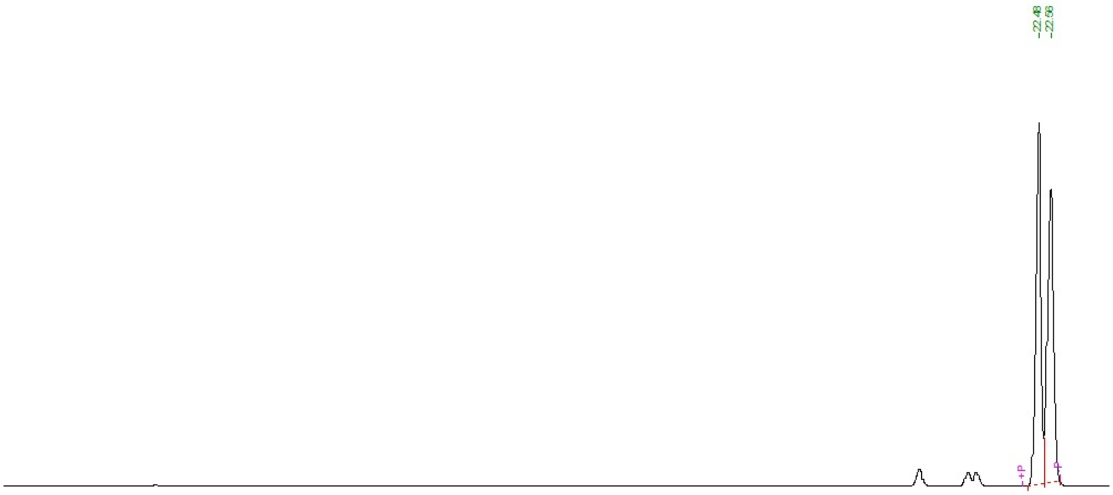

GC chromatogram (general GC method 1) of compound **2f**.

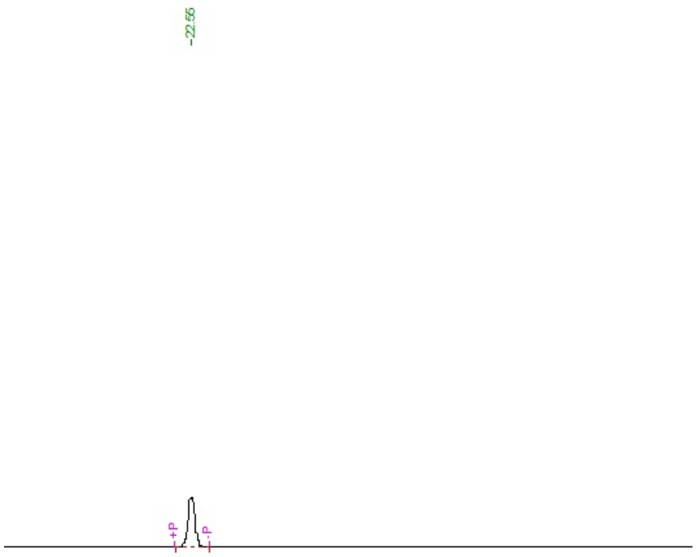

GC chromatogram (general GC method 1) of (*R*) – enantiomer of compound **2f**.

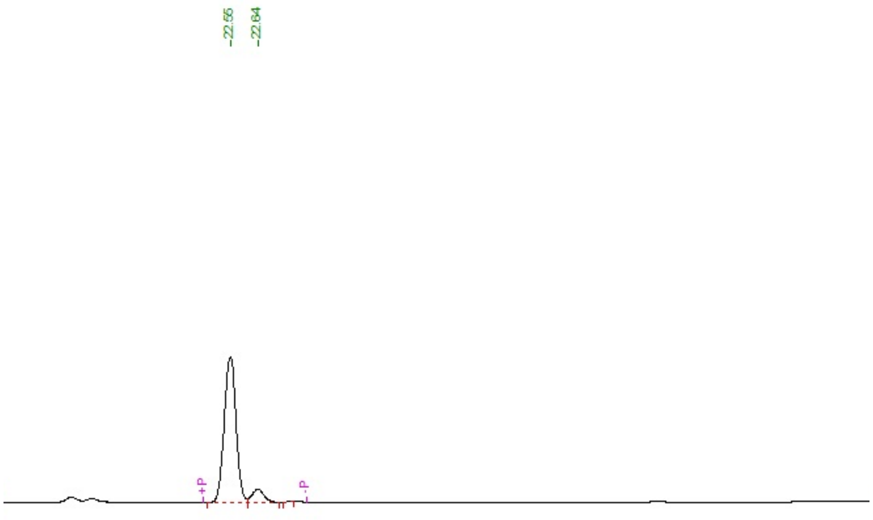

GC chromatogram (general GC method 1) of (*S*) – enantiomerically enriched (83 % *ee*) compound **2f**.

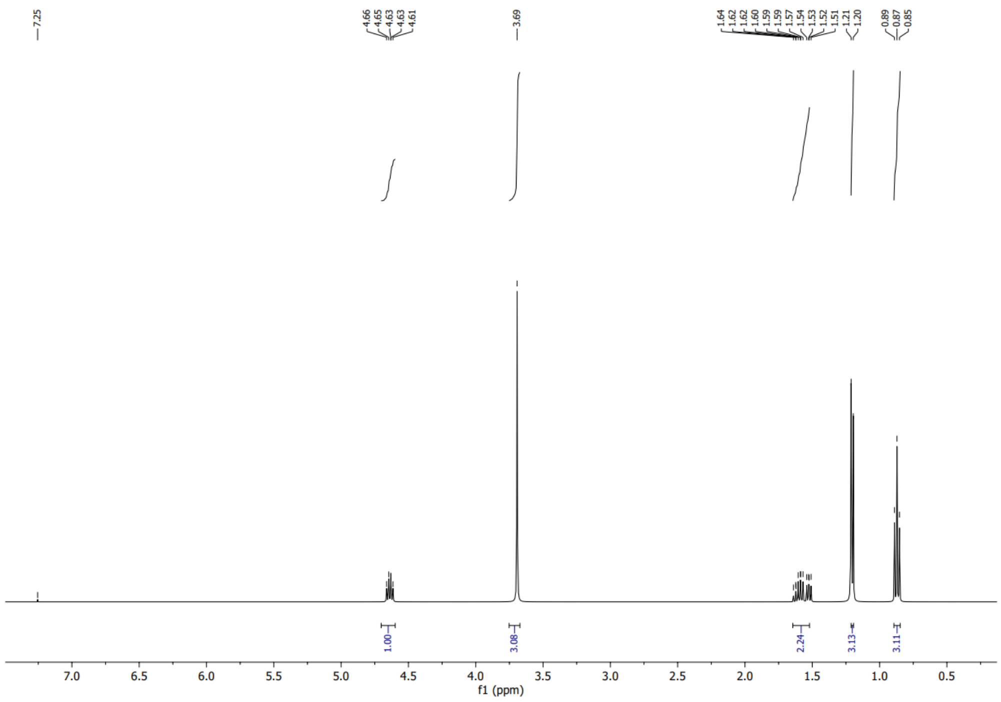

^{1}HNMR (400 MHz; $CDCl_3$) spectrum of compound **2g**.

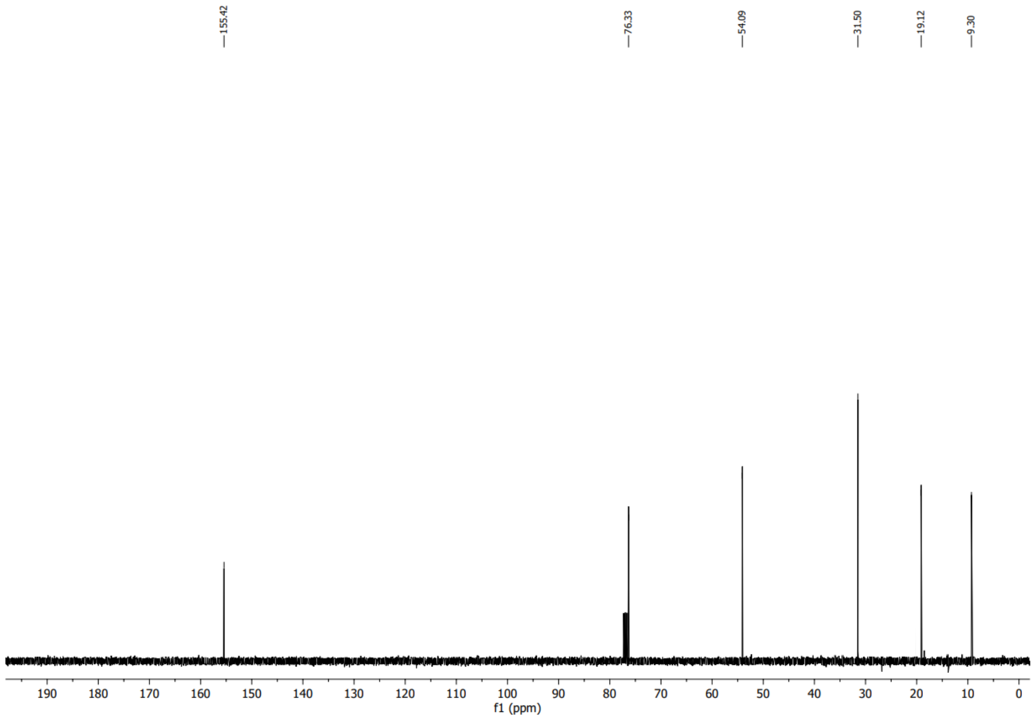

^{13}CNMR (100 MHz; $CDCl_3$) spectrum of compound **2g**.

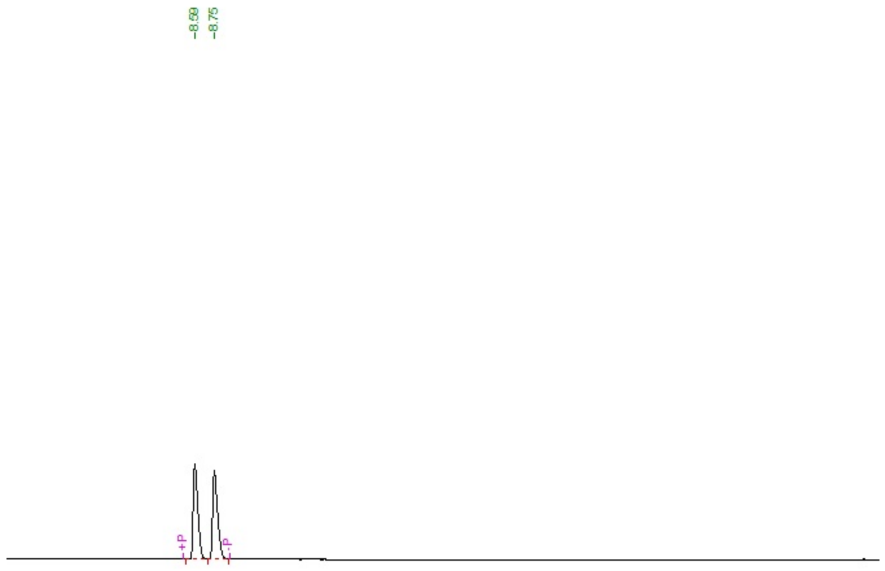

GC chromatogram (general GC method) of racemic compound **2g**.

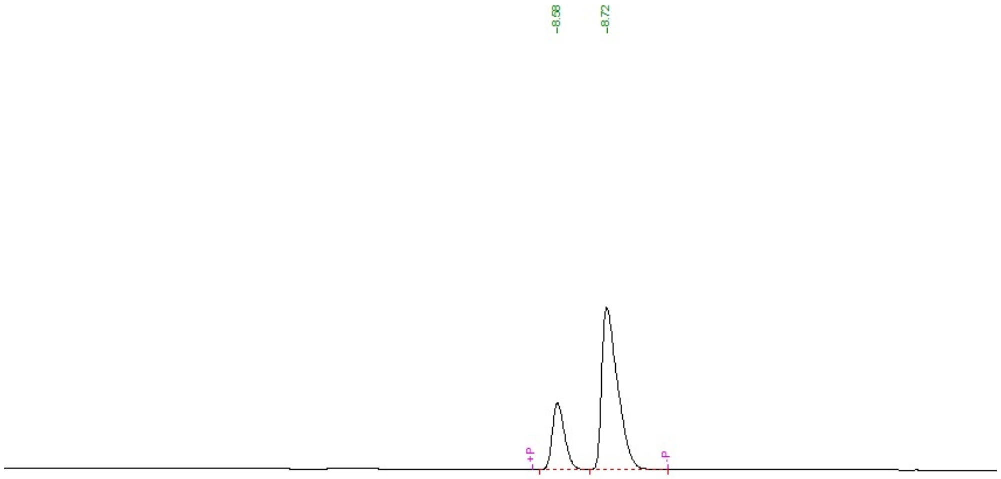

GC chromatogram (general GC method 1) of (*R*) – enantiomerically enriched (55 % *ee*) compound **2g**.

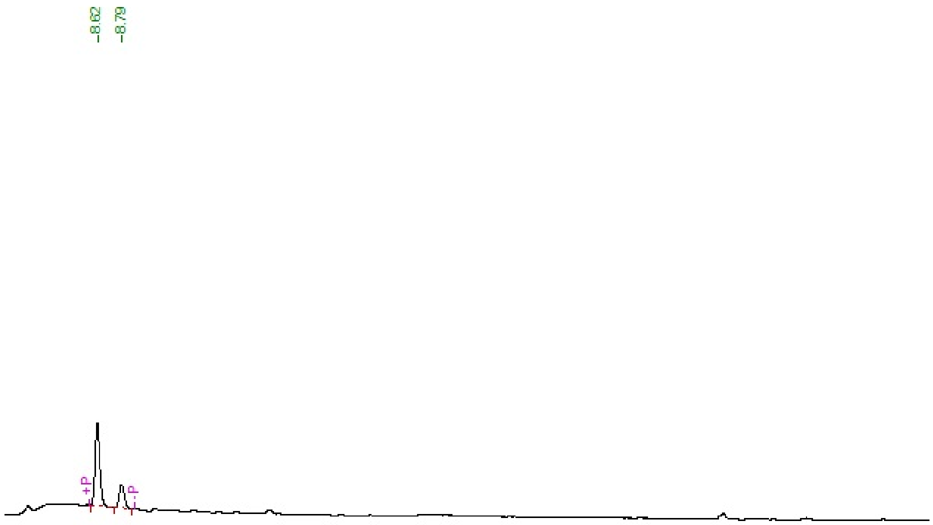

GC chromatogram (general GC method 1) of (*S*) – enantiomerically enriched (55 % *ee*) compound **2g**.

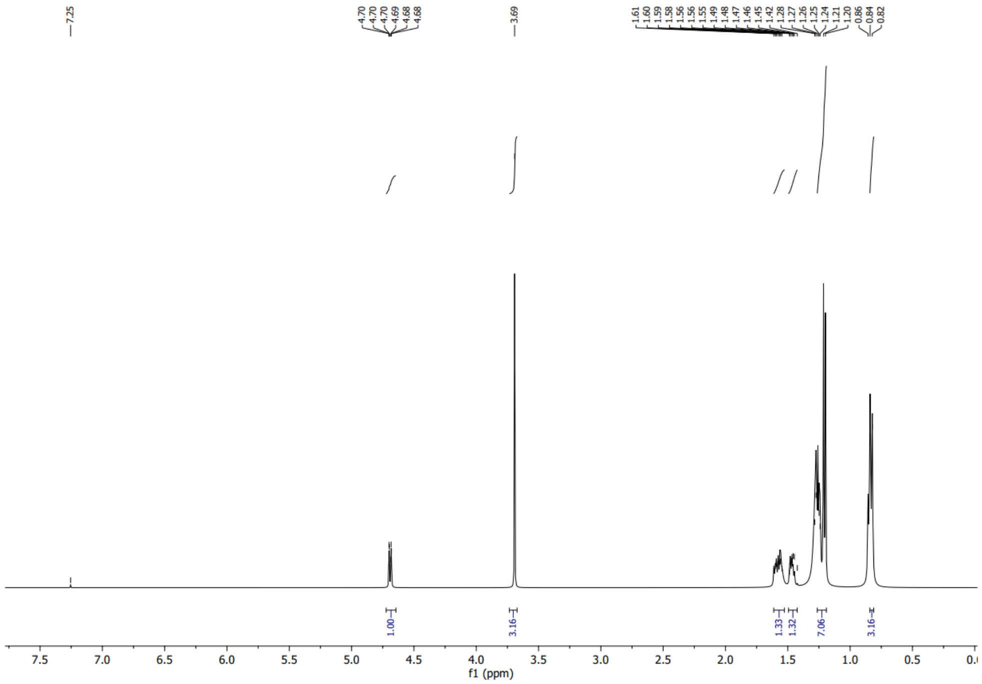

^{1}HNMR (400 MHz; $CDCl_3$) spectrum of compound **2h**.

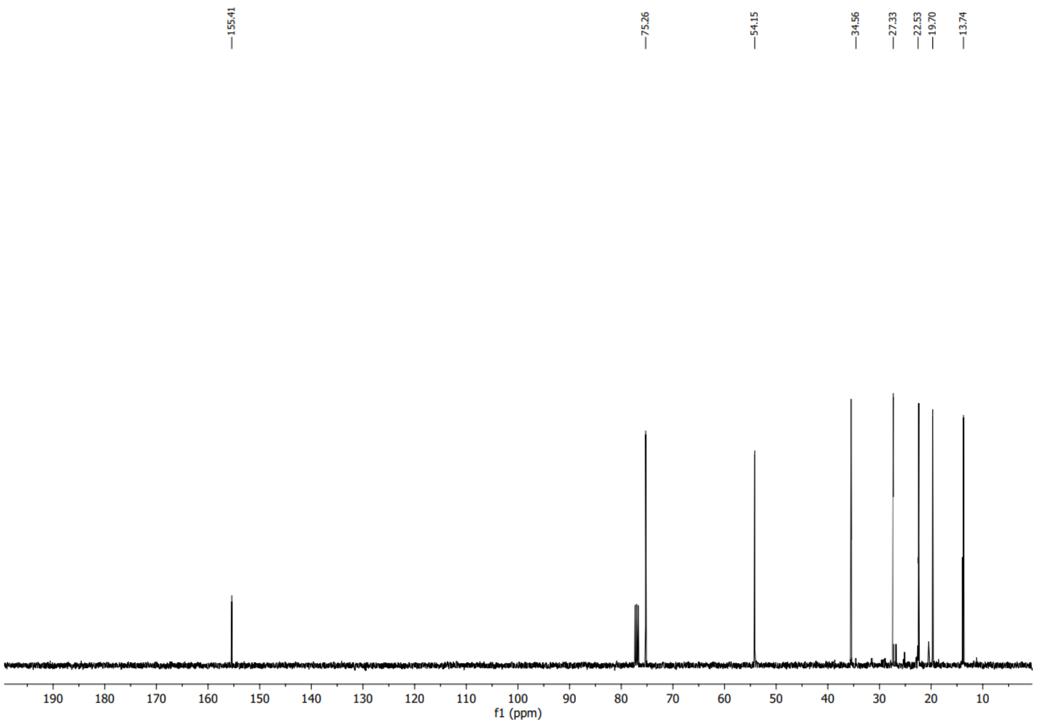

^{13}CNMR (100 MHz; CDCl3) spectrum of compound **2h**.

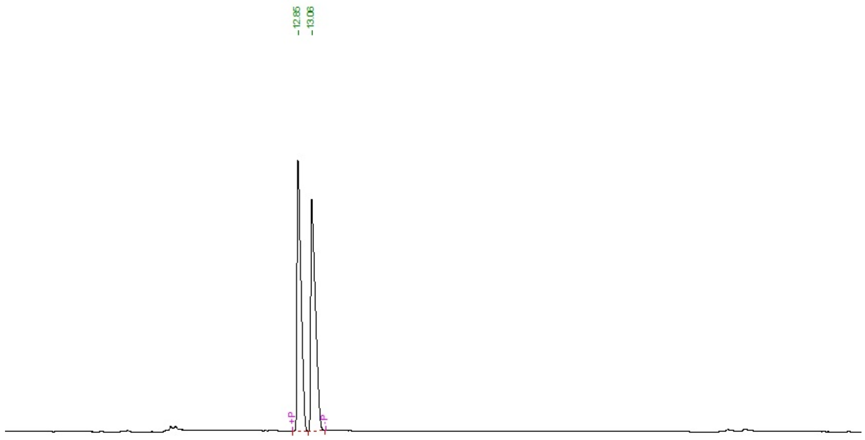

GC chromatogram (general GC method 1) of racemic compound **2h**.

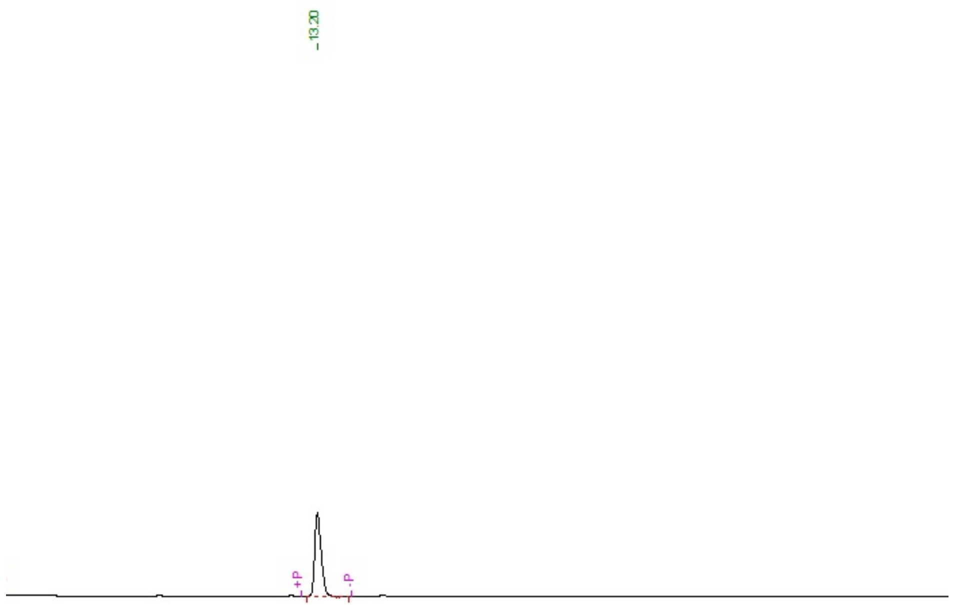

GC chromatogram (general GC method 1) of (*R*) – enantiomer of compound **2h**.

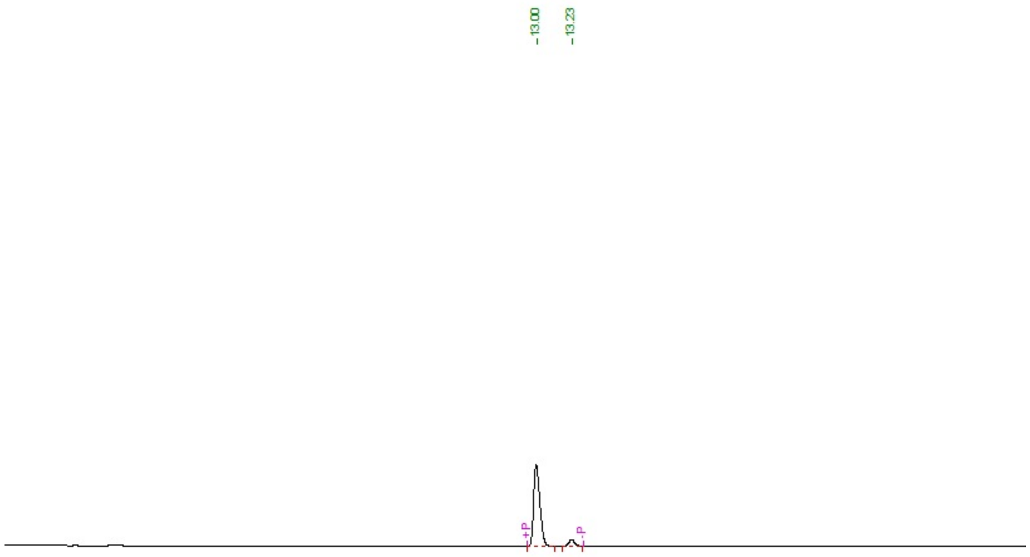

GC chromatogram (general GC method 1) of (*S*) – enantiomerically enriched (87 % *ee*) compound **2h**.

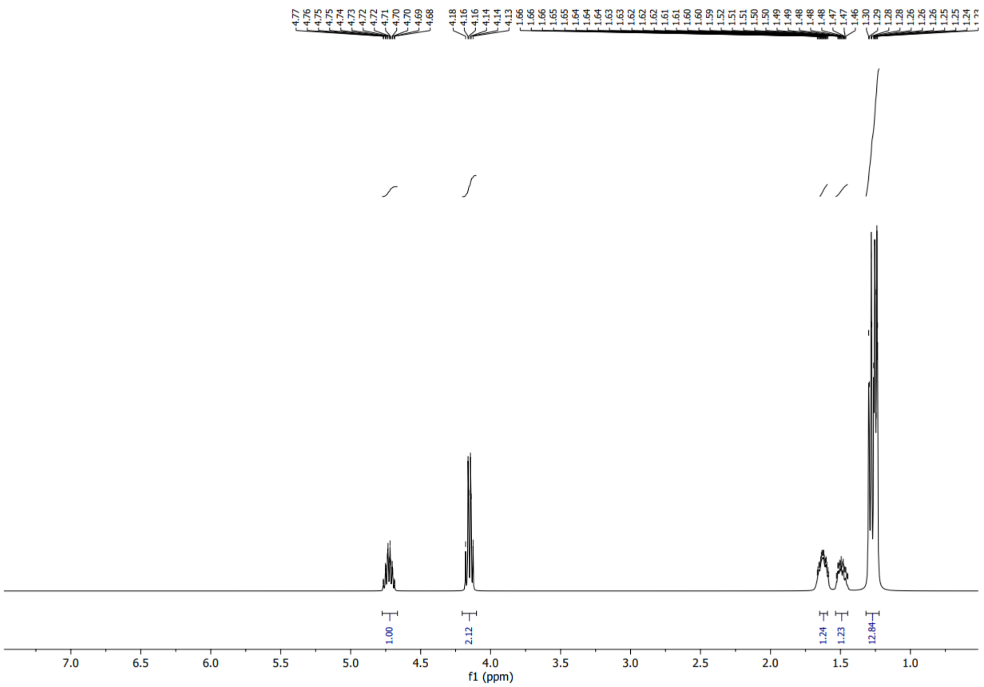

^{1}HNMR (400 MHz; $CDCl_3$) spectrum of compound **3h**.

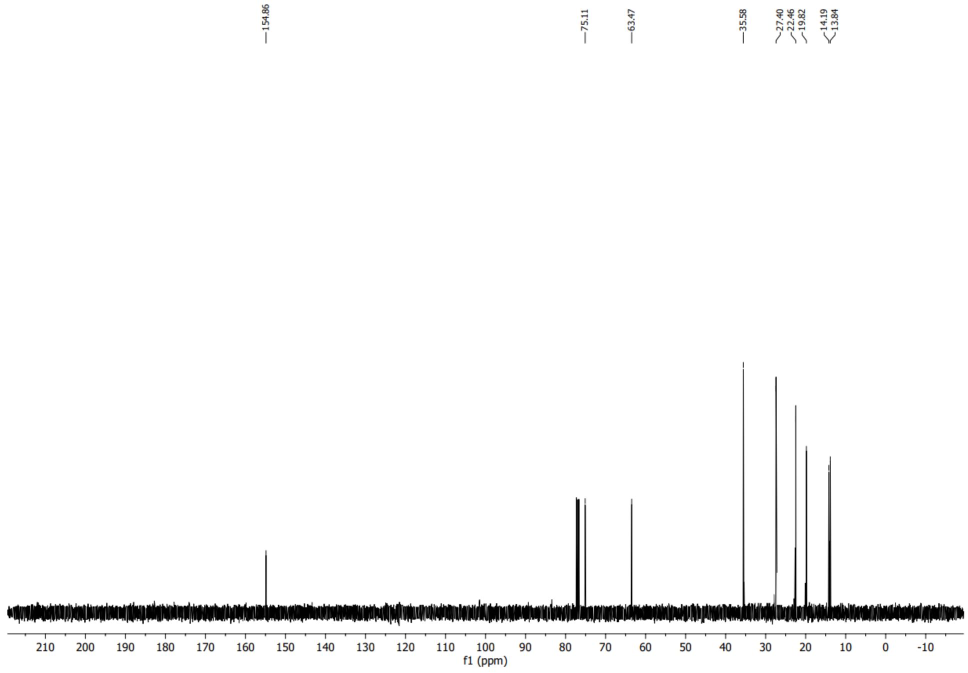

^{13}CNMR (100 MHz; $CDCl_3$) spectrum of compound **3h**.

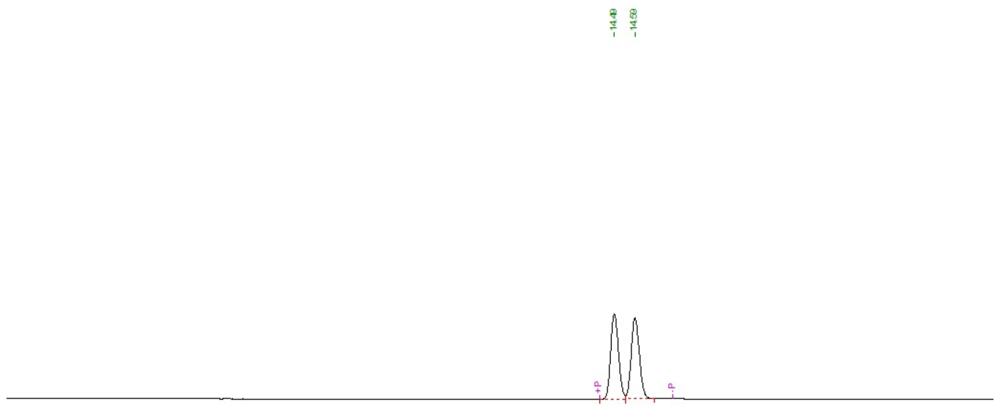

GC chromatogram (general GC method 1) of racemic compound **3h**.

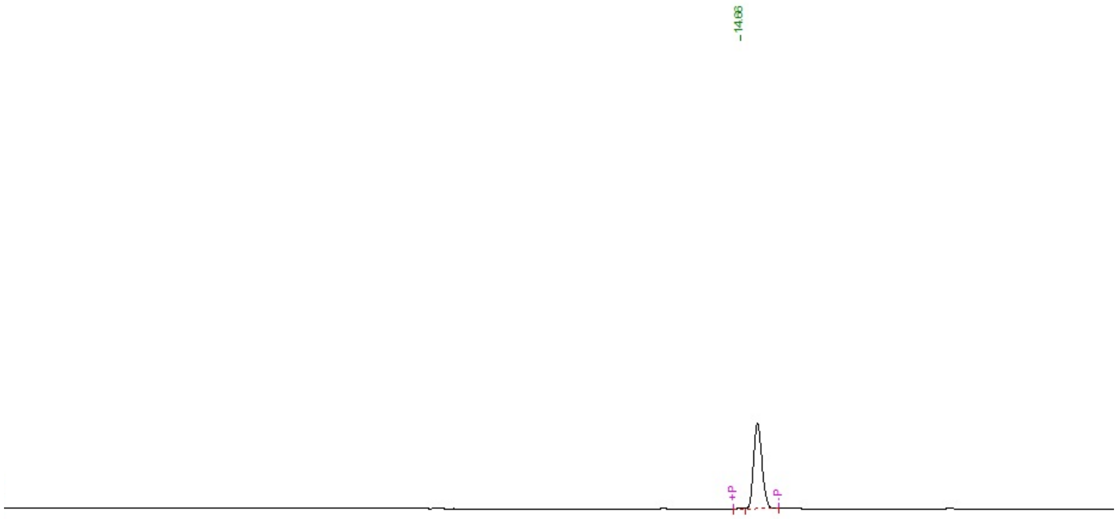

GC chromatogram (general GC method 1) of (*R*) – enantiomer of compound **3h**.

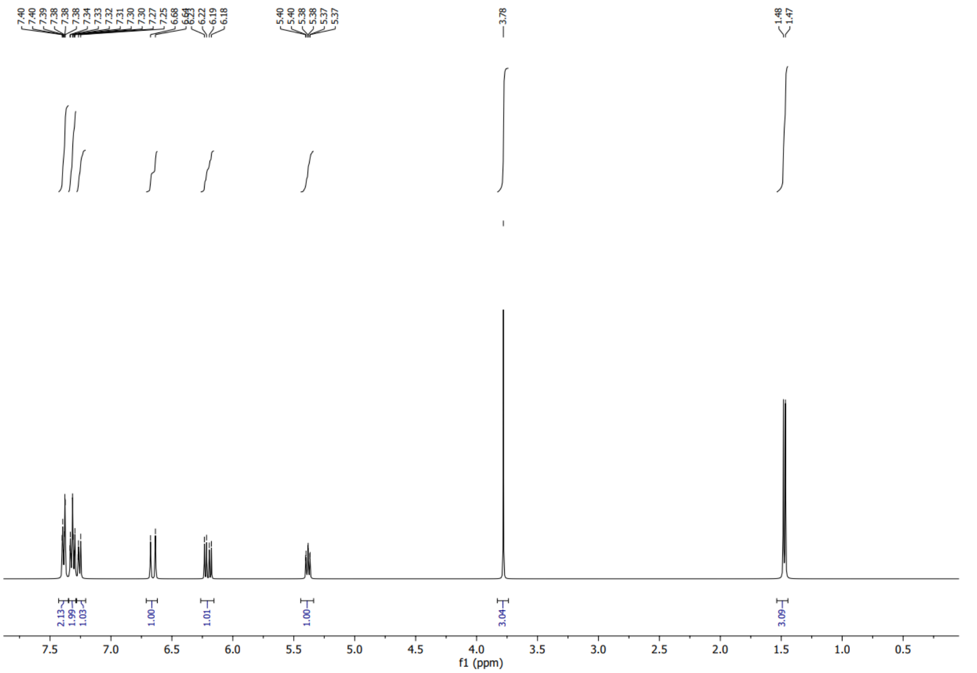

^{1}HNMR (400 MHz; $CDCl_3$) spectrum of compound **2i**.

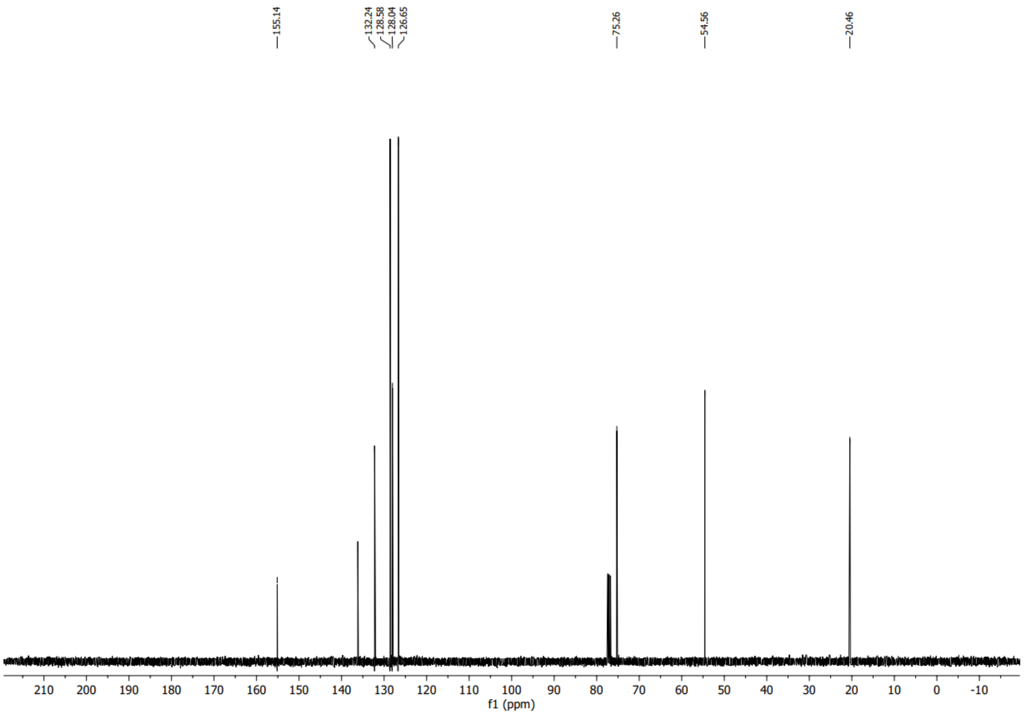

^{13}CNMR (100 MHz; $CDCl_3$) spectrum of compound **2i**.

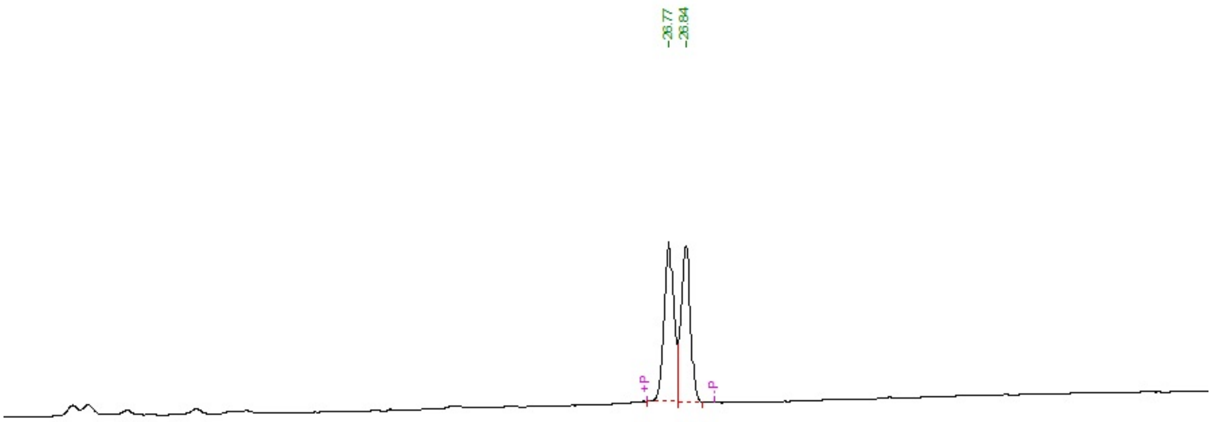

GC chromatogram (general GC method 1) of racemic compound **2i**.

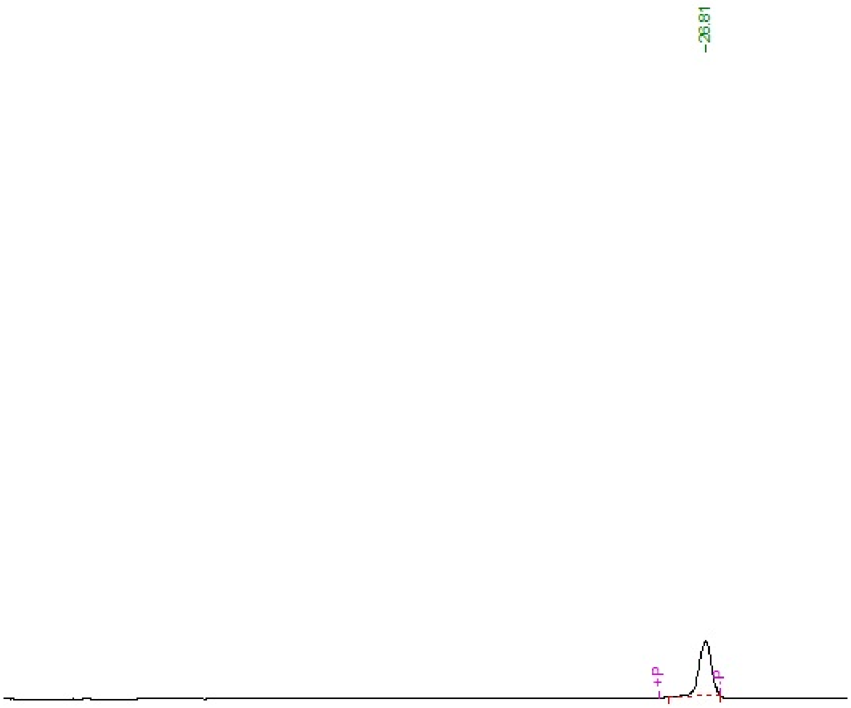

GC chromatogram (general GC method 1) of (*R*)-enantiomer of compound **2i**.

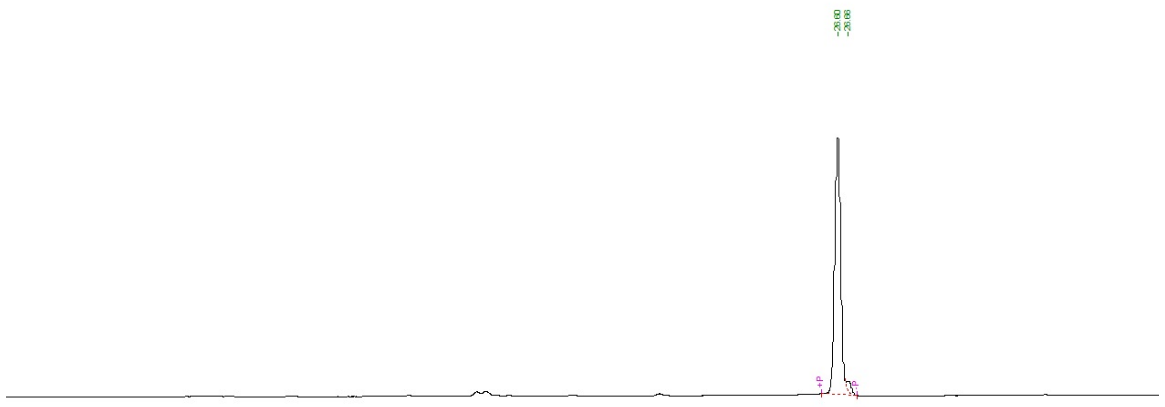

GC chromatogram (general GC method 1) of (*S*)-optically enriched (95 % *ee*) compound **2i**.

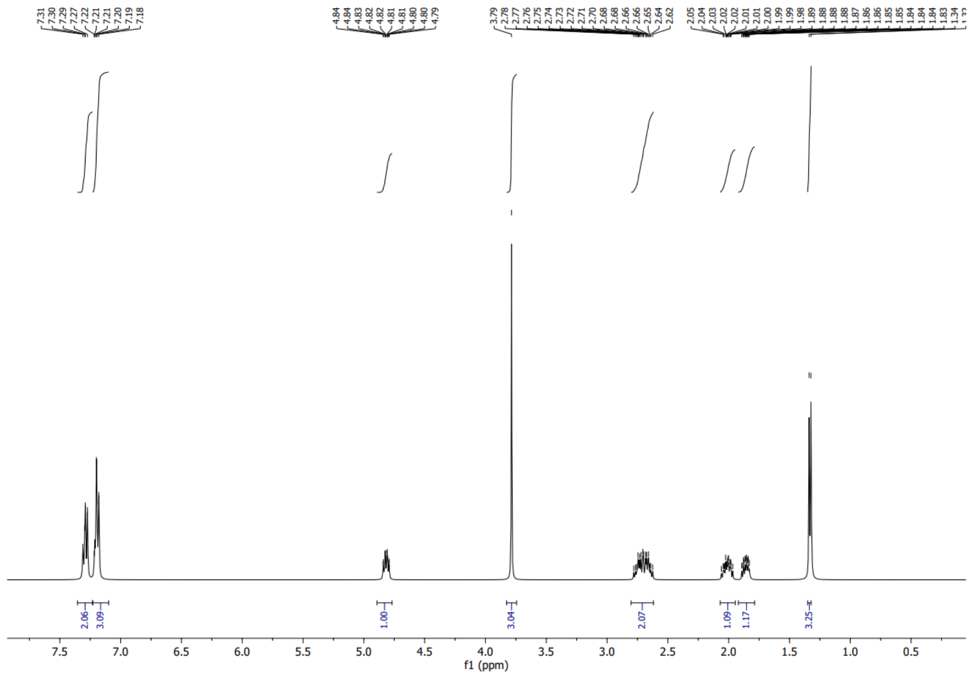

^{1}HNMR (400 MHz; $CDCl_3$) spectrum of compound **2j**.

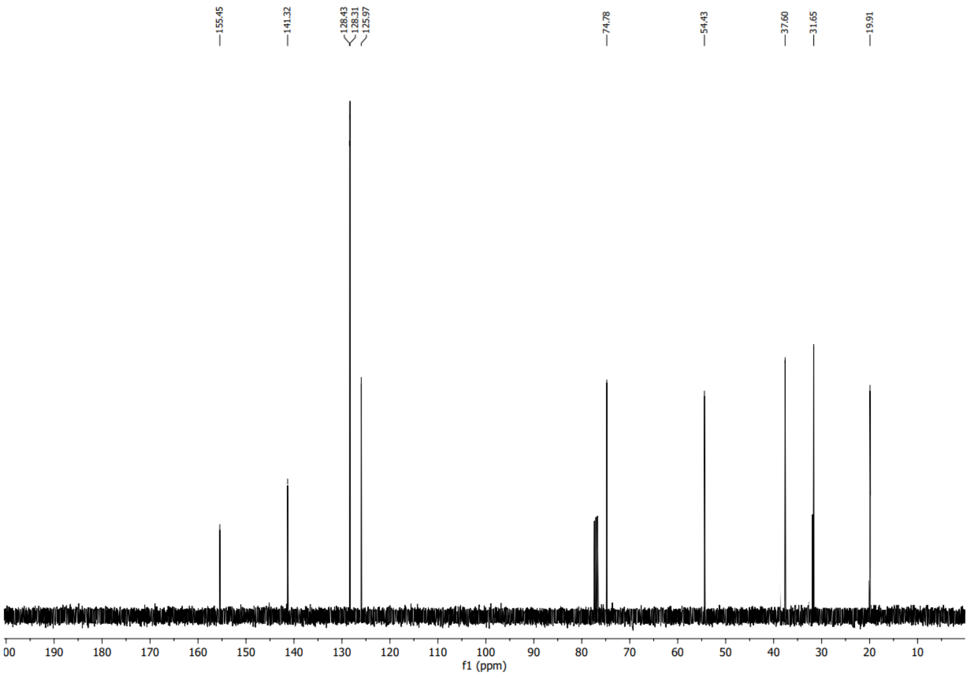

^{13}CNMR (100 MHz; $CDCl_3$) spectrum of compound **2j**.

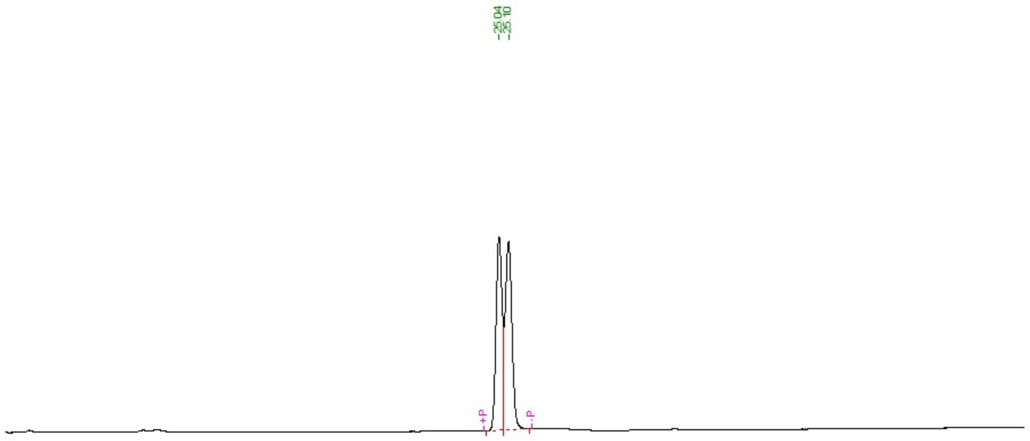

GC chromatogram (general GC method 1) of racemic compound **2j**.

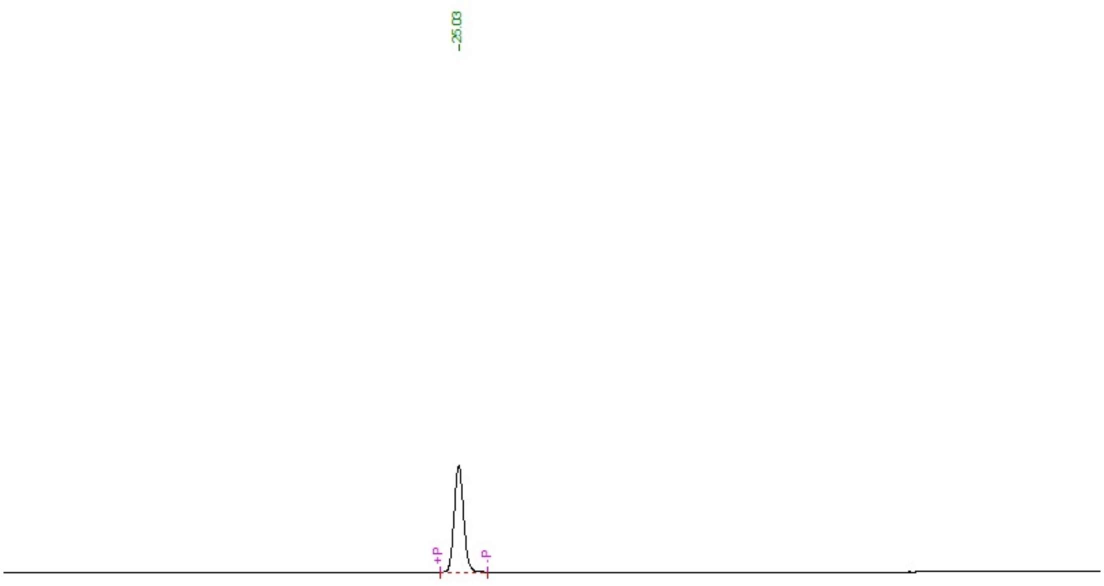

GC chromatogram (general GC method 1) of (*R*) enantiomer of compound **2j**.

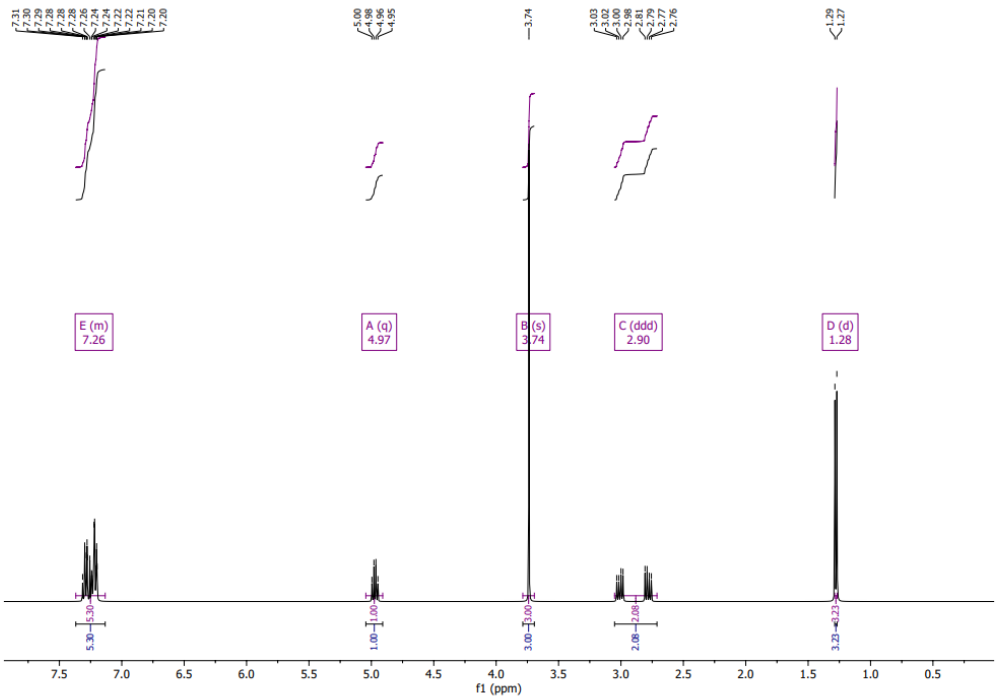

^{1}HNMR (400 MHz; $CDCl_3$) spectrum of compound **2k**.

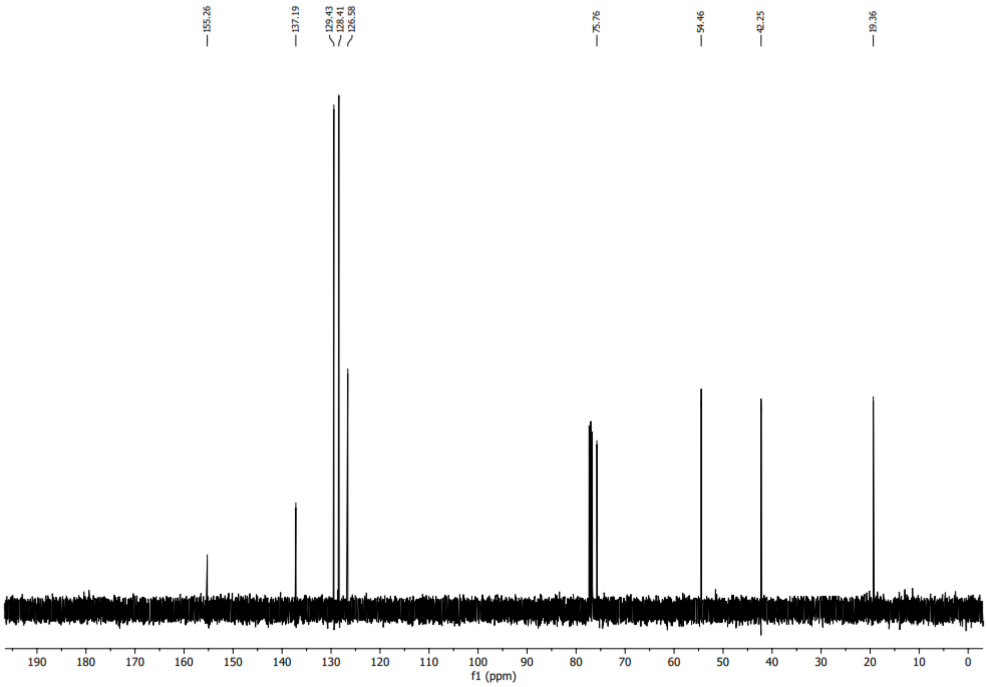

^{13}CNMR (100 MHz; $CDCl_3$) spectrum of compound **2k**.

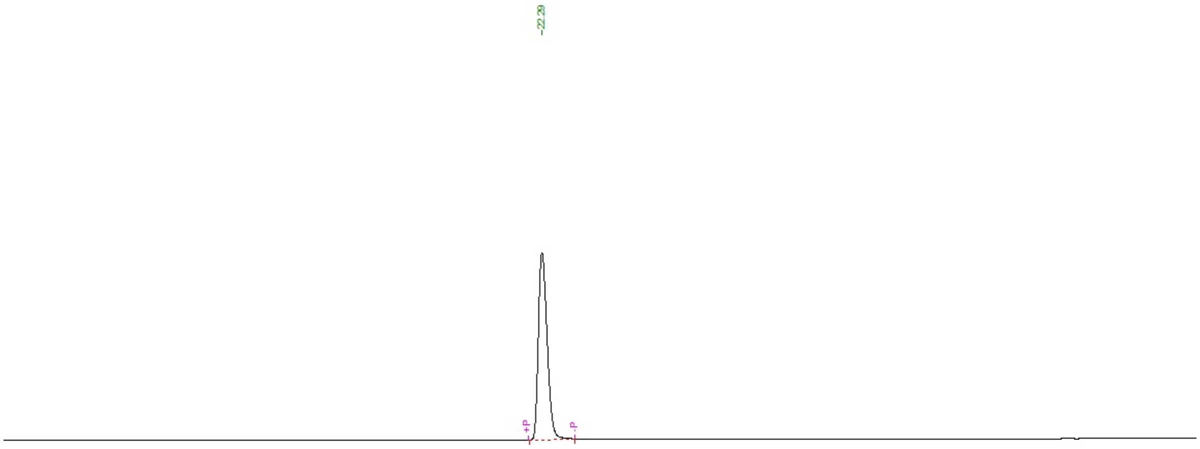

GC chromatogram (general GC method 2) of racemic compound **2k**.

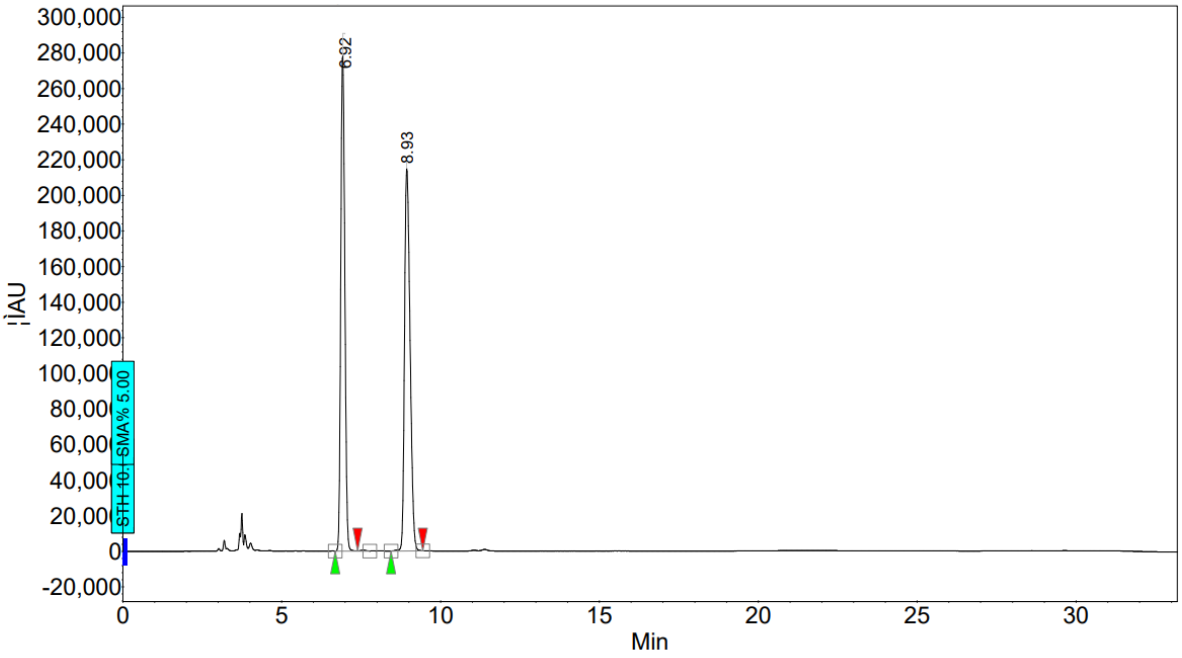

HPLC chromatogram (hexane:isopropanol 98:2) of racemic compound **2k** according to general method.

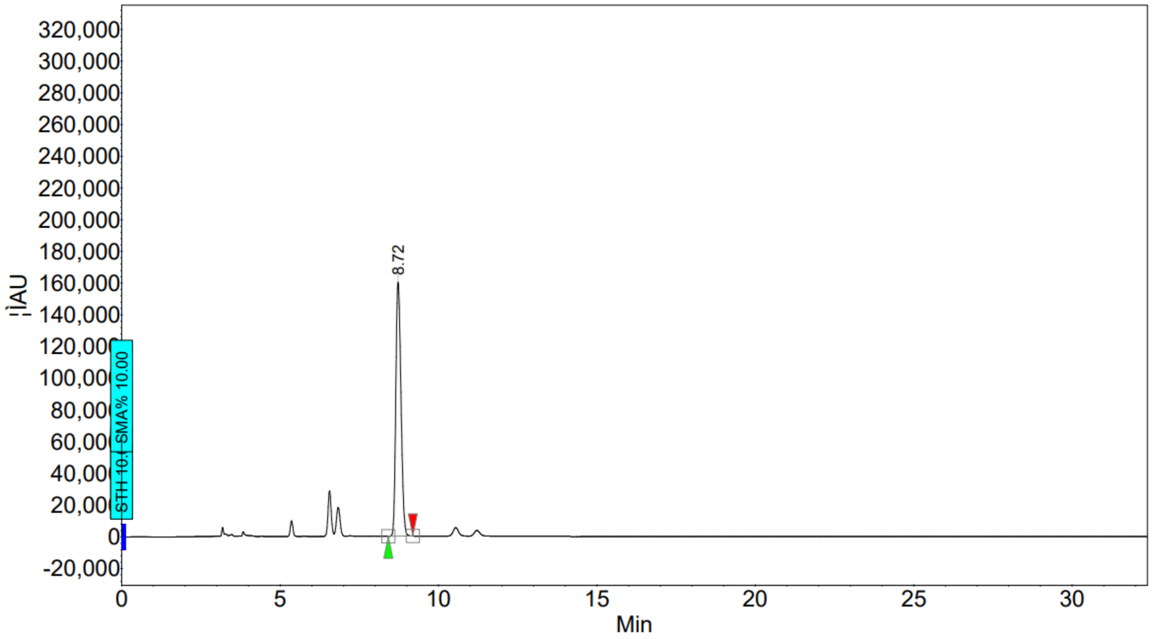

HPLC chromatogram (hexane:isopropanol 98:2) of (*R*) enantiomer of compound **2k** according to general method.

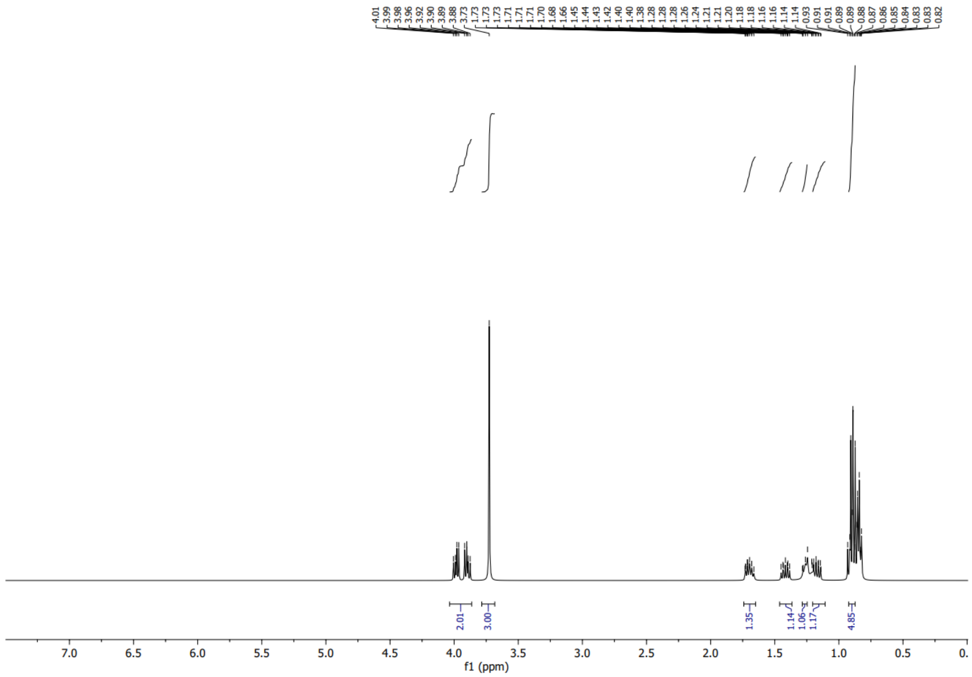

^{1}HNMR (400 MHz; $CDCl_3$) spectrum of compound **2l**.

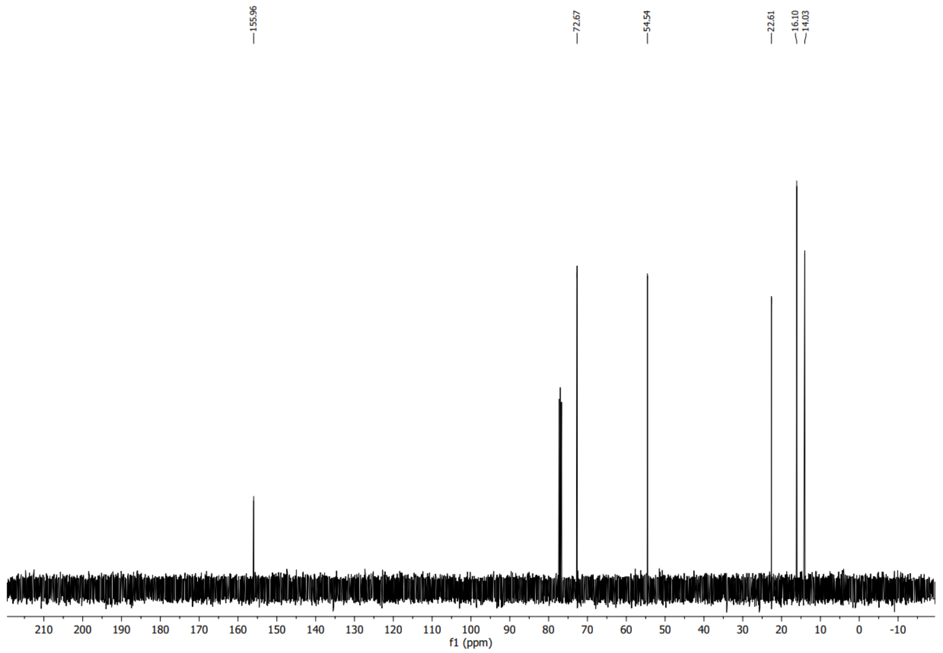

^{13}CNMR (100 MHz; $CDCl_3$) spectrum of compound **2l**.

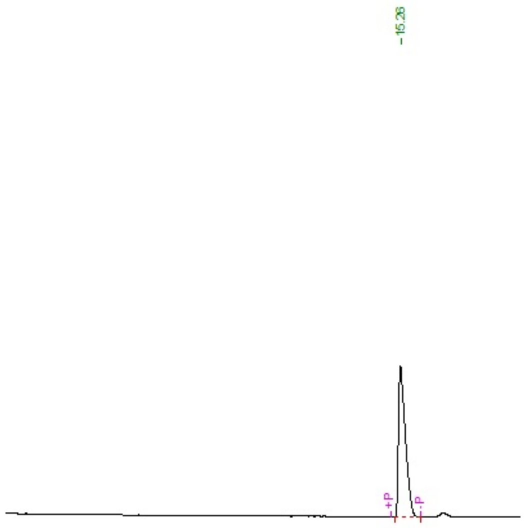

GC chromatogram (general GC method 2) of racemic compound **2l**.

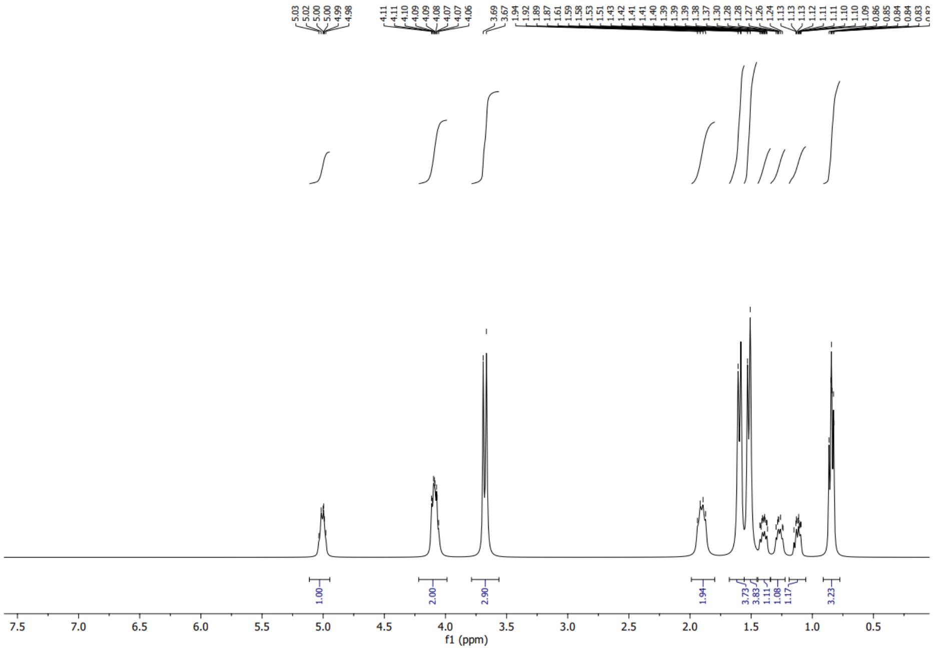

^{1}HNMR (400 MHz; $CDCl_3$) spectrum of compound **2m**.

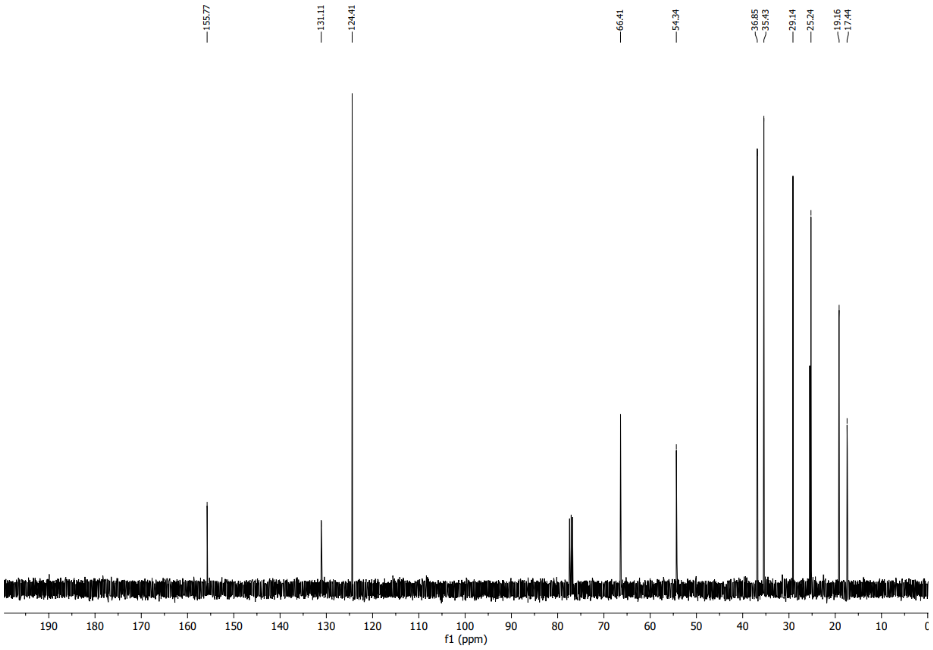

^{13}CNMR (100 MHz; $CDCl_3$) spectrum of compound **2m**.

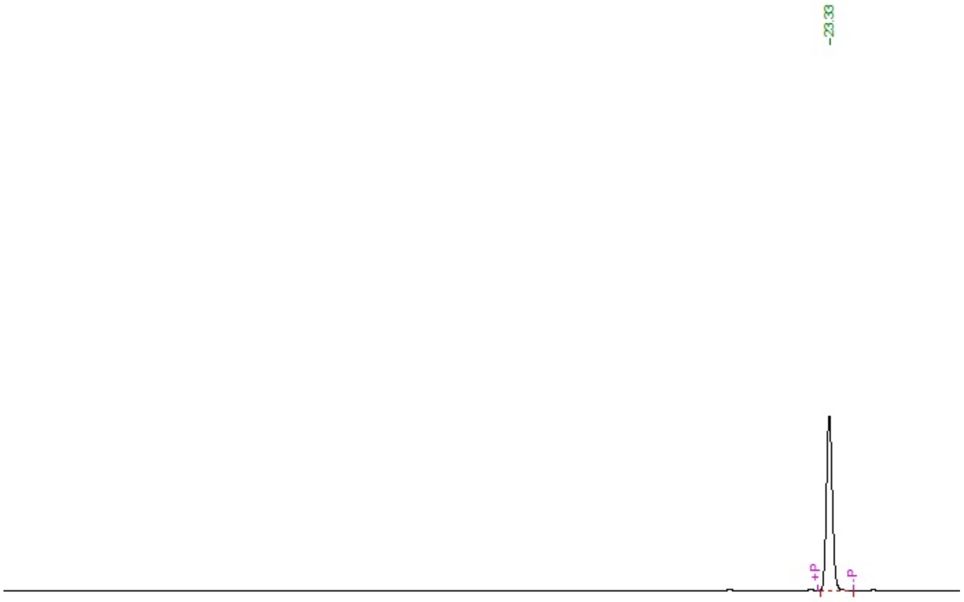

GC chromatogram (general GC method 1) of racemic compound **2m**.

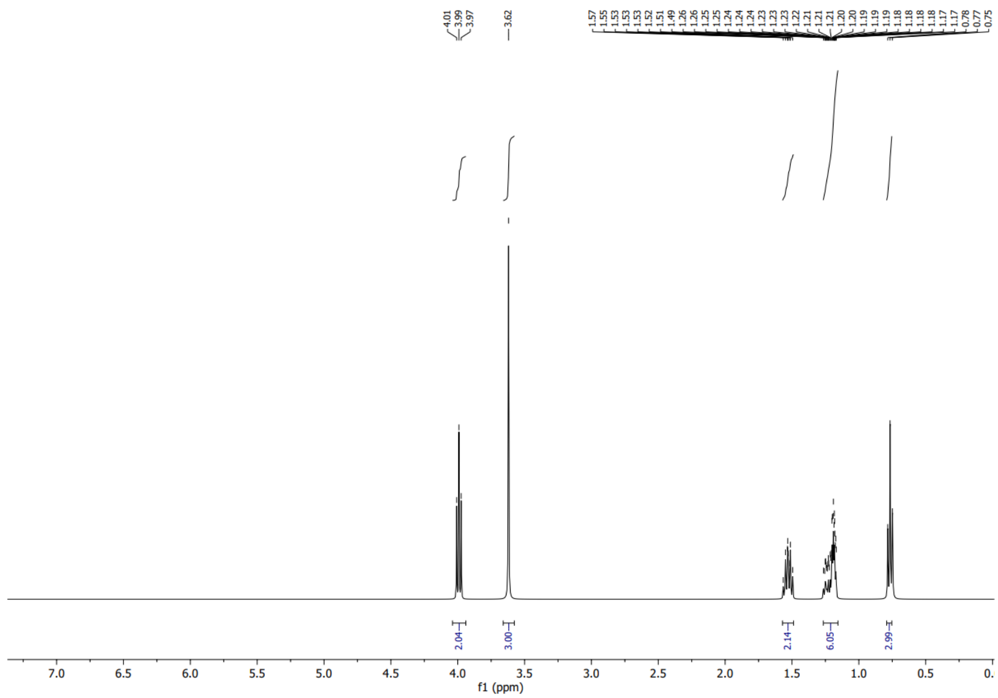

^{1}HNMR (400 MHz; $CDCl_3$) spectrum of compound **2n**.

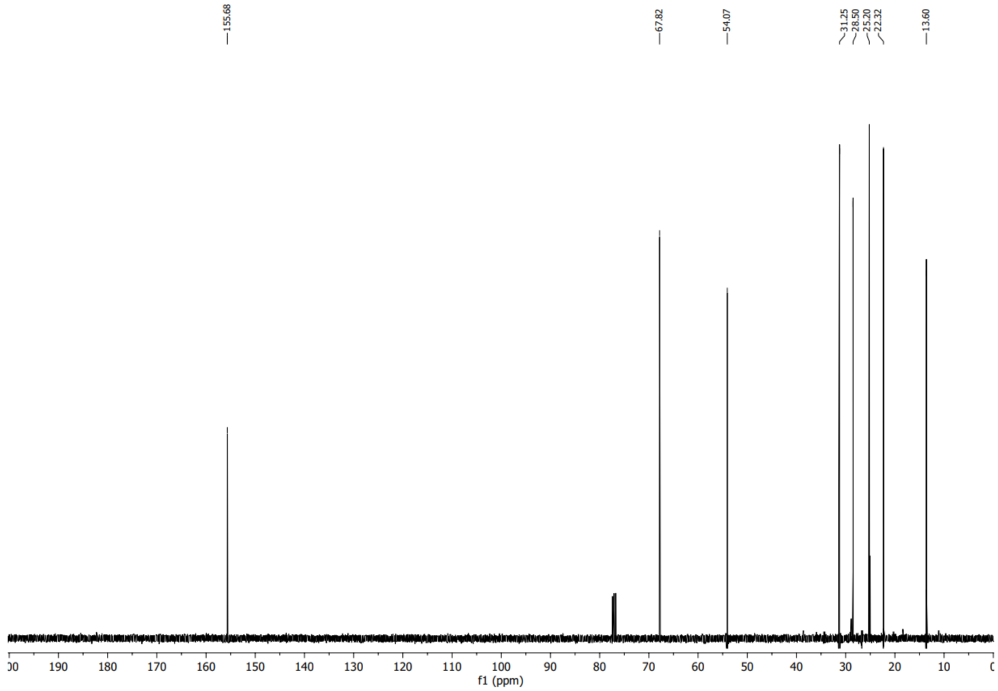

^{13}CNMR (100 MHz; $CDCl_3$) spectrum of compound **2n**.

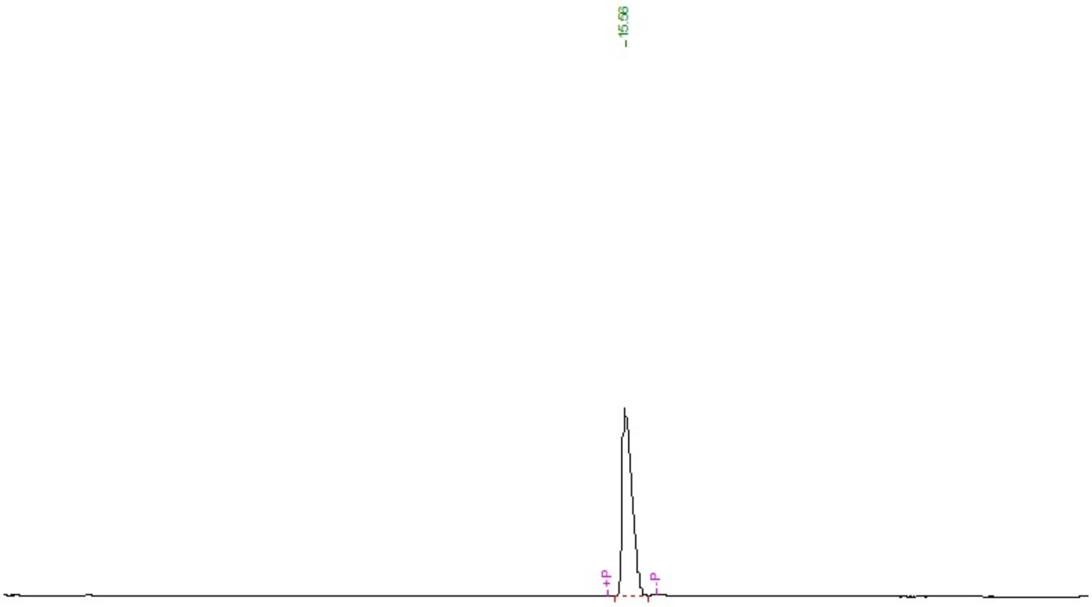

GC chromatogram (general GC method 1) of compound **2n**.

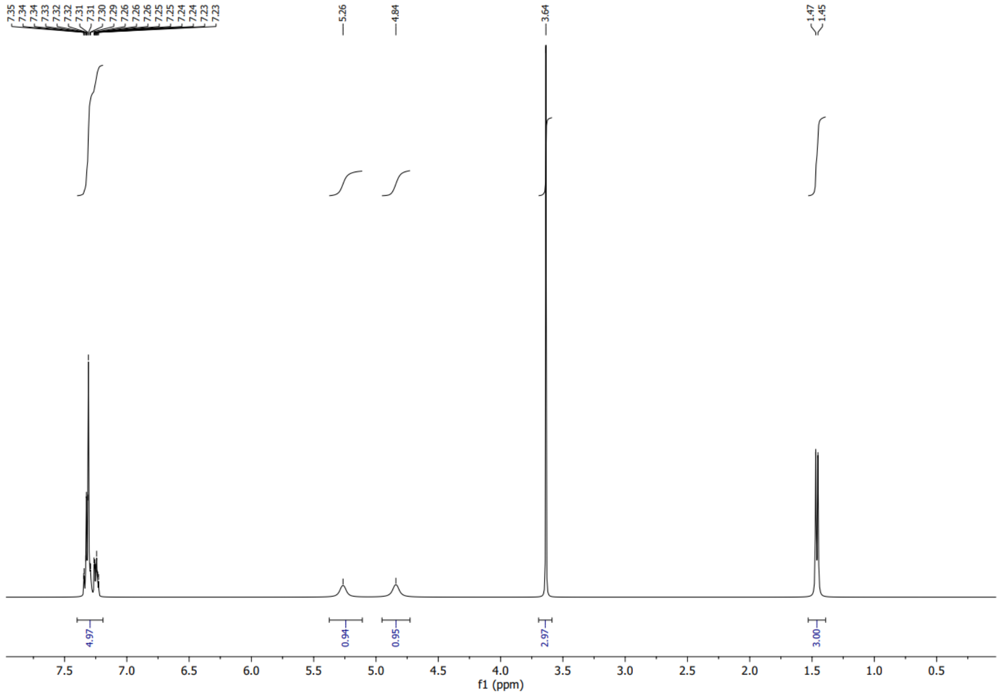

^{1}HNMR (400 MHz; $CDCl_3$) spectrum of compound **2o**.

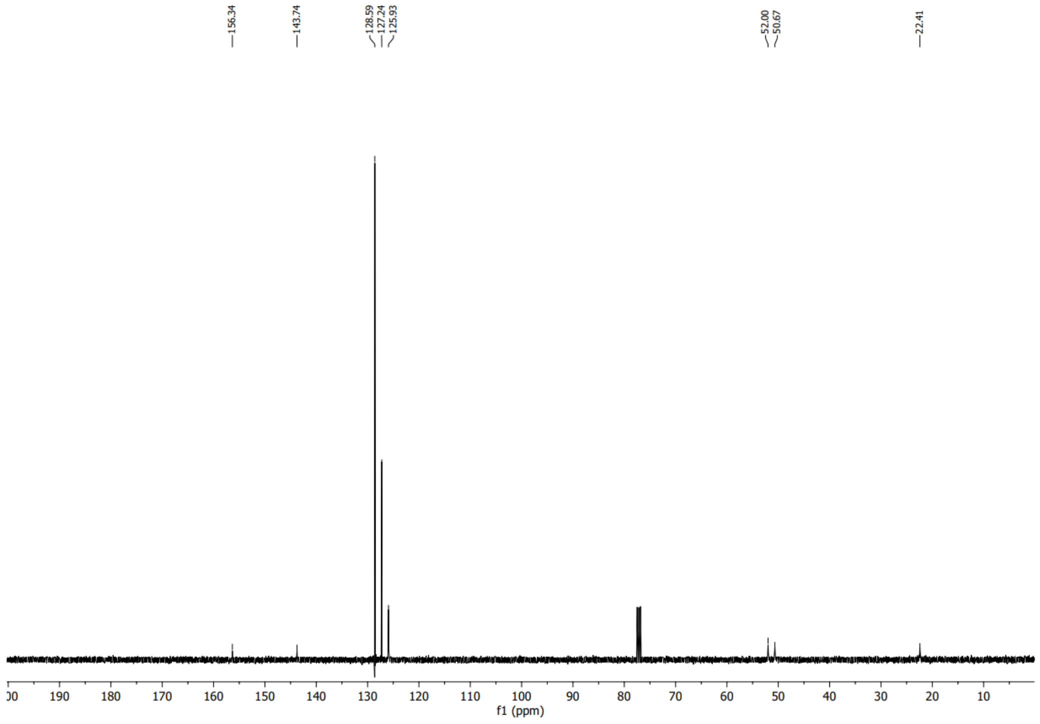

^{13}CNMR (100 MHz; $CDCl_3$) spectrum of compound **2o**.

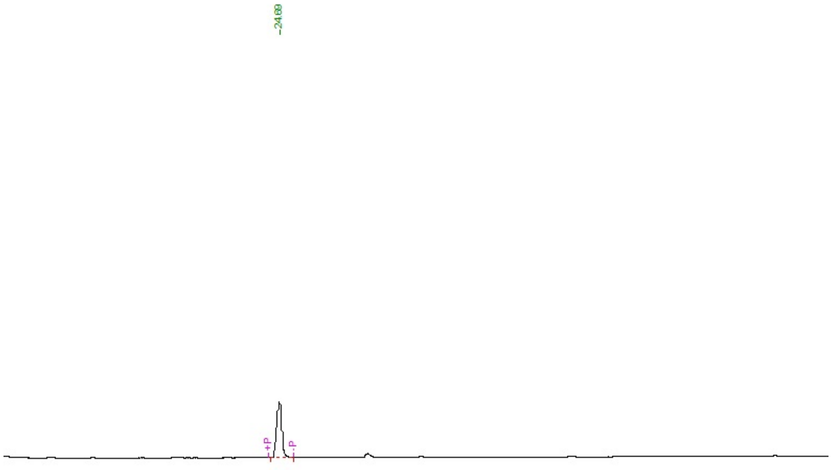

GC chromatogram (general GC method 1) of compound **2o**.

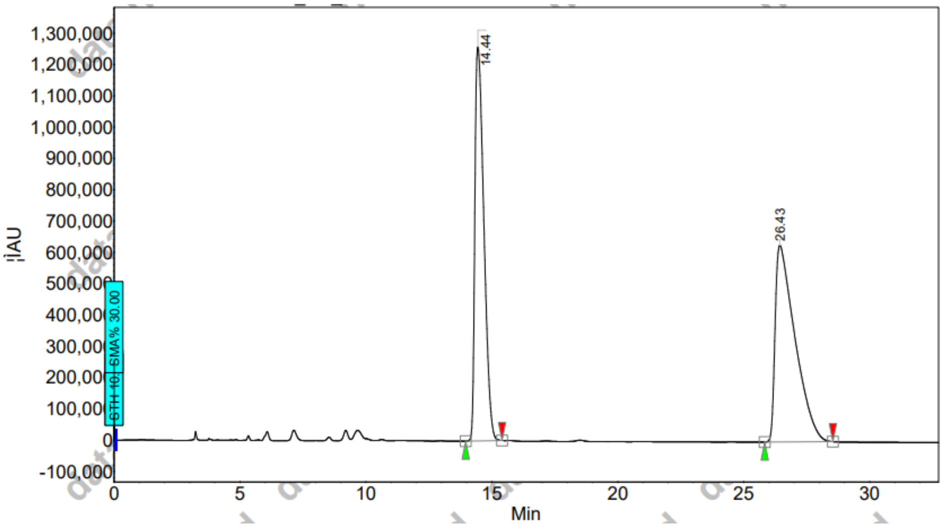

HPLC chromatogram (hexane:isopropanol 94:6) of racemic compound **2o** according to general method.

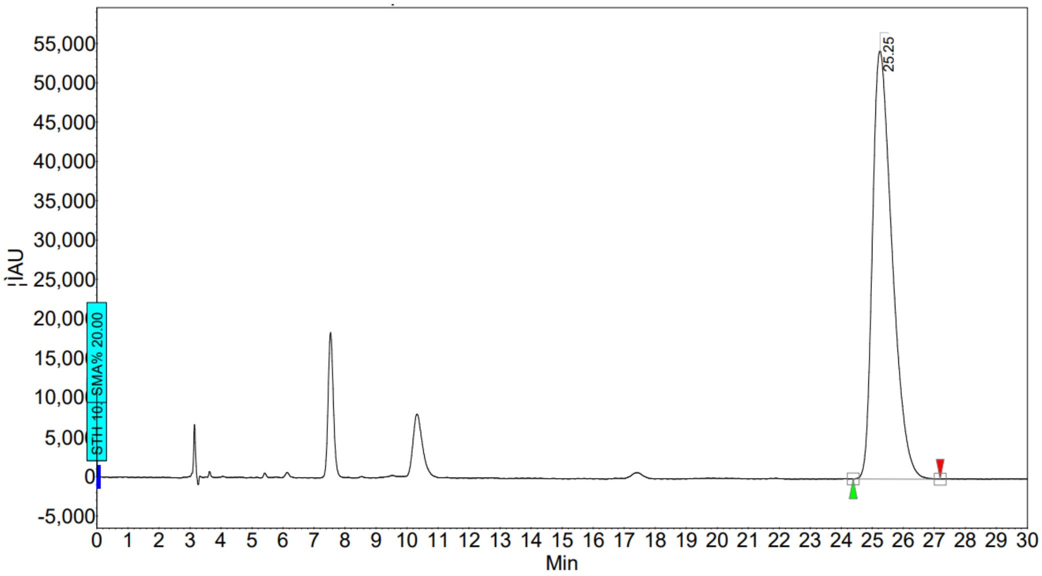

HPLC chromatogram (hexane:isopropanol 94:6) of (*R*)-enantiomer of compound **2o** according to general method.

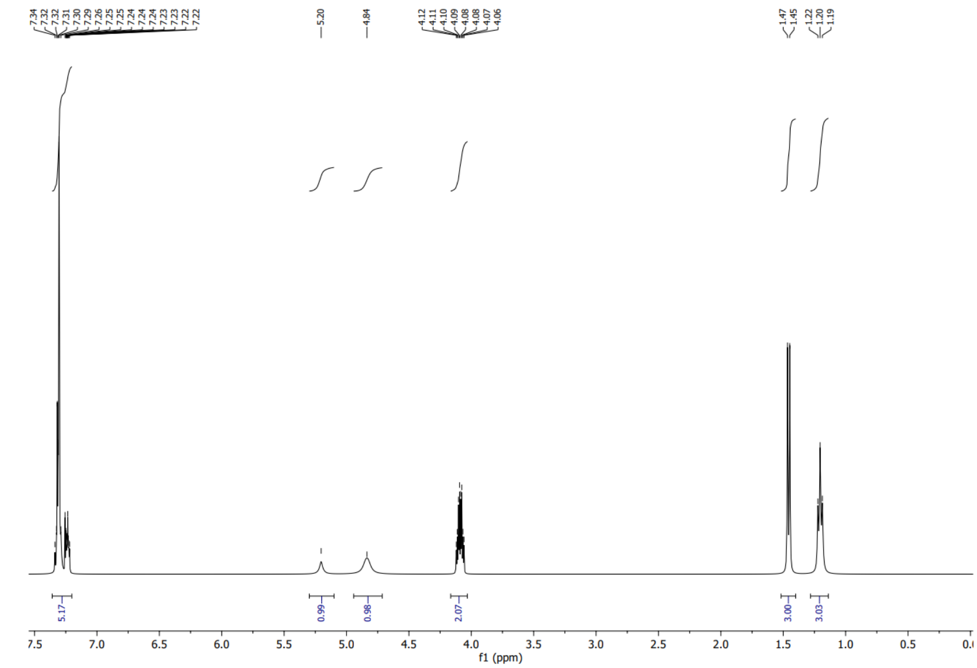

^{1}HNMR (400 MHz; $CDCl_3$) spectrum of compound **3o**.

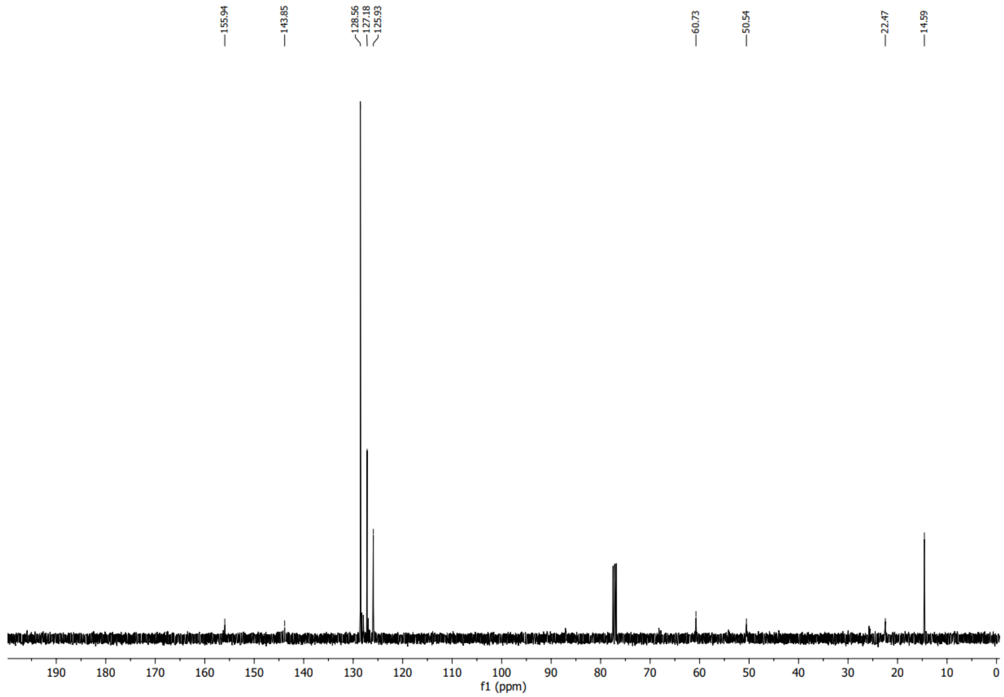

^{13}CNMR (100 MHz; $CDCl_3$) spectrum of compound **3o**.

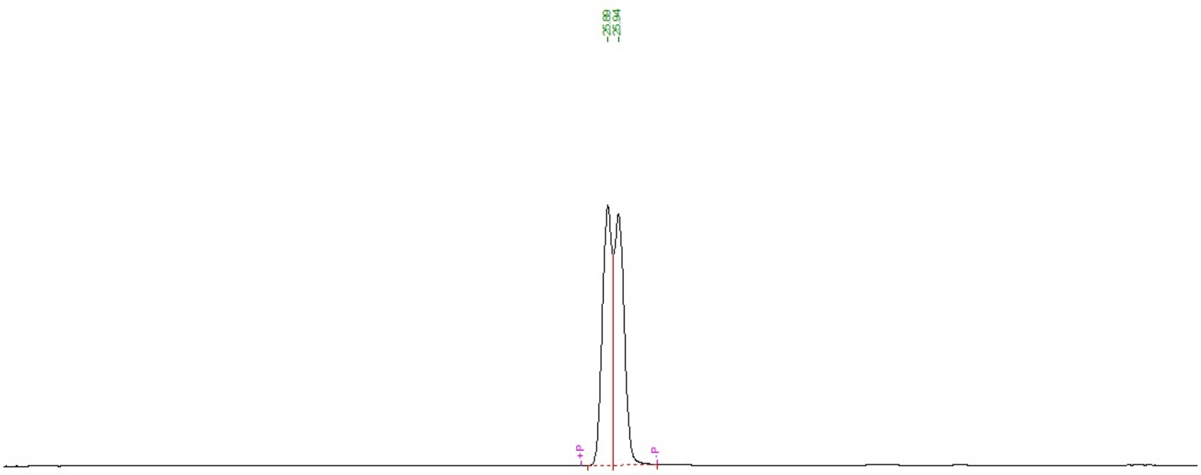

GC chromatogram (general GC method 1) of compound **3o**.

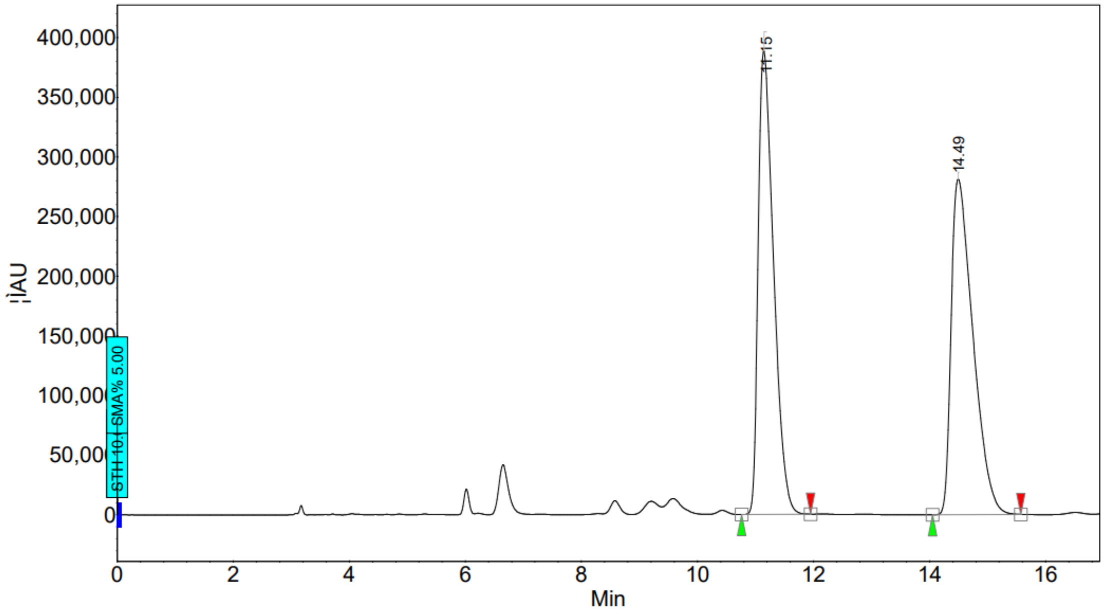

HPLC chromatogram (hexane:isopropanol 94:6 column: OM, T=15 °C) of compound **3o**.

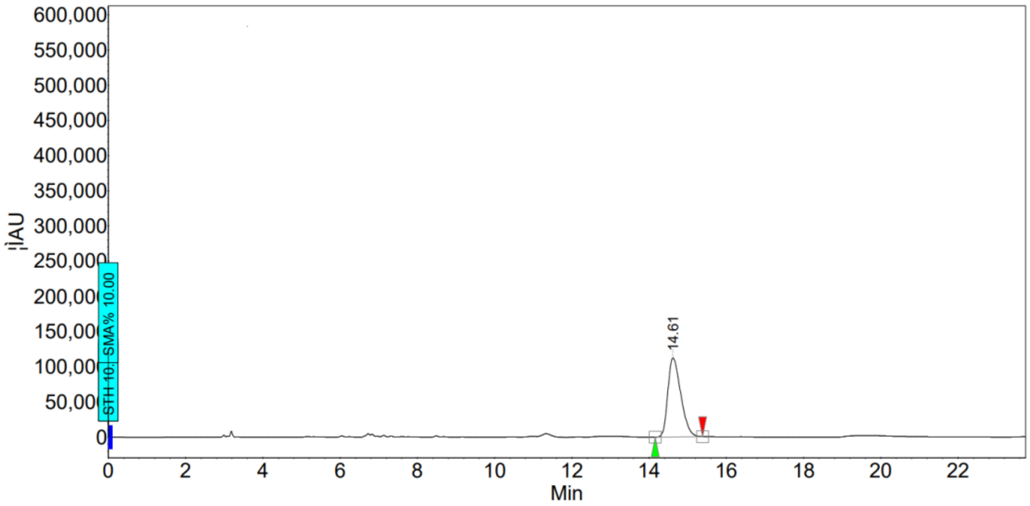

HPLC chromatogram (hexane:isopropanol 94:6 column: OM, T=15 °C) of (*R*)-enantiomer of compound **3o**.

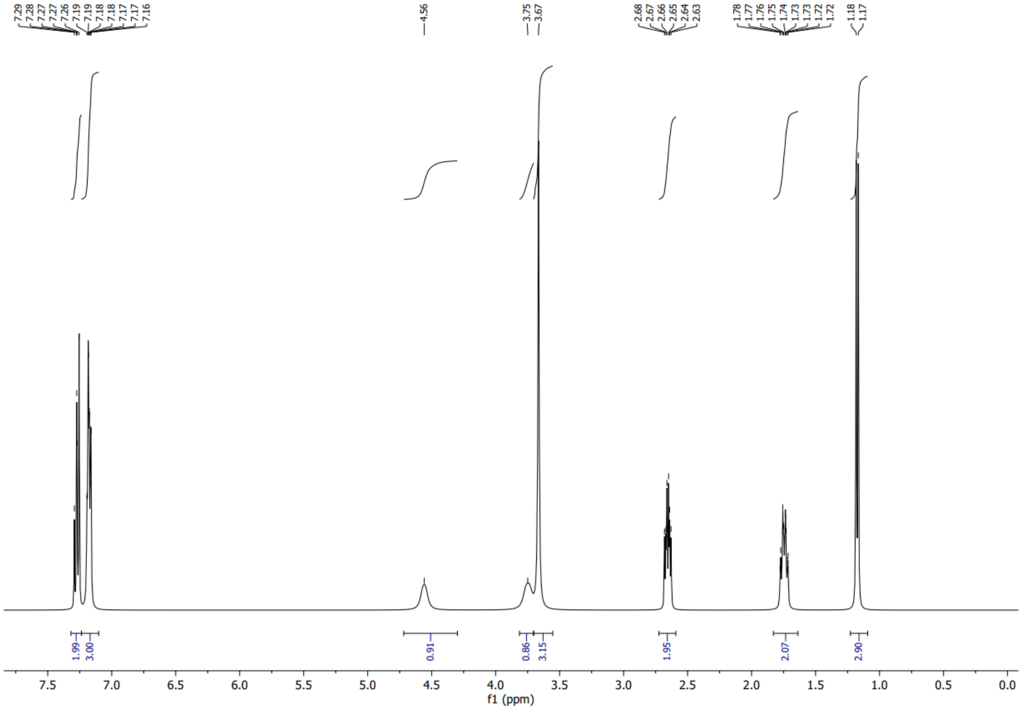

^{1}HNMR (400 MHz; $CDCl_3$) spectrum of compound **2p**.

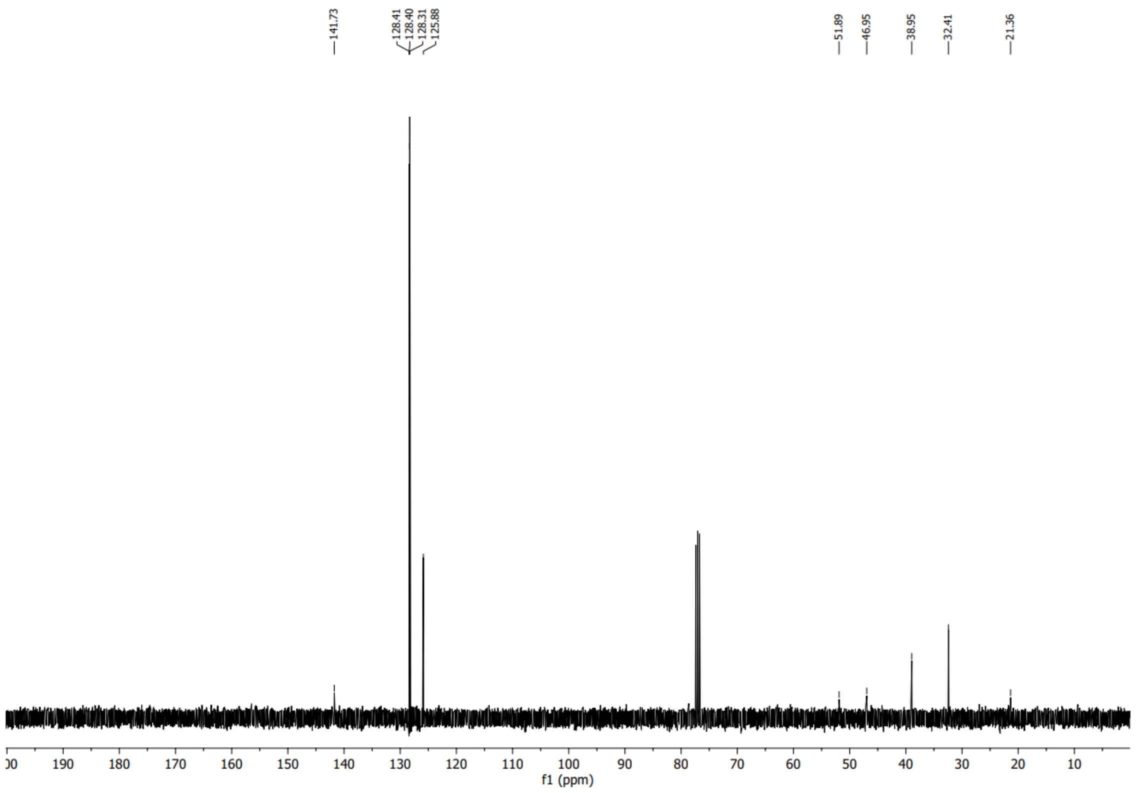

^{13}CNMR (100 MHz; $CDCl_3$) spectrum of compound **2p**.

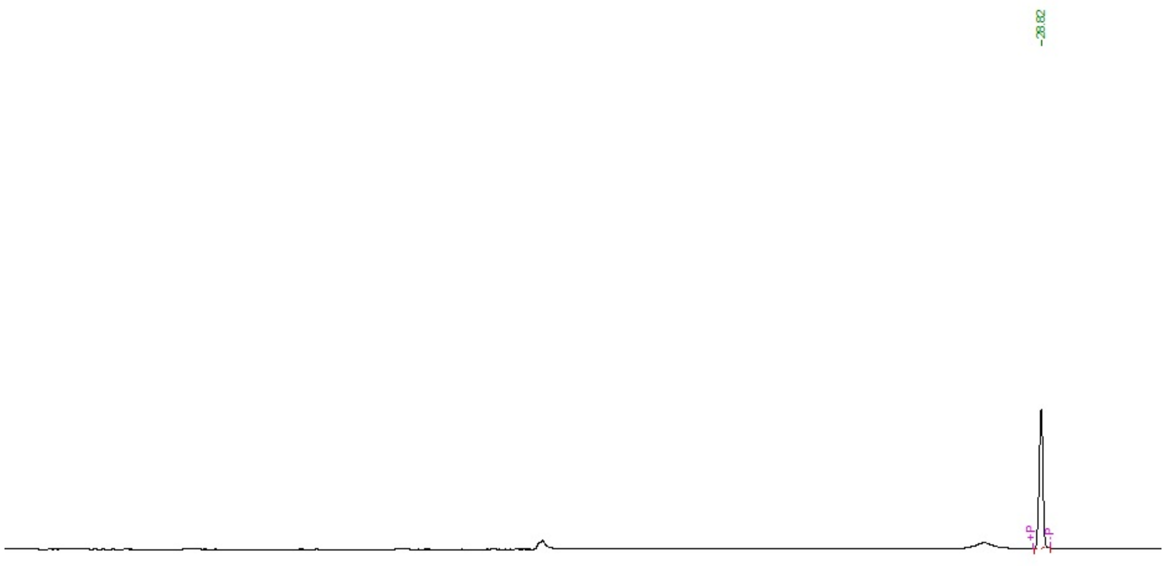

GC chromatogram (general GC method 1) of compound **2p**.

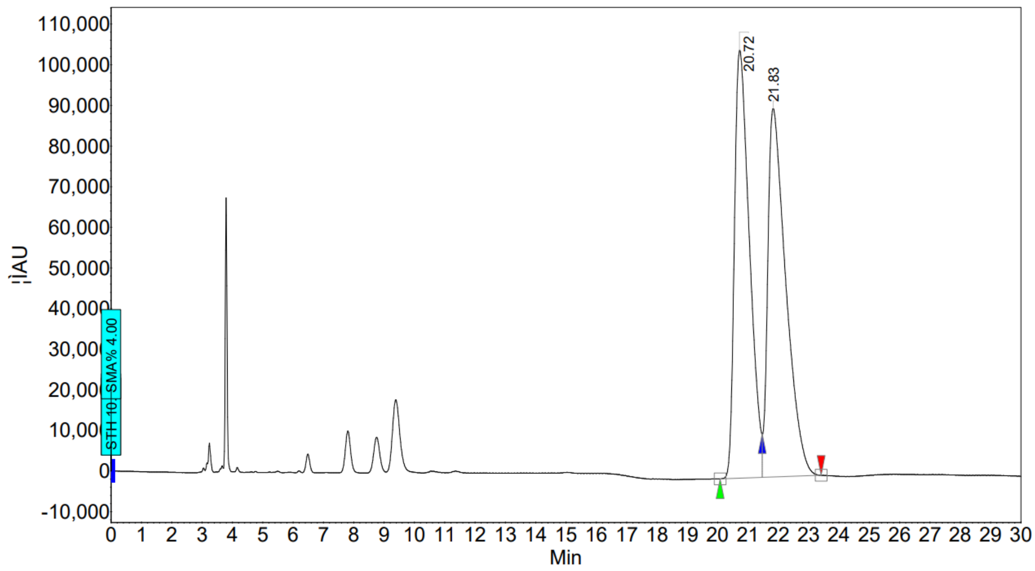

HPLC chromatogram (hexane:isopropanol : 95:5; column: OM) racemic of compound **2p**.

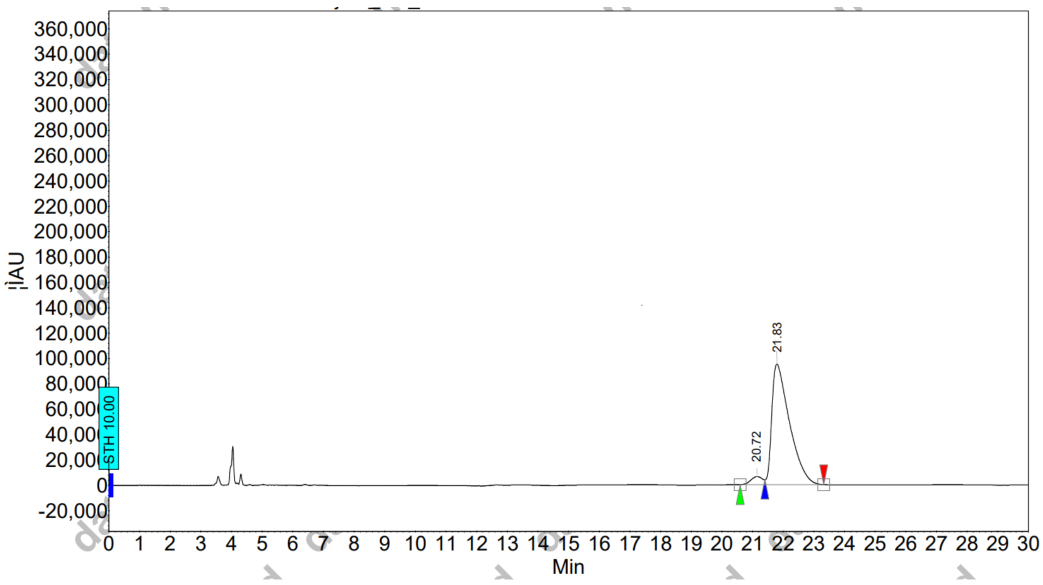

HPLC chromatogram (hexane:isopropanol : 95:5; column: OM) of *R*-enantio enriched (92 % *ee*) compound **2p**.

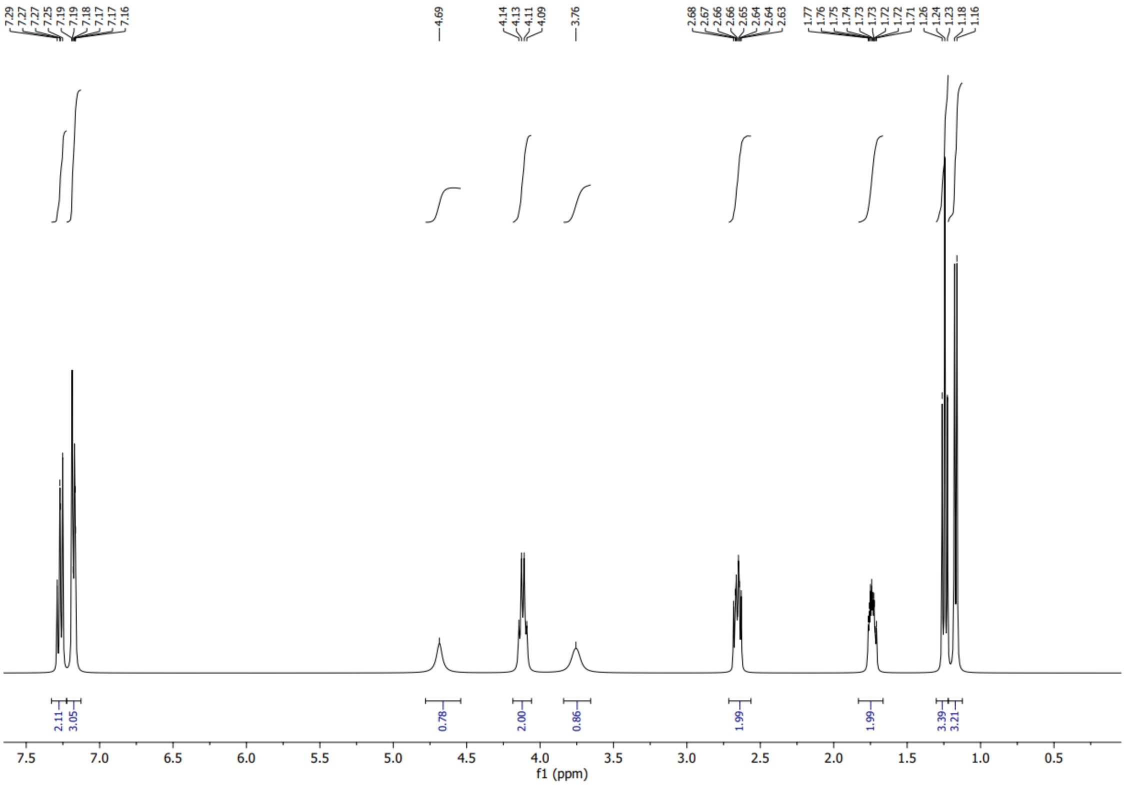

^{1}HNMR (400 MHz; $CDCl_3$) spectrum of compound **3p**.

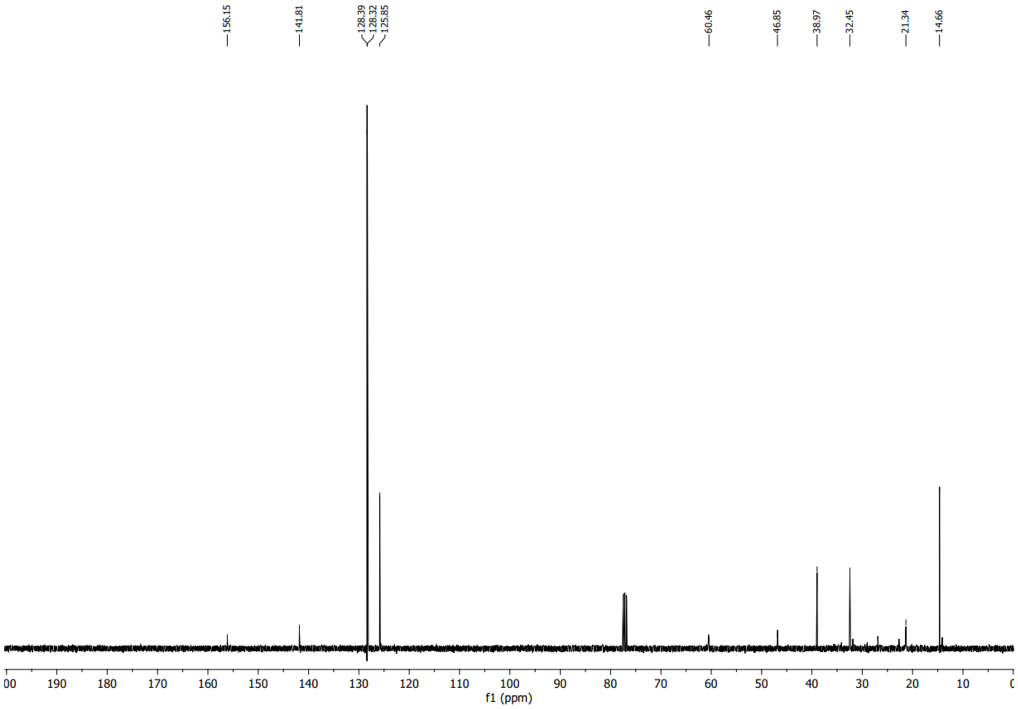

^{13}CNMR (100 MHz; $CDCl_3$) spectrum of compound **3p**.

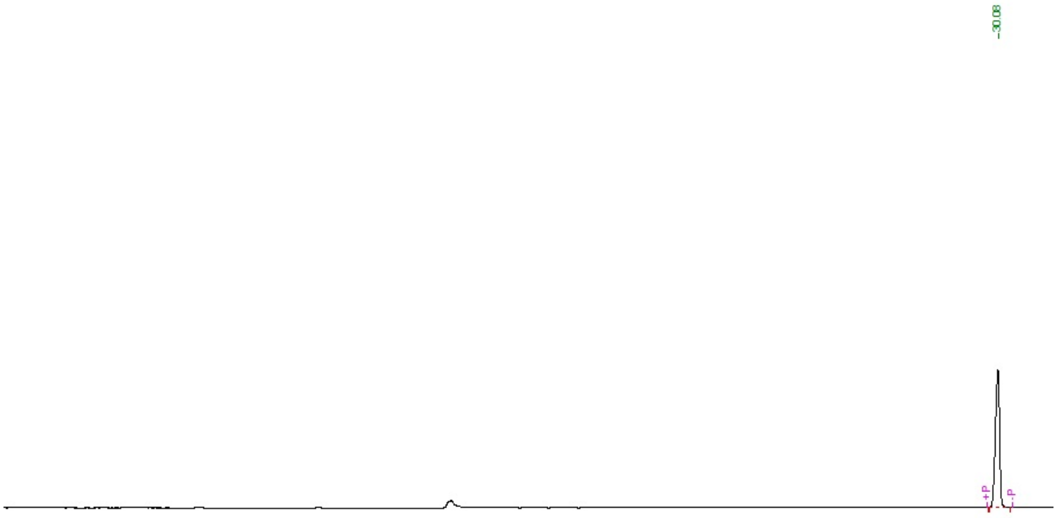

GC chromatogram (general GC method 1) of compound **3p**.

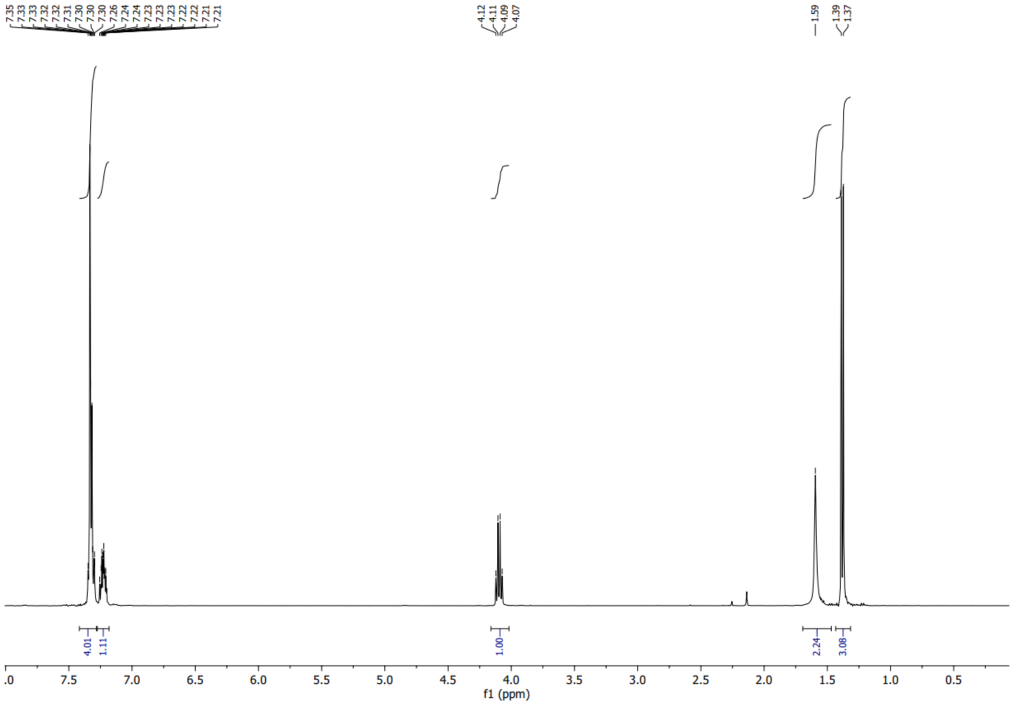

^{1}HNMR (400 MHz; $CDCl_3$) spectrum of compound **1o**.

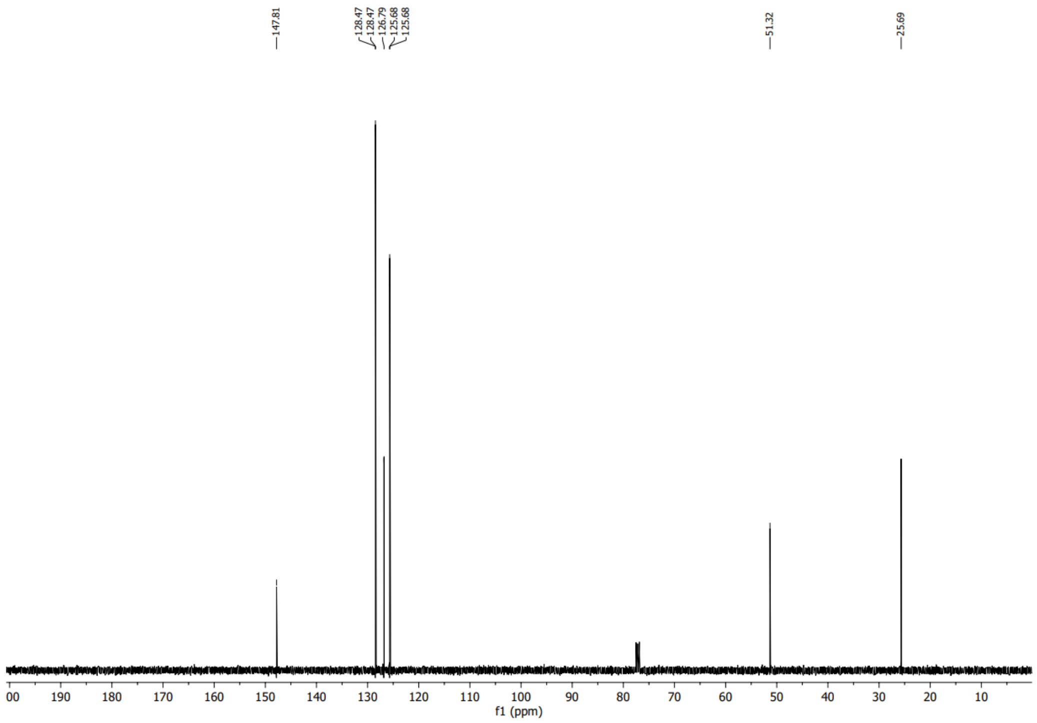

^{13}CNMR (100 MHz; $CDCl_3$) spectrum of compound **1o**.

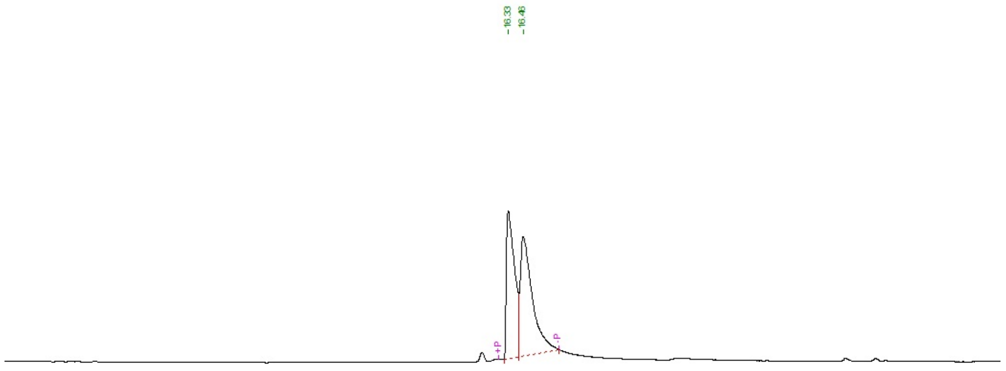

GC chromatogram (general GC method 1) of racemic compound **1o**.

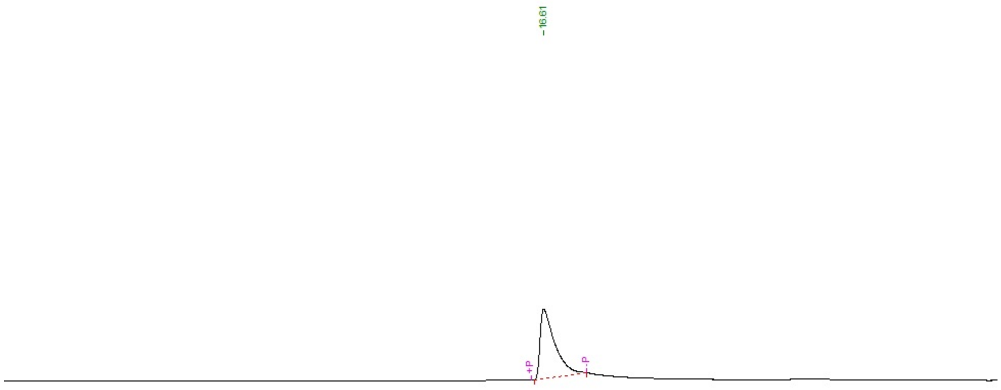

GC chromatogram (general GC method 1) of (*S*)-enantiomer of compound **1o**.

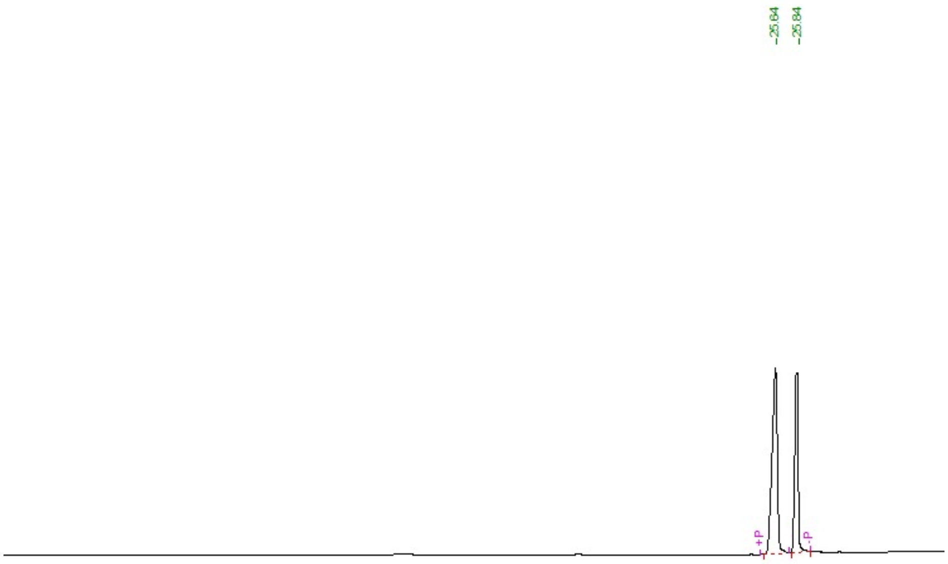

GC chromatogram (general GC method 1) of racemic compound **1o** after derivation.

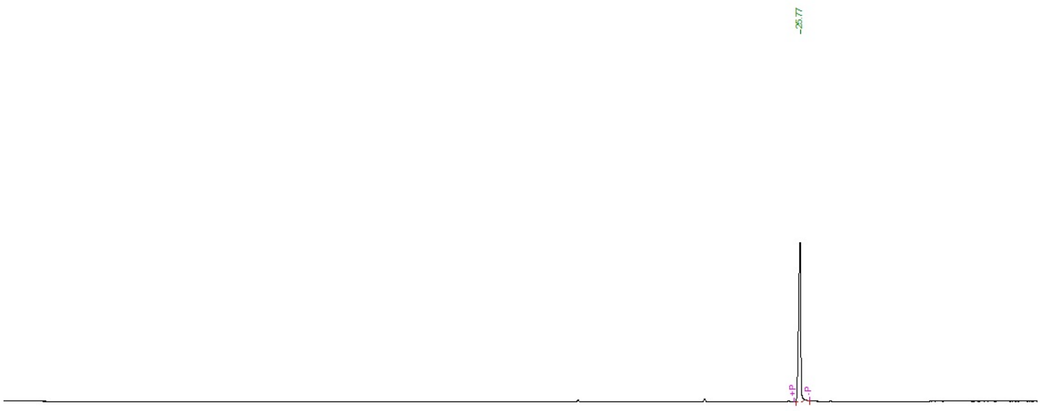

GC chromatogram (general GC method 1) (*R*)-enantiomer of compound **1o** after derivation.

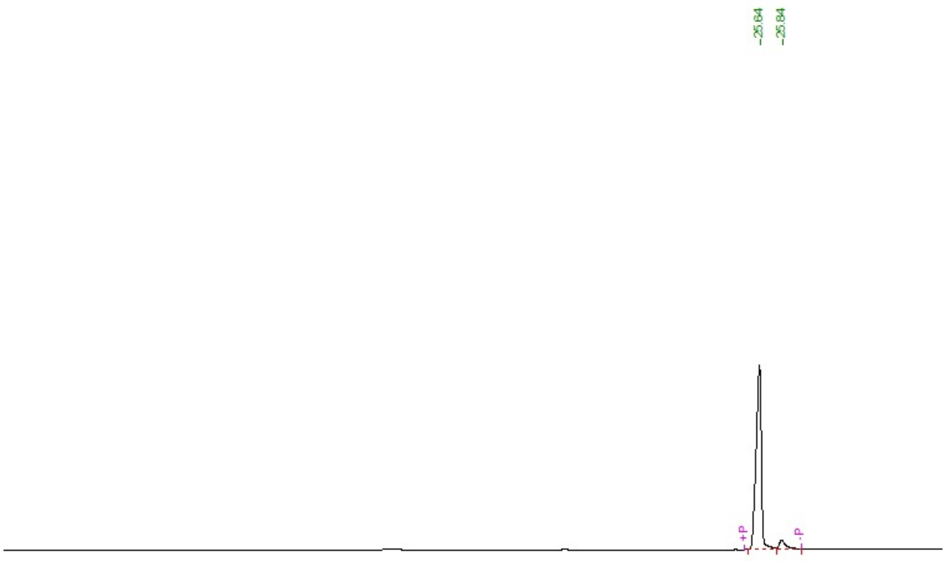

GC chromatogram (general GC method 1) (*S*)-optically enriched (90 % *ee*) compound **1o** after derivation.

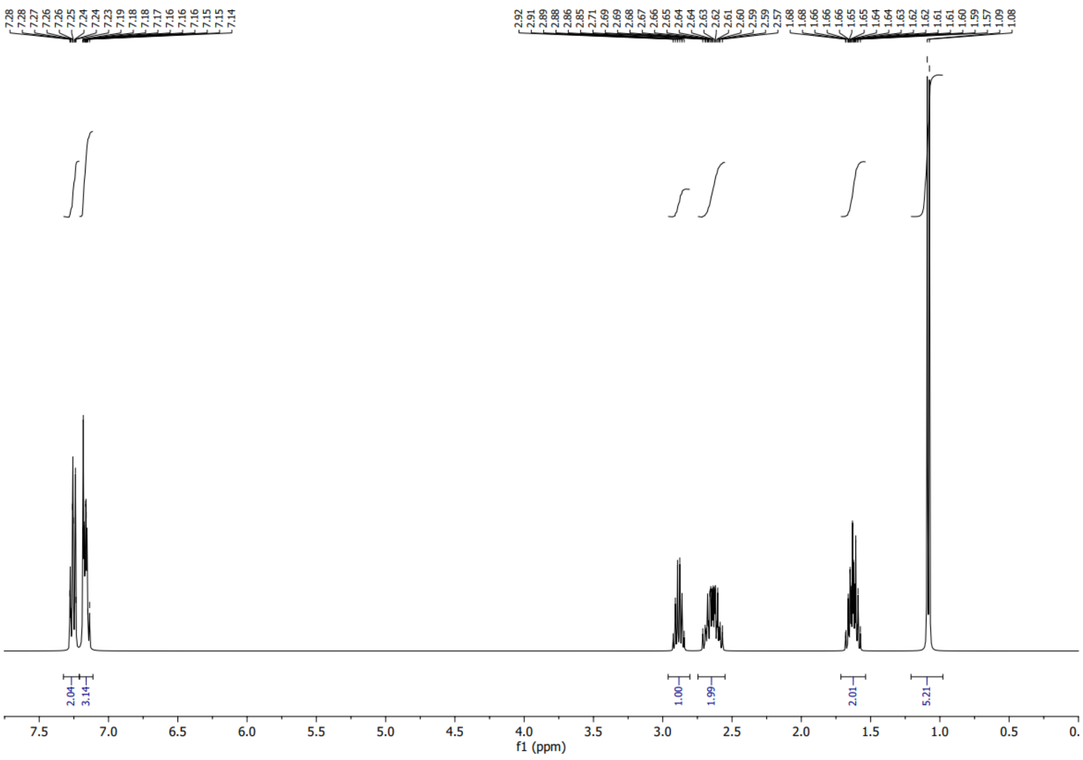

^{1}HNMR (400 MHz; CDCl3) spectrum of compound **1p**.

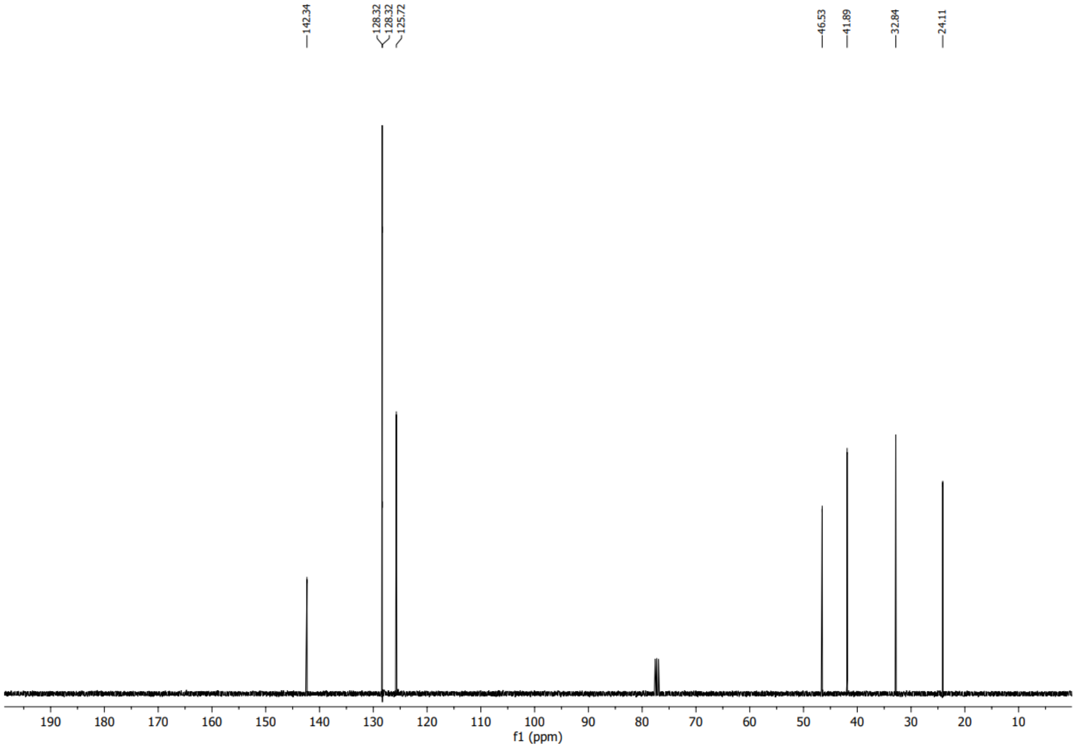

^{13}CNMR (100 MHz; CDCl3) spectrum of compound **1p**.

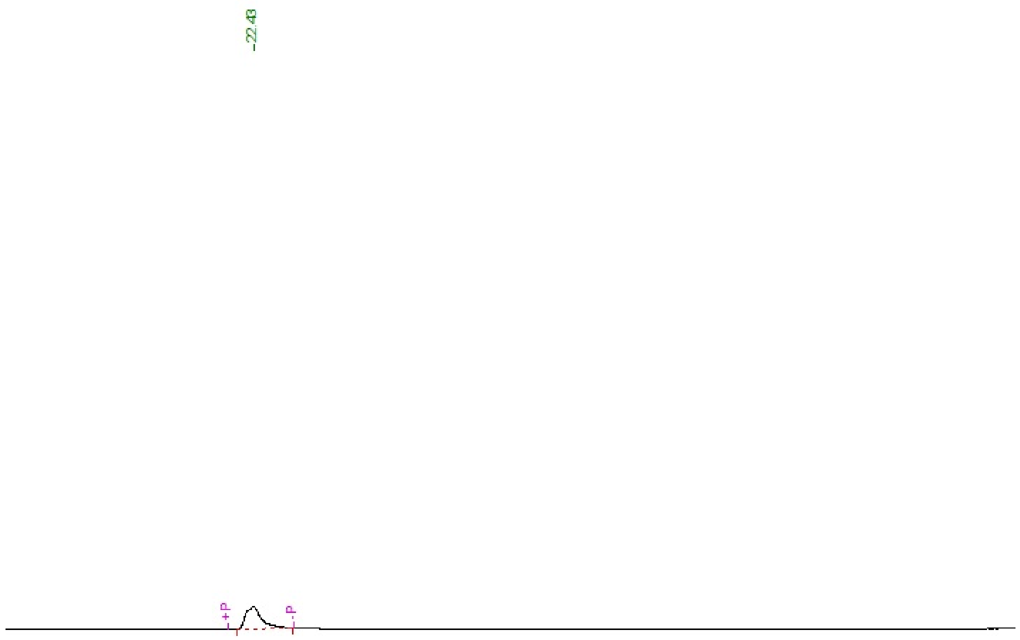

GC chromatogram (general GC method 1) of compound **1p**.

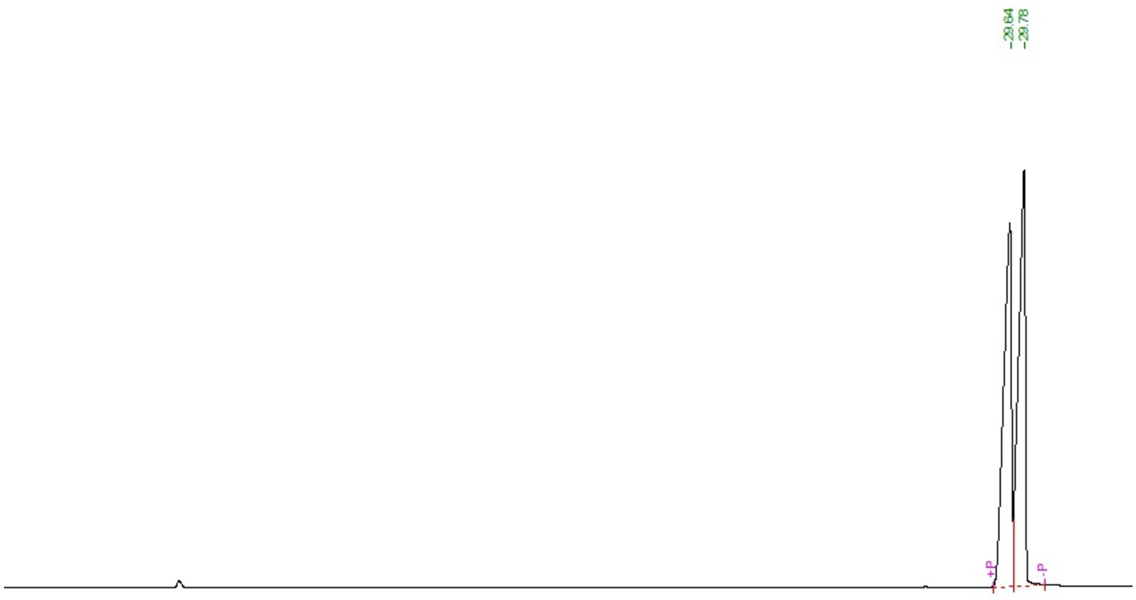

GC chromatogram (general GC method 1) of racemic compound **1p** after derivation.

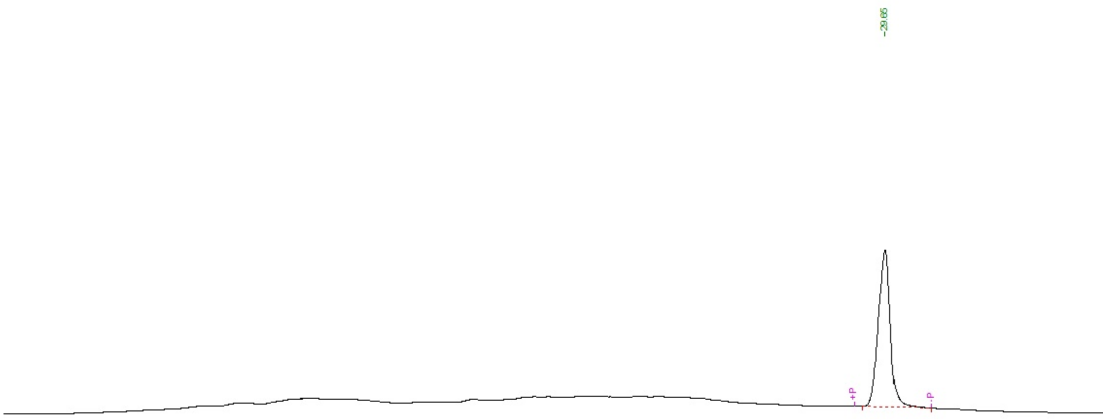

GC chromatogram (general GC method 1) (*S*)- enantiomer compound **1p** after derivation.
